# Population benefits of addressing programmatic and social determinants of gender disparities in tuberculosis in Viet Nam: A modelling study

**DOI:** 10.1371/journal.pgph.0000784

**Published:** 2022-07-14

**Authors:** Katherine C. Horton, Richard G. White, Nguyen Binh Hoa, Hai Viet Nguyen, Roel Bakker, Tom Sumner, Elizabeth L. Corbett, Rein M. G. J. Houben

**Affiliations:** 1 Department of Infectious Disease Epidemiology, London School of Hygiene and Tropical Medicine, London, United Kingdom; 2 TB Modelling Group, London School of Hygiene and Tropical Medicine, London, United Kingdom; 3 National Tuberculosis Control Programme, Hanoi, Viet Nam; 4 Department of Global Health and Amsterdam Institute of Global Health and Development, Amsterdam University Medical Centers, Amsterdam, The Netherlands; 5 Skardahl IT Solutions, Delft, The Netherlands; 6 Department of Clinical Research, London School of Hygiene and Tropical Medicine, London, United Kingdom; Pontifical Catholic University of Peru: Pontificia Universidad Catolica del Peru, PERU

## Abstract

High prevalence of infectious tuberculosis among men suggests potential population-wide benefits from addressing programmatic and social determinants of gender disparities. Utilising a sex-stratified compartmental transmission model calibrated to tuberculosis burden estimates for Viet Nam, we modelled interventions to increase active case finding, to reduce tobacco smoking, and to reduce alcohol consumption by 2025 in line with national and global targets. For each intervention, we examined scenarios differentially targeting men and women and evaluated impact on tuberculosis morbidity and mortality in men, women, and children in 2035. Active case finding interventions targeting men projected greater reductions in tuberculosis incidence in men, women, and children (16.2%, uncertainty interval, UI, 11.4–23.0%, 11.8%, UI 8.0–18.6%, and 21.5%, UI 16.9–28.5%, respectively) than those targeting women (5.2%, UI 3.8–7.1%, 5.4%, UI 3.9–7.3%, and 8.6%, UI 6.9–10.7%, respectively). Projected reductions in tuberculosis incidence for interventions to reduce male tobacco smoking and alcohol consumption were greatest for men (17.4%, UI 11.8–24.7%, and 11.0%, UI 5.4–19.4%, respectively), but still substantial for women (6.9%, UI 3.8–12.5%, and 4.4%, UI 1.9–10.6%, respectively) and children (12.7%, UI 8.4–19.0%, and 8.0%, UI 3.9–15.0%, respectively). Comparable interventions targeting women projected limited impact, with declines of 0.3% (UI 0.2%-0.3%) and 0.1% (UI 0.0%-0.1%), respectively. Addressing programmatic and social determinants of men’s tuberculosis burden has population-wide benefits. Future interventions to increase active case finding, to reduce tobacco smoking, and to reduce harmful alcohol consumption, whilst not ignoring women, should focus on men to most effectively reduce tuberculosis morbidity and mortality in men, women, and children.

## Introduction

In 2019, an estimated 10.0 million people developed tuberculosis (TB), and 1.4 million people with TB died, making TB the leading infectious cause of death worldwide [1]. Substantial gender disparities exist in the epidemiological burden of TB. Nearly twice as many cases of TB are reported among men as among women each year [1]. Prevalence surveys indicate greater disparity in the underlying burden of disease, with men accounting for 70% of adults with undiagnosed TB in low- and middle-income settings [2]. Men’s disadvantage in TB extends to diagnostic and treatment pathways, with men having less access to timely diagnosis [2,3] and worse treatment outcomes than women [4]. As a result of these disparities, over 60% of adult TB deaths occur among men each year [1], and most new infections among men, women, and children are likely attributable to contact with men [5,6].

A gender-responsive approach is needed to address factors that increase men’s risk of infection with *Mycobacterium tuberculosis* (*Mtb*) and progression to TB [6–12] whilst limiting men’s access to timely and appropriate care [13–15]. Applying a gender lens to the World Health Organization (WHO) End TB Strategy [16] highlights opportunities to address programmatic and social determinants of gender disparities in TB burden and care. Prioritisation of early diagnosis and treatment for all TB patients through integrated, patient-centred care [16] offers opportunity to confront gender disparities throughout the TB care cascade. Calls for bold policies and systems to address social determinants of TB, particularly among vulnerable groups [16], encompass strategies to tackle those social determinants that contribute to gender disparities in TB.

Viet Nam is an exemplar of the potential impact of a gender-responsive approach to TB. The country is a high TB burden country, with an estimated incidence of 176 per 100,000 in 2019 [1]. The first national TB prevalence survey in 2007 identified five male cases of undiagnosed TB for every female case, one of the highest sex ratios in the world [17]. Gaps in disease detection and reporting were significantly higher among men than women [17], and modelling indicates that men with infectious TB remained undiagnosed for one year longer than women [3]. By the second national TB prevalence survey in 2017, overall TB prevalence had fallen and gender gaps in detection and reporting had been eliminated, perhaps as a result of active case finding focused on high-risk, predominantly male populations [18]. However, prevalence remained four times higher among men than women [18], possibly due to social determinants such as tobacco smoking and harmful alcohol consumption, both well-documented proximal determinants of TB [19–22] with particularly high rates among men [23,24].

To evaluate population benefits of addressing programmatic and social determinants of gender disparities in tuberculosis, we developed a sex-stratified compartmental transmission model incorporating gendered programmatic and social determinants of *Mycobacterium tuberculosis* infection and TB disease. We assessed the potential impact of future interventions to increase active case finding, to reduce tobacco smoking, and to reduce harmful alcohol consumption, in line with national and global targets [25,26]. For each intervention, we examined scenarios differentially targeting men and women and evaluated impact on tuberculosis morbidity and mortality in men, women, and children. While our analyses focused on Viet Nam, we expect lessons on the impact of programmatic and social determinants of gender disparities in TB burden will have wider relevance across high TB burden settings.

## Methods

### Data

We developed a sex-stratified dynamic compartmental model of *Mtb* transmission and TB disease incorporating sex-disaggregated demographics and both programmatic and social determinants of gender disparities in TB in Viet Nam. Data supporting the findings of this study are available within the article and its supplemental materials (S1 Text).

The sex ratio at birth in Viet Nam is skewed towards an excess of male births [27], and life expectancy has been 8–10 years longer for women than men for the past 50 years [28]. Two-thirds of HIV infections occur among men, and fewer men living with HIV are on treatment relative to women living with HIV [29]. Social contact patterns show a high proportion of sex-assortative mixing amongst adults [30]. Gaps in TB disease detection and reporting were 72% higher among men than women in 2007 [17], and modelling indicates that men with smear-positive TB remained undiagnosed for one year longer than women [3]. In 2020, 40% of men and less than 2% of women were current tobacco smokers [23]. In the same year, the proportion of current alcohol drinkers was 48% in men and 10% in women, and men who were current alcohol drinkers consumed over five times as many standard drinks daily as women (51 grams vs. 8 grams) [24]. The adult prevalence of diabetes is similar in men and women (1% in 2000 projected to rise to 2% by 2030) [31], as is the proportion of the population that is underweight, as indicated by body mass index less than 18.5 (25% in 2002) [32], so neither of these risks was explicitly considered due to the focus on gender disparities in our model.

### Model structure

Our model is an extension of the TIME model [33] (S1 Text). The core model has four states related to *Mtb* infection and TB disease: susceptible (uninfected), latent *Mtb* infection, active smear-positive TB disease, and active smear-negative TB disease. States of infection and disease are stratified by treatment history and drug resistance, and the entire model is stratified by sex, age, and HIV and antiretroviral treatment (ART) status.

Sex-specific risks of *Mtb* infection and progression to active disease are incorporated in the model (S1 Text). Demographic data and data on HIV incidence, HIV progression, and ART uptake are sex-specific. Heterogeneous mixing reflects age- and sex-assortative mixing patterns between men (males age ≥ 15 years), women (females age ≥ 15 years), and children (both sexes age < 15 years). Increased risks of *Mtb* infection and progression to active disease as a result of tobacco smoking are applied to a proportion of men and women based on annual estimates of the prevalence of current smokers. Increased risk of progression to active disease is raised further for a proportion of men and women based on annual estimates of alcohol consumption. A constant relative risk term is applied to the risk of infection to reflect residual sex-specific effects not otherwise specified. The annual rate at which individuals enter the TB care cascade is also sex-specific.

### Model calibration

We calibrated the model to population size estimates and projections [34,35] and epidemiological calibration targets, including TB morbidity and mortality [18,36–38] and case notification rates [39] (S1 Text). Targets included indicators specific to age [36,39], sex [18,37–39], HIV status [36,39], and MDR status [40], where possible. We utilised an adaptive approximate Bayesian computation (ABC) Markov chain Monte Carlo (MCMC) method [41,42], using a modified version of the easyABC package that accepts seed parameter values [43] in R [44]. MCMC chains were thinned to select 1,000 parameter sets with posterior estimates consistent with all calibration targets. Results for the baseline calibration present median values, with uncertainty intervals (UIs) based on minimum and maximum values.

### Estimating the potential impact of future interventions

We assessed the potential impact of future interventions to increase active case finding for TB, to reduce tobacco smoking, and to reduce harmful alcohol consumption over the period 2021–2025. We modelled increased active case finding for TB by increasing the rate at which adults enter the TB care cascade such that, when implemented across the adult population, interventions result in 11,000 additional case notifications in 2021 increasing to 28,500 additional case notifications in 2025, per targets in the Viet Nam National Strategic Plan 2021–2025 [25] (S1 Text). We independently modelled interventions to reduce tobacco smoking and harmful alcohol consumption over the period 2021–2025 to reach a 30% reduction in the prevalence of current tobacco use and a 10% reduction in harmful alcohol consumption, both relative to 2015, by 2025, in line with global targets [26] (S1 Text). We assumed sigmoidal scale-up of each intervention over the period 2021–2025 with no further scale-up after 2025.

For each intervention, we examined scenarios differentially targeting men and women to evaluate the impact of meeting intervention targets only in men, meeting intervention targets only in women, and meeting intervention targets in both men and women. We calculated the projected impact of each scenario on overall TB morbidity and mortality, as well as TB burden in men, women, and children.

For each intervention scenario, we calculated the percent decline in projected TB incidence and mortality in 2035 relative to the baseline calibration. We also projected the expected number of incident TB cases and deaths averted over the period 2021–2035 for each intervention scenario relative to the baseline calibration. Results are reported as median values, with UIs based on minimum and maximum values.

### Inclusivity in global research

Additional information regarding the ethical, cultural, and scientific considerations specific to inclusivity in global research is included in the Supporting Information (S1 Checklist).

## Results

### Baseline calibration

**Fig 1:**
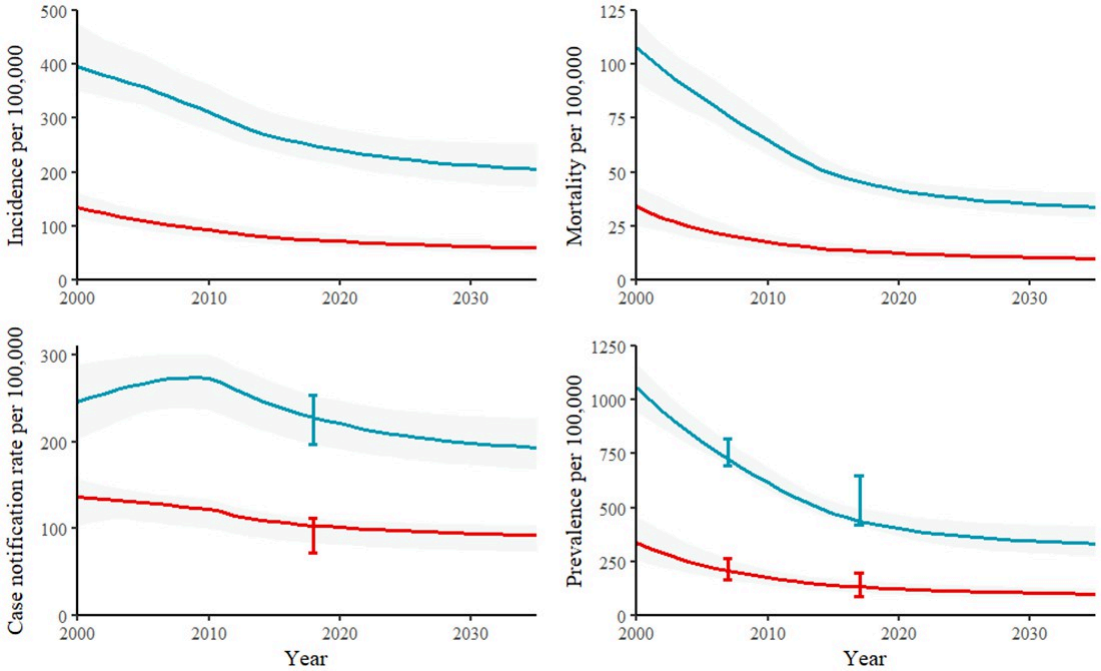
Epidemiological estimates and projections for men (light blue) and women (red) for incidence (top left), mortality (top right), case notification rate (bottom left), and prevalence (bottom right) for the calibrated model. Figures show median model estimates (line), model uncertainty (shaded area), and sex-specific calibration targets (error bars).

The calibrated model fits population size estimates and projections as well as epidemiological calibration targets (S1 Text). Model estimates reflect substantial declines in TB burden in recent decades (Fig 1). Gender disparities in TB burden changed little between 2000 and 2020 in our model (S1 Text). TB incidence, prevalence, and mortality were approximately three times higher in men than in women, while case notification rates were approximately twice as high in men as in women.

### Potential impact of future interventions

**Fig 2:**
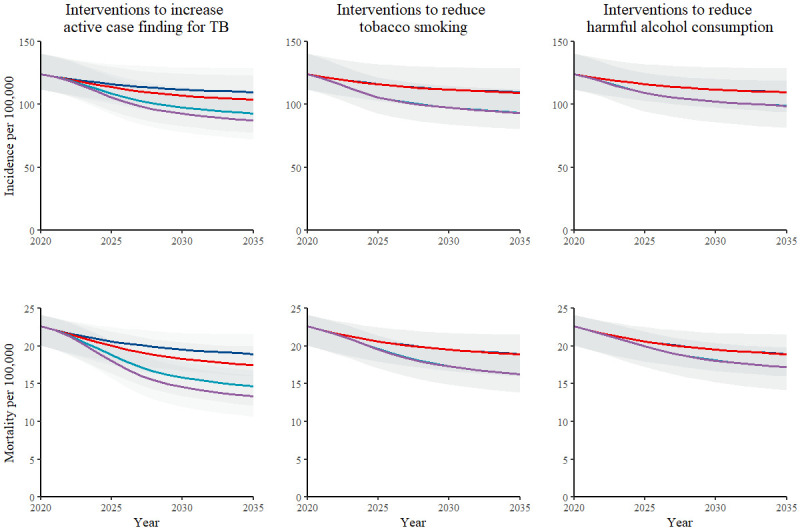
Projections for total population incidence (top) and mortality (bottom) for interventions to increase active case finding for TB (left), to reduce tobacco smoking (middle), and to reduce harmful alcohol consumption (right). Each plot shows median model estimates (lines) and model uncertainty (shaded area) for the baseline calibration (dark blue) and intervention scenario targeting men (light blue), targeting women (red), and targeting both men and women (purple).

For interventions to increase active case finding for TB, projected declines in TB incidence and mortality were greatest for scenarios targeting both men and women and higher for scenarios targeting only men than those targeting only women (Fig 2). For interventions to reduce tobacco smoking and to reduce harmful alcohol consumption, projected declines in TB incidence and mortality were similar for scenarios targeting both men and women and scenarios targeting only men; projected reductions were limited for scenarios targeting only women (Fig 2).

**Fig 3:**
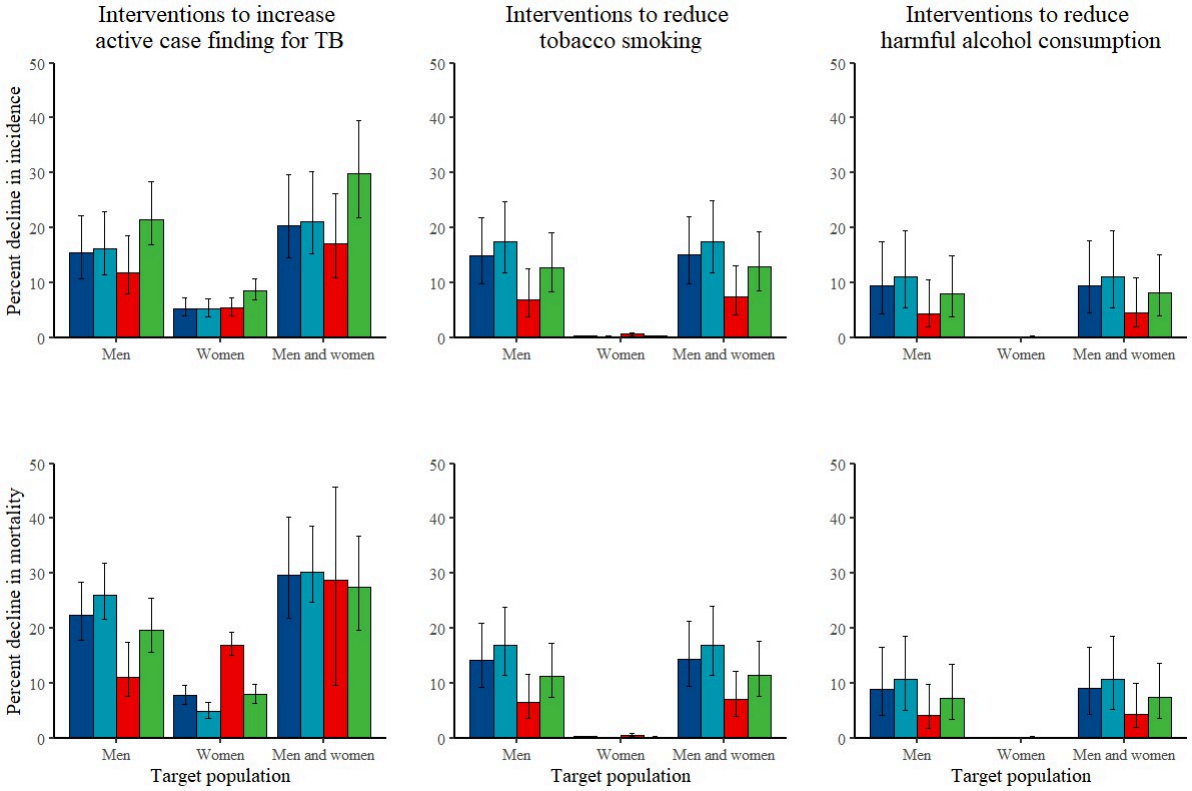
Percent decline in TB incidence (top) and mortality (bottom) in 2035, by target population (men, women, both men and women), for interventions to increase active case finding for TB (left), to reduce tobacco smoking (middle), and to reduce harmful alcohol consumption (right). Each plot shows median model estimates (bar) and model uncertainty (error bars) for the total population (dark blue), men (light blue), women (red), and children (green).

Active case finding intervention scenarios targeting only men projected greater reductions in TB incidence in men, women, and children (16.2%, uncertainty interval, UI, 11.4–23.0%, 11.8%, UI 8.0–18.6%, and 21.5%, UI 16.9–28.5%, respectively) than those targeting only women (5.2%, UI 3.8–7.1%, 5.4%, UI 3.9–7.3%, and 8.6%, UI 6.9–10.7%, respectively) (Fig 3). Projected reductions in TB mortality were greater for men and children in scenarios targeting only men (26.0%, UI 21.7–31.9%, and 19.7%, UI 15.6–25.5%, respectively) compared to scenarios targeting only women (4.9%, UI 3.7–6.6%, and 7.9%, UI 6.4–9.8%, respectively), and greater for women in scenarios targeting only women (17.0%, UI 15.1–19.3%) compared to scenarios targeting only men (11.1%, UI 7.6–17.4%).

For interventions to reduce tobacco smoking and to reduce harmful alcohol consumption, projected reductions in TB incidence for scenarios targeting only men were greatest for men (17.4%, UI 11.8–24.7%, and 11.0%, UI 5.4–19.4%, respectively), but still substantial for women (6.9%, UI 3.8–12.5%, and 4.4%, UI 1.9–10.6%, respectively) and children (12.7%, UI 8.4–19.0%, and 8.0%, UI 3.9–15.0%, respectively) (Fig 3). Projected declines in mortality for scenarios targeting only men were similarly distributed across men, women, and children. Comparable interventions targeting only women projected limited impact on TB incidence and mortality.

Between 2021 and 2035, the baseline calibration projected a total estimated 1.68 million (UI 1.47–1.93 million) incident TB cases and 300,000 (UI 250,000–330,000) TB deaths. The greatest proportion of incident cases and deaths were projected to be averted by interventions targeting both men and women. Across interventions, scenarios targeting only men are projected to avert a higher percentage of incident cases and deaths than scenarios targeting only women (Table 1). Distributions of incident cases and deaths averted in men, women, and children are similar to distributions of reductions in incidence and mortality in 2035 across those populations.

**Table 1:** Percent of projected incident cases averted and deaths averted over 2021–2035 for interventions to increase active case finding for TB, to reduce tobacco smoking, and to reduce harmful alcohol consumption by target population.

Targeted population	Percent of projected incident cases averted	Percent of projected deaths averted
Interventions to increase active case finding for TB		
Men	9.4% (6.6–13.1%)	13.4% (11.0%-16.8%)
Women	3.2% (2.4–4.2%)	4.5% (3.7%-5.4%)
Men and women	12.7% (8.4–19.2%)	18.1% (12.5%-25.6%)
Interventions to reduce tobacco smoking		
Men	10.2% (6.9–14.9%)	8.1% (5.4%-12.1%)
Women	0.2% (0.1–0.2%)	0.1% (0.1%-0.2%)
Men and women	10.3% (7.0–15.0%)	8.2% (5.5%-12.2%)
Interventions to reduce harmful alcohol consumption		
Men	6.6% (3.1–11.5%)	5.2% (2.4%-9.1%)
Women	0.1% (0.0–0.1%)	0.0% (0.0%-0.1%)
Men and women	6.6% (3.1–11.5%)	5.2% (2.4%-9.1%)

## Discussion

Our analyses using mathematical modelling shows that addressing programmatic and social determinants of gender disparities in TB burden and care in Viet Nam will have population-wide benefits. Interventions to increase active case finding for TB, to reduce tobacco smoking, and to reduce harmful alcohol consumption are projected to reduce TB morbidity and mortality in men, women, and children, especially when interventions are targeted towards men.

Over the past decade, the scale up of active case finding activities in Viet Nam has closed the gender gap in access to diagnosis and treatment by focusing on high risk populations including predominantly male populations such as prison inmates and coal miners [18]. Our analyses show clear population-wide benefits of efforts to further improve access to TB care, particularly for interventions targeting men. Future active case finding interventions, whilst not ignoring women, should focus efforts on men to most effectively reduce TB morbidity and mortality in men, women, and children. Appropriately, the National Strategic Plan for the National TB Programme in Viet Nam highlights vulnerable, predominantly male populations including tobacco smokers, alcohol consumers, those with silicosis, inmates and staff in correctional facilities, and migrants. Active case finding strategies to reach these and other hard-to-reach male populations must consider gendered barriers that disadvantage men in access TB care, notably financial and work-related concerns, stigma, and negative masculinities. Evidence from other settings indicates a range of strategies that offer convenient access to diagnosis and treatment may be needed across community [45–47], health care [48], occupational [49,50], transport [51], and leisure settings, though these approaches must be tailored to reach men in different geographic settings, age groups, and socioeconomic strata within Viet Nam.

Efforts are also needed to address social determinants of gender disparities in TB that contribute to men’s disproportionate incidence of disease. Our analyses show that the benefit of interventions to reduce tobacco smoking and to reduce harmful alcohol consumption comes almost entirely from their implementation in men. When focusing on TB morbidity and mortality, reducing tobacco smoking and harmful alcohol consumption in men has a greater impact on women’s health than reducing tobacco smoking and harmful alcohol consumption in women. The prevalence of tobacco smoking and alcohol consumption are, respectively, 20 and 10 times higher among men than women, and while the prevalence of tobacco smoking has declined slightly in recent decades [52], alcohol consumption has increased substantially [24]. Whilst efforts to change social determinants of TB are challenging for a national TB programme to instigate, such efforts are in line with the WHO End TB Strategy, as well as the multidimensional approach outlined in the Sustainable Development Goals [53]. Although the potential impact on TB epidemiology is less pronounced for interventions to reduce tobacco smoking and to reduce harmful alcohol consumption, compared to interventions to increase active case finding, the wider benefits of these interventions should make them appealing to TB programmes as well as non-communicable disease programmes.

We have not specified intervention strategies given the limited evidence on effective strategies to reach men with interventions examined here. For the same reason, we have considered neither the economic costs nor operational feasibility of interventions. Our aim was to examine the potential population-wide benefits of these interventions, focusing on the relative impact of strategies differentially targeting the sexes, in order to highlight the potential and guide the development of future interventions. Further work will be needed to identify acceptable and feasible, effective and cost-effective strategies.

Our work has several limitations. Few sex-stratified data points were available for model calibration, emphasising the need for further disaggregation of routine surveillance data and TB burden estimates by sex. Similarly, we were not able to examine more nuanced age-sex interactions in natural history and gendered risks, including tobacco smoking and harmful alcohol consumption, due to the lack of empirical data. The sex-specific risks we have included in the model are not comprehensive, and we have not explored their interactions. However, to our knowledge, this study presents the first comprehensive sex-stratified dynamic transmission model of TB to explore potential solutions for the substantial and consistent sex disparities in TB burden across low- and middle-income countries [2,54]. By incorporating programmatic and social determinants of gender disparities in TB, we have been able to identify context-specific drivers of those disparities in TB burden and generate TB burden estimates and trends not currently available with sex disaggregation.

Gender disparities in the epidemiological burden of TB are pronounced in Viet Nam and globally. As countries strive to reduce TB morbidity and mortality in line with the End TB Strategy and Sustainable Development Goals [16,53], a gender-responsive approach that considers programmatic and social determinants of TB is essential to accelerate progress toward these targets. Our work adds evidence that future interventions to increase active case finding, to reduce tobacco smoking, and to reduce harmful alcohol consumption, whilst not ignoring women, should focus on men to most effectively reduce TB morbidity and mortality in men, women, and children.

## Supporting information

S1 Checklist Inclusivity in global research checklist. (DOCX)

S1 Text Detailed modelling methods. (DOCX)
