# Supplementary material for: Population benefits of addressing programmatic and social determinants of gender disparities in tuberculosis in Viet Nam: A modelling study
Source: PLOS Glob Public Health. 2022 Jul 14;2(7):e0000784. doi: 10.1371/journal.pgph.0000784 (PMC10021793; doi:10.1371/journal.pgph.0000784)
Supplement: S1 Text — (DOCX) [file pgph.0000784.s002.docx]

Population benefits of addressing programmatic and social determinants
of gender disparities in tuberculosis in Viet Nam: a modelling study

Supplemental materials: Detailed modelling methods

1 Model structure 2

1.1 Tuberculosis model 2

1.2 Demographic model 6

1.3 HIV model 7

2 Model equations 9

3 Model parameters 14

3.1 Force of infection 14

3.2 Progression to active disease 17

3.3 Infectious disease 25

3.4 Self-cure 26

3.5 Care cascade 26

3.6 Mortality 30

3.7 MDR 32

4 Model implementation 33

5 Calibration 34

5.1 Population calibration 34

5.2 Epidemiological calibration 34

6 Calibrated model 36

6.1 Posterior parameters 36

6.2 Population calibration 39

6.3 Epidemiological calibration 39

7 References 48

1 Model structure

1.1 Tuberculosis model

We developed a sex-stratified dynamic compartmental model of *Mtb* transmission and TB disease based on a model previously published by Houben et al. [1]. The core TB model has four states related to *Mtb* infection and TB disease: susceptible, latent infection, smear-positive TB disease, and smear-negative TB disease, as shown in Figure 1. (There is no compartment for post-preventive therapy, unlike the model by Houben et al.)


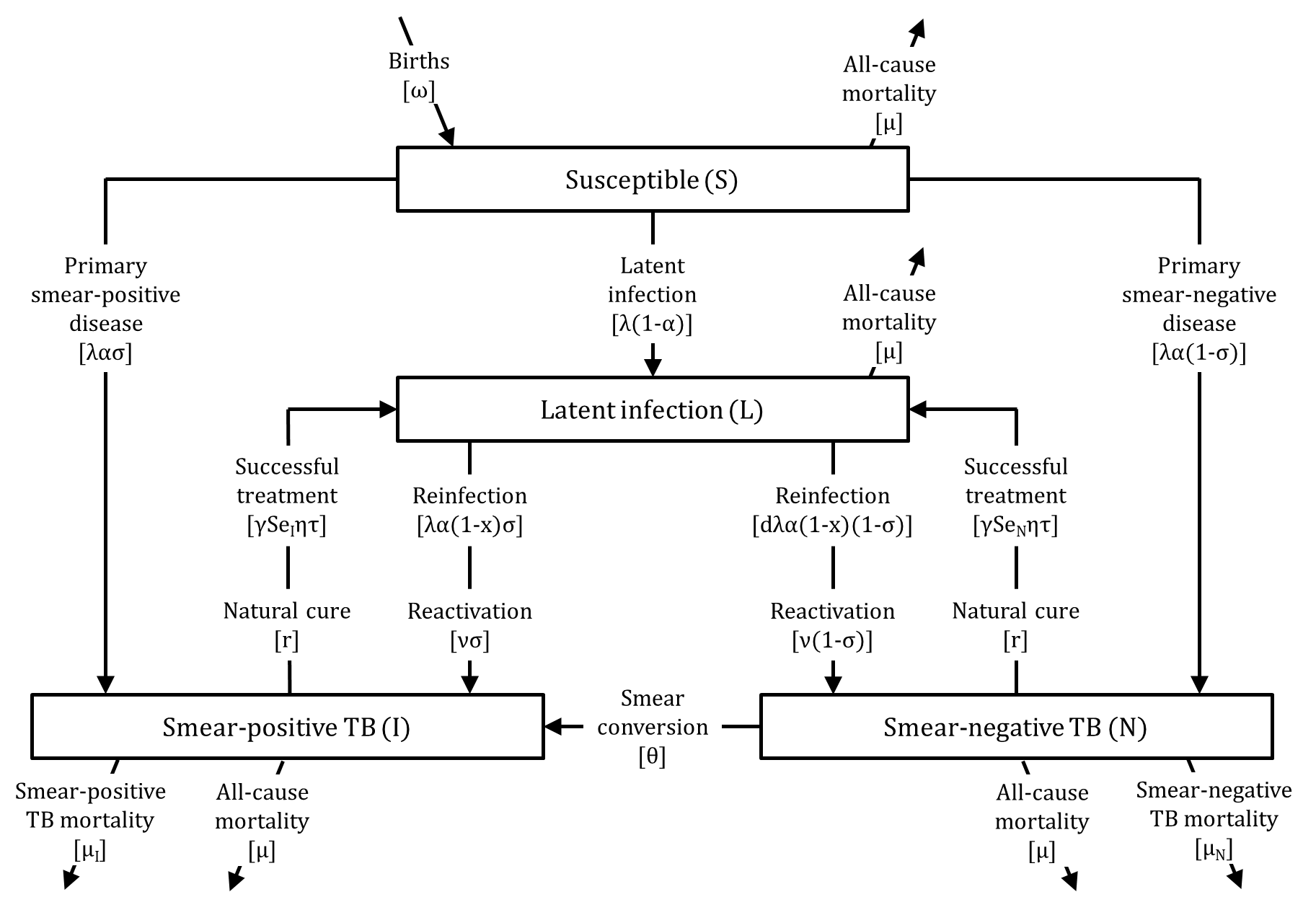


Figure 1: TB model structure

Individuals enter the susceptible compartment (S) at birth and are at risk of all-cause mortality throughout the model.

Susceptible individuals (S) are at annual risk of infection *λ*. Proportion α of infected individuals progress to disease, with proportion σ developing smear-positive TB (I) and proportion (1-σ) develop smear-negative (N) disease. The remaining proportion *(1-α)* are latently infected (L). Individuals with latent infection are at risk of progressing to disease through reinfection, at rate *λ*α*(1-x)*, where *x* is the proportion of protection provided by previous infection against progression to disease following reinfection, with proportion σ developing smear-positive disease and proportion *(1-σ)* developing smear-negative disease. Latently infected individuals also progress to disease through reactivation at rate *ν*, with proportion *σ* developing smear-positive disease and proportion *(1-σ)* developing smear-negative disease.

Individuals with smear-negative disease convert to smear-positive disease at rate *θ*. Individuals with smear-positive or smear-negative disease experience TB-associated mortality at rates *μ_I_* and *μ_N_*, respectively.

Individuals who recover from TB disease return to the latent infection compartment where they are at risk of reactivation or reinfection as described above. Individuals recover through natural cure at rate *r* or through successful treatment following access to TB care, diagnosis, and linkage to care. Individuals are screened for disease at rate *γ*, the inverse of time to presentation, with individuals with smear-negative disease screened at a lower rate *d*γ* and individuals with neither smear-positive nor smear-negative disease screened at lower rate *h*********γ*. Screened individuals are diagnosed based on the net sensitivity *Se* and specificity *Sp* of the diagnostic algorithm, the proportion *ψ* who receive drug-susceptibility testing (DST), and the sensitivity and specificity of DST. Proportion *η* of those diagnosed with TB are linked to treatment, and proportion *τ* successfully complete treatment.

Infection and disease compartments (L, I, and N) are stratified based on treatment history (treatment naïve, indicated by subscript *X*, and previously treated, subscript *P*) and drug resistance status (drug-susceptible, subscript *S*, and multidrug-resistant, MDR, subscript *R*).

Individuals move from the treatment naïve stratum to the previously treated stratum after completing treatment or after initiating unsuccessful treatment, as shown in Figure 2 and detailed elsewhere [1]. Those who complete treatment move from I_X_ and N_X_ to L_P_, while those who initiate unsuccessful treatment move from I_X_ to I_P_ and N_X_ to N_P_.


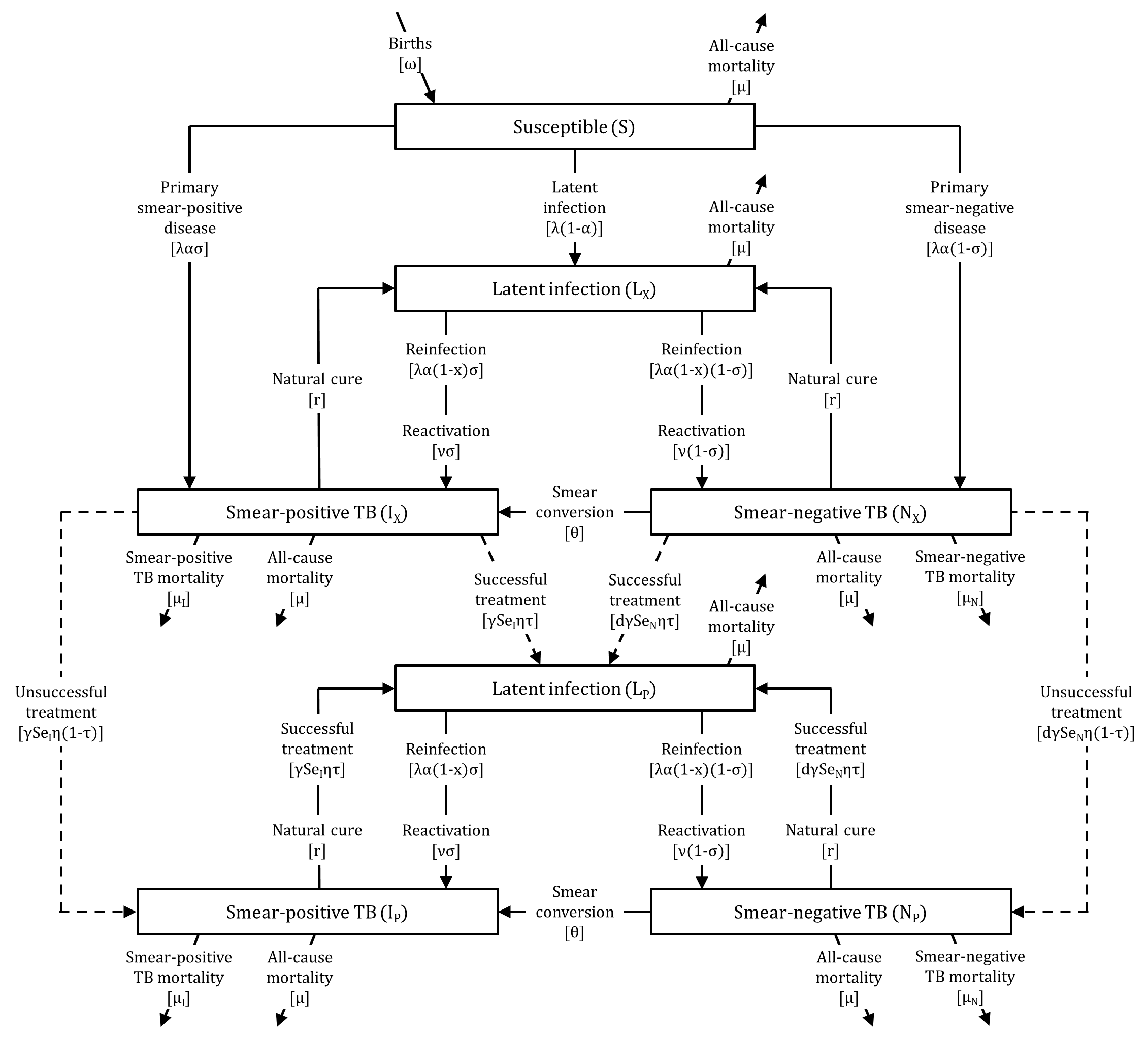


Figure 2: Model structure with treatment history strata. Top of the figure shows treatment naïve stratum (subscript X); bottom shows previously treated stratum (subscript P). Transitions between treatment naïve and previously treated strata are indicated by dashed lines.

MDR is acquired either by transmission of an MDR strain or development during treatment, as shown in Figure 3 and detailed elsewhere [1]. MDR transmission is specified by a separate annual risk of infection *λ_R_* adjusted by *φ* to indicate the relative fitness of MDR strains. Mixed infections are not explicitly modelled. Superinfections that rapidly progress to disease move to the disease compartment matching the drug resistance profile of the superinfecting strain (I_S_, N_S_, I_R_, or N_R_), regardless of the resistance profile of the previous infection. The movement of individuals with latent superinfection is determined by parameter $\iota=\frac{\varphi}{1+\varphi}$ such that among individuals with latent drug sensitive infection who are reinfected with an MDR strain, proportion *ι* move from L_S_ to L_R_ and proportion *(1-ι)* remain in L_S_, and among individuals with latent MDR infection who are reinfected with a drug resistant strain, proportion *(1-ι)* move from L_R_ to L_S_ and proportion *ι* remain in L_R_. Individuals with drug susceptible TB who are diagnosed and initiate treatment develop MDR at rate ξ.


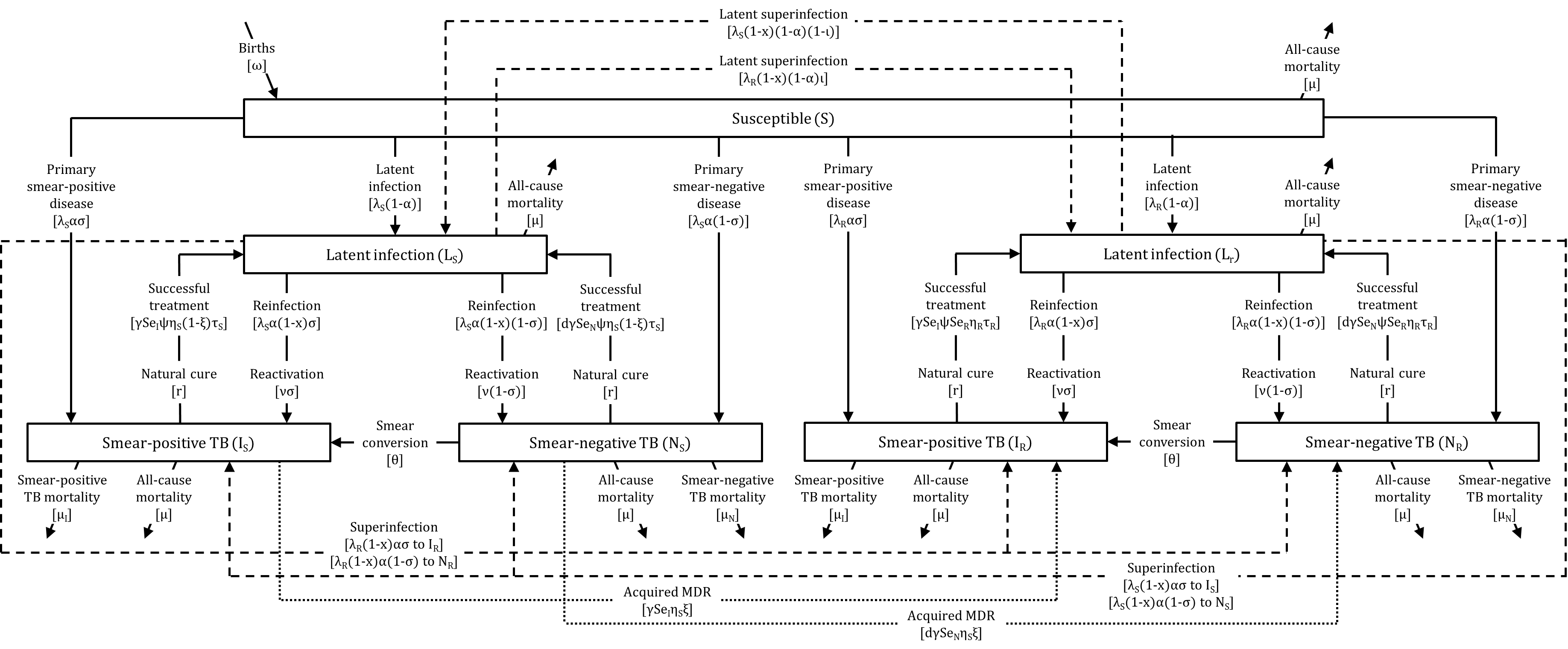


**Figure 3: Model diagram illustrating drug susceptible and MDR strata. Left side of the diagram shows drug susceptible stratum (subscript S); right side shows MDR stratum (subscript R). Dashed lines indicate superinfections. Dotted lines indicated acquired resistance.**

The full model is stratified by sex (male and female), age (five-year age groups, i.e., 0-4, 5-9, ...75-79, ≥80), and HIV status (positive and negative), with HIV-positive individuals further stratified by CD4 count (<50, 50-99, 100-199, 200-249, 250-349, 350-499, and ≥500 cells/μL) and duration of anti-retroviral treatment (ART) (none, 0-6, 7-12, >12 months).

Model equations are provided in Section 0, and model parameters are provided in Section 0.

1.2 Demographic model

The demographic model is designed to reflect the demographic projection model (DemProj) of the Spectrum software suite [2]. The demographic model accounts for births, migration, and all-cause mortality and divides the population across two sex strata (male and female) and 17 age strata (five year age groups, i.e., 0-4, 5-9, ..., 75-79, ≥80). Sex is assumed constant through an individual’s lifetime.

Births are modelled using the crude birth rate based on United Nations (UN) Population Division estimates [3], acknowledging the sex distribution of new births according to UN World Population Prospects estimates (Figure 4) [4]. New births are added to the susceptible compartment of the TB model. Aging is modelled such that one-fifth of the population of each five-year age group moves to the subsequent age group each year.


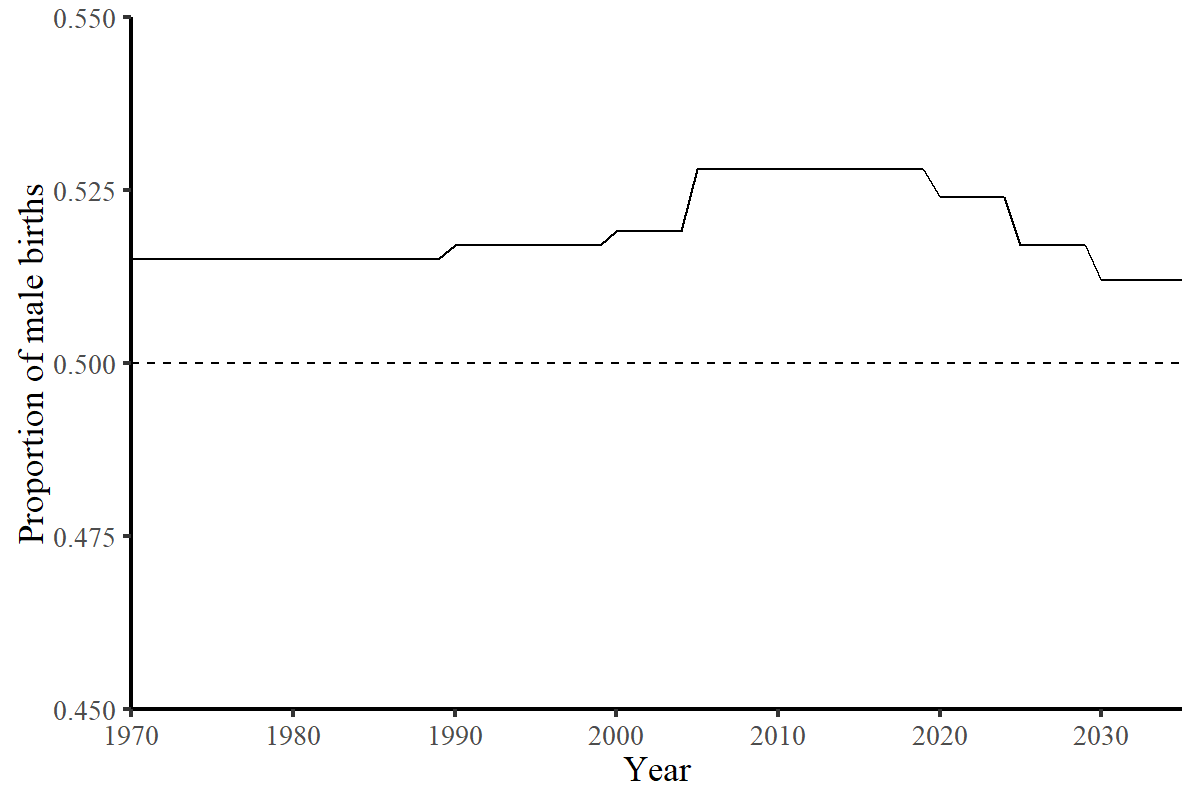


Figure 4: Proportion of male births (Dashed line at 0.5 for reference)

Migration is calculated by age and sex for each year according to DemProj estimates. Migration is considered independent of TB or HIV disease status, and migrants are distributed across disease states based on the relative size of each compartment.

All-cause mortality is modelled using rates derived from UN Population Division life tables [3]. Because TB and HIV deaths are included in background mortality rates, duplicate TB- and HIV-associated deaths are removed at each time point.

1.3 HIV model

The HIV model is designed to reflect the AIDS Impact Model (AIM) of the Spectrum software suite [2]. Individuals who are HIV-positive are categorized into seven stages based on CD4 count (<50, 50-99, 100-199, 200-249, 250-349, 350-499, and >500 cells/μL) and into four stages based on ART duration (none, 0-6, 7-12, >12 months). HIV incidence and progression (the latter indicated by CD4 count), ART initiation and duration, and HIV-associated mortality are modelled as described elsewhere [1].

HIV incidence is based on age- and sex-specific incidence estimates from AIM [2]. HIV incidence is considered independent of TB status. New infections are assigned to CD4 categories according to age-specific distributions from AIM (Table 1).

Table 1: Proportional distribution of new HIV infections by age group and CD4 category

| CD4 count | Age group (years) | | | | |
| --- | --- | --- | --- | --- | --- |
|  | 0-14 | 15-24 | 25-34 | 35-44 | 45+ |
| >500 | 64.3 | 64.3 | 60.7 | 58.5 | 55.2 |
| 350-500 | 35.7 | 35.7 | 39.3 | 41.5 | 44.8 |
| 250-349 | 0 | 0 | 0 | 0 | 0 |
| 200-249 | 0 | 0 | 0 | 0 | 0 |
| 100-199 | 0 | 0 | 0 | 0 | 0 |
| 50-99 | 0 | 0 | 0 | 0 | 0 |
| <50 | 0 | 0 | 0 | 0 | 0 |

Individuals progress through CD4 categories at age- and CD4-specific rates, which are derived from risks used in AIM (Table 2) [2].

Table 2: Rates of progression through CD4 count categories by age group and CD4 category

| CD4 count | Age group (years) | | | | |
| --- | --- | --- | --- | --- | --- |
|  | 0-14 | 15-24 | 25-34 | 35-44 | 45+ |
| >500 | 0.298 | 0.117 | 0.147 | 0.183 | 0.213 |
| 350-500 | 0.239 | 0.223 | 0.240 | 0.355 | 0.535 |
| 250-349 | 0.183 | 0.294 | 0.452 | 0.581 | 0.855 |
| 200-249 | 0.183 | 0.508 | 1.087 | 1.250 | 1.818 |
| 100-199 | 0.130 | 0.214 | 0.637 | 0.676 | 0.952 |
| 50-99 | 0.130 | 0.348 | 1.449 | 1.449 | 2.000 |

HIV-associated mortality rates in the absence of ART are based on age- and CD4-specific rates from AIM (Table 3) [2]. Mortality rates in ages 0-4 years are assumed eight times those in ages 5-14 years [1]. Duplicate TB- and HIV-associated deaths are removed at each time point.

Table 3: HIV mortality rates (in the absence of ART) by age group and CD4 category

| CD4 count | Age group (years) | | | | | |  |
| --- | --- | --- | --- | --- | --- | --- | --- |
|  | 0-4 | 5-14 | 15-24 | 25-34 | 45-44 | 45-54 | |
| >500 | 0.312 | 0.039 | 0.005 | 0.004 | 0.005 | 0.005 | |
| 350-500 | 0.382 | 0.048 | 0.011 | 0.01 | 0.013 | 0.013 | |
| 250-349 | 0.466 | 0.058 | 0.026 | 0.026 | 0.036 | 0.032 | |
| 200-249 | 0.466 | 0.058 | 0.061 | 0.069 | 0.096 | 0.08 | |
| 100-199 | 0.569 | 0.071 | 0.139 | 0.185 | 0.258 | 0.203 | |
| 50-99 | 0.569 | 0.071 | 0.321 | 0.499 | 0.691 | 0.513 | |
| <50 | 0.569 | 0.071 | 0.737 | 1.342 | 1.851 | 1.295 | |

The number of individuals who should be on ART is calculated from age- and sex-specific estimates of the number of individuals on ART and the number of individuals in need of ART from AIM [2], based on the CD4 threshold for ART initiation (Table 4). ART coverage does not take into account risk of HIV infection during TB disease nor diagnosis of TB disease as an indicator of ART eligibility.

Table 4: CD4 threshold for ART initiation by year

| Year | CD4 threshold (cells/μL) |
| --- | --- |
| 1970-2009 | 200 |
| 2010-2050 | 350 |

The number of individuals who start ART within a given time step is calculated as the difference between the number of individuals who should be on ART and the number who are currently on ART, plus the number who will die on ART during that time step. The distribution of individuals starting ART across CD4 categories is based on the proportion of individuals eligible for ART in each CD4 category and the proportion of deaths among individuals eligible for ART (but not currently on ART) in each CD4 category.

HIV-associated mortality rates for individuals on ART, by age, sex, CD4 count at ART initiation, and duration of ART, are taken from AIM [2].

2 Model equations

Transitions between compartments are defined as follows. Uppercase subscripts indicate states of and parameters specific to infection and disease strata based on treatment history (treatment-naïve, indicated by subscript *X*, and previously treated, subscript *P*) and drug resistance status (drug-susceptible, subscript *S*, and multidrug-resistant, MDR, subscript *R*). Lowercase subscripts denote states of and parameters specific to strata by sex (subscript *g*), age (subscript *a*), HIV status (subscript *h*), and duration of ART (subscript *k*), and time-step (subscript *t*).

$\frac{dS_{g,a,h,k,t}}{\mathrm{dt}} =-\left( \lambda_{S_{g,a,t}}+\lambda_{R_{g,a,t}} \right)*S_{g,a,h,k,t}$ ^[[1]](#footnote-2)^

$\frac{dL_{\mathrm{SX}_{g,a,h,k,t}}}{\mathrm{dt}}=\left( \lambda_{S_{g,a,t}}\left( 1-\alpha_{g,a,h,k,t} \right) \right)*S_{g,a,h,k,t}$

$-\left( \lambda_{S_{g,a,t}}\left( 1-x_{a,h,k} \right)\alpha_{g,a,h,k,t}+\lambda_{R_{g,a,t}}\left( 1-x_{a,h,k} \right)\alpha_{g,a,h,k,t}+\lambda_{R_{g,a,t}}\left( 1-x_{a,h,k} \right)\left( 1-\alpha_{g,a,h,k,t} \right)\iota+\nu_{g,a,h,k,t} \right)*L_{{SX}_{g,a,h,k,t}}$

$+\left( r_{h} \right)*I_{\mathrm{SX}_{g,a,h,k,t}}$

$+\left( r_{h} \right)*N_{\mathrm{SX}_{g,a,h,k,t}}$

$+\left( \lambda_{S_{g,a,t}}\left( 1-x_{a,h,k} \right)\left( 1-\alpha_{g,a,h,k,t} \right)\left( 1-\iota\right) \right)*L_{\mathrm{RX}_{g,a,h,k,t}}$

$\frac{dI_{\mathrm{SX}_{g,a,h,k,t}}}{\mathrm{dt}} =\left( \lambda_{S_{g,a,t}}\alpha_{g,a,h,k,t}\sigma_{a,h} \right)*S_{g,a,h,k,t}$

$+\left( \lambda_{S_{g,a,t}}\left( 1-x_{a,h,k} \right)\alpha_{g,a,h,k,t}\sigma_{a,h}+\nu_{g,a,h,k,t}\sigma_{a,h} \right)*L_{\mathrm{SX}_{g,a,h,k,t}}$

$-\left( r_{h}+\gamma_{g,a,t}\mathrm{Se}_{I_{h,t}}\psi_{X_{t}}\mathrm{Sp}_{R_{t}}\eta_{S_{t}}+\gamma_{g,a,t}\mathrm{Se}_{I_{h,t}}\left( 1-\psi_{X_{t}} \right)\eta_{S_{t}}+\gamma_{g,a,t}\mathrm{Se}_{I_{h,t}}\psi_{X_{t}}\left( 1-\mathrm{Sp}_{R_{t}} \right)\eta_{R_{t}} \right)*I_{\mathrm{SX}_{g,a,h,k,t}}$

$+\left( \theta_{h} \right)*N_{\mathrm{SX}_{g,a,h,k,t}}$

$+\left( \lambda_{S_{g,a,t}}\left( 1-x_{a,h,k} \right)\alpha_{g,a,h,k,t}\sigma_{a,h} \right)*L_{\mathrm{RX}_{g,a,h,k,t}}$

$\frac{dN_{\mathrm{SX}_{g,a,h,k,t}}}{\mathrm{dt}} =\left( \lambda_{S_{g,a,t}}\alpha_{g,a,h,k,t}\left( 1-\sigma_{a,h} \right) \right)*S_{g,a,h,k,t}$

$+\left( \lambda_{S_{g,a,t}}\left( 1-x_{a,h,k} \right)\alpha_{g,a,h,k,t}\left( 1-\sigma_{a,h} \right)+\nu_{g,a,h,k,t}\left( 1-\sigma_{a,h} \right) \right)*L_{\mathrm{SX}_{g,a,h,k,t}}$ ^[[2]](#footnote-3)^

$-\left( r_{h}+d_{N}\gamma_{g,a,t}\mathrm{Se}_{N_{h,t}}\psi_{X_{t}}{\mathrm{Sp}_{R_{t}}\eta}_{S_{t}}+d_{N}\gamma_{g,a,t}\mathrm{Se}_{N_{h,t}}\left( 1-\psi_{X_{t}} \right)\eta_{S_{t}}+d_{N}\gamma_{g,a,t}\mathrm{Se}_{N_{h,t}}\psi_{X_{t}}{\left( 1-\mathrm{Sp}_{R_{t}} \right)\eta}_{R_{t}}+\theta_{h} \right)*N_{\mathrm{SX}_{g,a,h,k,t}}$

$+\left( \lambda_{S_{g,a,t}}\left( 1-x_{a,h,k} \right)\alpha_{g,a,h,k,t}\left( 1-\sigma_{a,h} \right) \right)*L_{\mathrm{RX}_{g,a,h,k,t}}$

$\frac{dL_{\mathrm{SP}_{g,a,h,k,t}}}{\mathrm{dt}} =\left( \gamma_{g,a,t}\mathrm{Se}_{I_{h,t}}{\psi_{X_{t}}\mathrm{Sp}_{R_{t}}\eta}_{S_{t}}\left( 1-\xi_{h} \right)\tau_{S_{h,k,t}}+\gamma_{g,a,t}\mathrm{Se}_{I_{h,t}}{\left( 1-\psi_{X_{h,t}} \right)\eta}_{S_{t}}\left( 1-\xi_{h} \right)\tau_{S_{h,k,t}}+\gamma_{g,a,t}\mathrm{Se}_{I_{h,t}}{\psi_{X_{t}}\left( 1-\mathrm{Sp}_{R_{t}} \right)\eta}_{R_{t}}\tau_{R_{h,k,t}} \right)*I_{\mathrm{SX}_{g,a,h,k,t}}$

+$\left( d_{N}\gamma_{g,a,t}\mathrm{Se}_{N_{h,t}}{\psi_{X_{t}}\mathrm{Sp}_{R_{t}}\eta}_{S_{t}}\left( 1-\xi_{h} \right)\tau_{S_{h,k,t}}+d_{N}\gamma_{g,a,t}\mathrm{Se}_{N_{h,t}}{\left( 1-\psi_{X_{t}} \right)\eta}_{S_{t}}\left( 1-\xi_{h} \right)\tau_{S_{h,k,t}}+d_{N}\gamma_{g,a,t}\mathrm{Se}_{N_{h,t}}{\psi_{X_{t}}\left( 1-\mathrm{Sp}_{R_{t}} \right)\eta}_{R_{t}}\tau_{R_{h,k,t}} \right)*N_{\mathrm{SX}_{g,a,h,k,t}}$

$-\left( \lambda_{S_{g,a,t}}\left( 1-x_{a,h,k} \right)\alpha_{g,a,h,k,t}+\lambda_{R_{g,a,t}}\left( 1-x_{a,h,k} \right)\alpha_{g,a,h,k,t}+\lambda_{R_{g,a,t}}\left( 1-x_{a,h,k} \right)\left( 1-\alpha_{g,a,h,k,t} \right)\iota+\nu_{g,a,h,k,t} \right)*L_{\mathrm{SP}_{g,a,h,k,t}}$

+$\left( r_{h}+ \gamma_{g,a,t}\mathrm{Se}_{I_{h,t}}{\psi_{P_{t}}\mathrm{Sp}_{R_{t}}\eta}_{S_{t}}\left( 1-\xi_{h} \right)\tau_{S_{h,k,t}}+\gamma_{g,a,t}\mathrm{Se}_{I_{h,t}}{\left( {1-\psi}_{P_{t}} \right)\eta}_{S_{t}}\left( 1-\xi_{h} \right)\tau_{S_{h,k,t}}+\gamma_{g,a,t}\mathrm{Se}_{I_{h,t}}{\psi_{P_{t}}\left( 1-\mathrm{Sp}_{R_{t}} \right)\eta}_{R_{t}}\tau_{R_{h,k,t}} \right)*I_{\mathrm{SP}_{g,a,h,k,t}}$

+$\left( r_{h}+d_{N}\gamma_{g,a,t}\mathrm{Se}_{N_{h,t}}{\psi_{P_{t}}\mathrm{Sp}_{R_{t}}\eta}_{S_{t}}\left( 1-\xi_{h} \right)\tau_{S_{h,k,t}}+d_{N}\gamma_{g,a,t}\mathrm{Se}_{N_{h,t}}{\left( 1-\psi_{P_{t}} \right)\eta}_{S_{t}}\left( 1-\xi_{h} \right)\tau_{S_{h,k,t}}+d_{N}\gamma_{g,a,t}\mathrm{Se}_{N_{h,t}}{\psi_{P_{t}}\left( 1-\mathrm{Sp}_{R_{t}} \right)\eta}_{R_{t}}\tau_{R_{h,k,t}} \right)*N_{\mathrm{SP}_{g,a,h,k,t}}$

$+\left( \lambda_{s_{g,a,t}}\left( 1-\iota\right)\left( 1-x_{a,h,k} \right)\left( 1-\alpha_{g,a,h,k,t} \right) \right)*L_{\mathrm{RX}_{g,a,h,k,t}}$

$\frac{dI_{\mathrm{SP}_{g,a,h,k,t}}}{\mathrm{dt}} =\left( \gamma_{g,a,t}\mathrm{Se}_{I_{h,t}}\psi_{X_{t}}\mathrm{Sp}_{R_{t}}\eta_{S_{t}}\left( 1-\xi_{h} \right)\left( 1-\tau_{S_{h,k,t}} \right)+\gamma_{g,a,t}\mathrm{Se}_{I_{h,t}}\left( 1-\psi_{X_{t}} \right)\eta_{S_{t}}\left( 1-\xi_{h} \right)\left( 1-\tau_{S_{h,k,t}} \right)+\gamma_{g,a,t}\mathrm{Se}_{I_{h,t}}\psi_{X_{t}}\left( 1-\mathrm{Sp}_{R_{t}} \right)\eta_{R_{t}}\left( 1-\tau_{R_{h,k,t}} \right) \right)*I_{\mathrm{SX}_{g,a,h,k,t}}$

$+\left( \lambda_{S_{g,a,t}}\left( 1-x_{a,h,k} \right)\alpha_{g,a,h,k,t}\sigma_{a,h}+\nu_{g,a,h,k,t}\sigma_{a,h} \right)*L_{\mathrm{SP}_{g,a,h,k,t}}$

$-\left( r_{h}+\gamma_{g,a,t}\mathrm{Se}_{I_{h,t}}\psi_{P_{t}}\mathrm{Sp}_{R_{t}}\eta_{S_{t}}\left( 1-\xi_{h} \right)\tau_{S_{h,k,t}}+\gamma_{g,a,t}\mathrm{Se}_{I_{h,t}}\left( {1-\psi}_{P_{t}} \right)\eta_{S_{t}}\left( 1-\xi_{h} \right)\tau_{S_{h,k,t}}+\gamma_{g,a,t}\mathrm{Se}_{I_{h,t}}\psi_{P_{t}}\left( 1-\mathrm{Sp}_{R_{t}} \right)\eta_{R_{t}}\tau_{R_{h,k,t}}+\gamma_{g,a,t}\mathrm{Se}_{I_{h,t}}\psi_{P_{t}}\mathrm{Sp}_{R_{t}}\eta_{S}\xi_{h}+\gamma_{g,a,t}\mathrm{Se}_{I_{h,t}}\left( 1-\psi_{P_{t}} \right)\eta_{S_{t}}\xi_{h} \right)*I_{\mathrm{SP}_{g,a,h,k,t}}$

$+\left( \theta_{h} \right)*N_{\mathrm{SP}_{g,a,h,k,t}}$

$+\left( \lambda_{S_{g,a,t}}\left( 1-x_{a,h,k} \right)\alpha_{g,a,h,k,t}\sigma_{a,h} \right)*L_{\mathrm{RP}_{g,a,h,k,t}}$

$\frac{dN_{\mathrm{SP}_{g,a,h,k,t}}}{\mathrm{dt}} =\left( d_{N}\gamma_{g,a,t}\mathrm{Se}_{N_{h,t}}\psi_{X_{t}}\mathrm{Sp}_{R_{t}}\eta_{S_{t}}\left( 1-\xi_{h} \right)\left( 1-\tau_{S_{h,k,t}} \right)+d_{N}\gamma_{g,a,t}\mathrm{Se}_{N_{h,t}}\left( 1-\psi_{X_{t}} \right)\eta_{S_{t}}\left( 1-\xi_{h} \right)\left( 1-\tau_{S_{h,k,t}} \right)+d_{N}\gamma_{g,a,t}\mathrm{Se}_{N_{h,t}}\psi_{X_{t}}\left( 1-\mathrm{Sp}_{R_{t}} \right)\eta_{R_{t}}\left( 1-\tau_{R_{h,k,t}} \right) \right)*N_{\mathrm{SX}_{g,a,h,k,t}}$ ^[[3]](#footnote-4)^

$+\left( \lambda_{S_{g,a,t}}\left( 1-x_{a,h,k} \right)\alpha_{g,a,h,k,t}\left( 1-\sigma_{a,h} \right)+\nu_{g,a,h,k,t}\left( 1-\sigma_{a,h} \right) \right)*L_{\mathrm{SP}_{g,a,h,k,t}}$

$-\left( r_{h}+d_{N}\gamma_{g,a,t}\mathrm{Se}_{N_{h,t}}\psi_{P_{t}}\mathrm{Sp}_{R_{t}}\eta_{S_{t}}\left( 1-\xi_{h} \right)\tau_{S_{h,k,t}}+d_{N}\gamma_{g,a,t}\mathrm{Se}_{N_{h,t}}\left( 1-\psi_{P_{t}} \right)\eta_{S_{t}}\left( 1-\xi_{h} \right)\tau_{S_{h,k,t}}+d_{N}\gamma_{g,a,t}\mathrm{Se}_{N_{h,t}}\psi_{P_{t}}\left( 1-\mathrm{Sp}_{R_{t}} \right)\eta_{R_{t}}\tau_{R_{h,k,t}}+d_{N}\gamma_{g,a,t}\mathrm{Se}_{N_{h,t}}\psi_{P_{t}}\mathrm{Sp}_{R_{t}}\eta_{S_{t}}\xi_{h}+d_{N}\gamma_{g,a,t}\mathrm{Se}_{N_{h,t}}\left( 1-\psi_{P_{t}} \right)\eta_{S_{t}}\xi_{h}+\theta_{h} \right)*N_{\mathrm{SP}_{g,a,h,k,t}}$

$+\left( \lambda_{S_{g,a,t}}\left( 1-x_{a,h,k} \right)\alpha_{g,a,h,k,t}\left( 1-\sigma_{a,h} \right) \right)*L_{\mathrm{RP}_{g,a,h,k,t}}$

$\frac{dL_{\mathrm{RX}_{g,a,h,k,t}}}{\mathrm{dt}}=\left( \lambda_{R_{g,a,t}}\left( 1-\alpha_{g,a,h,k,t} \right) \right)*S_{g,a,h,k,t}$

$+\left( \lambda_{R_{g,a,t}}\left( 1-x_{a,h,k} \right)\left( 1-\alpha_{g,a,h,k,t} \right)\iota\right)*L_{\mathrm{SX}_{g,a,h,k,t}}$

$-\left( \lambda_{S_{g,a,t}}\left( 1-x_{a,h,k} \right)\alpha_{g,a,h,k,t}+\lambda_{S_{g,a,t}}\left( 1-x_{a,h,k} \right)\left( 1-\alpha_{g,a,h,k,t} \right)\left( 1-\iota\right)+\lambda_{R_{g,a,t}}\left( 1-x_{a,h,k} \right)\alpha_{g,a,h,k,t}+\nu\right)*L_{\mathrm{RX}_{g,a,h,k,t}}$

$+\left( r_{h} \right)*I_{\mathrm{RX}_{g,a,h,k,t}}$

$+\left( r_{h} \right)*N_{\mathrm{RX}_{g,a,h,k,t}}$

$\frac{dI_{\mathrm{RX}_{g,a,h,k,t}}}{\mathrm{dt}} =\left( \lambda_{R_{g,a,t}}\alpha_{g,a,h,k,t}\sigma_{a,h} \right)*S_{g,a,h,k,t}$

$+\left( \lambda_{R_{g,a,t}}\left( 1-x_{a,h,k} \right)\alpha_{g,a,h,k,t}\sigma_{a,h} \right)*L_{\mathrm{SX}_{g,a,h,k,t}}$

$+\left( \lambda_{R_{g,a,t}}\left( 1-x_{a,h,k} \right)\alpha_{g,a,h,k,t}\sigma+\nu_{g,a,h,k,t}\sigma_{a,h} \right)*L_{\mathrm{RX}_{g,a,h,k,t}}$

$-\left( r_{h}+\gamma_{g,a,t}\mathrm{Se}_{I_{h,t}}\psi_{X_{t}}\mathrm{Se}_{R_{t}}\eta_{R_{t}}+\gamma_{g,a,t}\mathrm{Se}_{I_{h,t}}\left( 1-\psi_{X_{t}} \right)\eta_{S_{t}}+\gamma_{g,a,t}\mathrm{Se}_{I_{h,t}}\psi_{X_{t}}\left( 1-\mathrm{Se}_{R_{t}} \right)\eta_{S_{t}} \right)*I_{\mathrm{RX}_{g,a,h,k,t}}$

$+\left( \theta_{h} \right)*N_{\mathrm{RX}_{g,a,h,k,t}}$

$\frac{dN_{\mathrm{RX}_{g,a,h,k,t}}}{\mathrm{dt}} =\left( \lambda_{R_{g,a,t}}\alpha_{g,a,h,k,t}\left( 1-\sigma_{a,h} \right) \right)*S_{g,a,h,k,t}$

$+\left( \lambda_{R_{g,a,t}}\left( 1-x_{a,h,k} \right)\alpha_{g,a,h,k,t}\left( 1-\sigma_{a,h} \right) \right)*L_{\mathrm{SX}_{g,a,h,k,t}}$

$+\left( \lambda_{R_{g,a,t}}\left( 1-x_{a,h,k} \right)\alpha_{g,a,h,k,t}\left( 1-\sigma_{a,h} \right)+\nu_{g,a,h,k,t}\left( 1-\sigma_{a,h} \right) \right)*L_{\mathrm{RX}_{g,a,h,k,t}}$

$-\left( r_{h}+d_{N}\gamma_{g,a,t}\mathrm{Se}_{N_{h,t}}\psi_{X_{t}}\mathrm{Se}_{R_{t}}\eta_{R_{t}}+d_{N}\gamma_{g,a,t}\mathrm{Se}_{N_{h,t}}\left( 1-\psi_{X_{t}} \right)\eta_{S_{t}}+d_{N}\gamma_{g,a,t}\mathrm{Se}_{N_{h,t}}\psi_{X_{t}}\left( 1-\mathrm{Se}_{R_{t}} \right)\eta_{S_{t}}+\theta_{h} \right)*N_{\mathrm{RX}_{g,a,h,k,t}}$ ^[[4]](#footnote-5)^

$\frac{dL_{\mathrm{RP}_{g,a,h,k,t}}}{\mathrm{dt}} =\left( \lambda_{R_{g,a,t}}\left( 1-x_{a,h,k} \right)\left( 1-\alpha_{g,a,h,k,t} \right)\iota\right)*L_{\mathrm{SP}_{g,a,h,k,t}}$

$+\left( \gamma_{g,a,t}\mathrm{Se}_{I_{h,t}}{\psi_{X_{t}}\mathrm{Se}_{R_{t}}\eta}_{R_{t}}\tau_{R_{h,k,t}}+\gamma_{g,a,t}\mathrm{Se}_{I_{h,t}}{\left( 1-\psi_{X_{t}} \right)\eta}_{S_{t}}\tau_{S_{h,k,t}}\mathrm{RR}_{X}+\gamma_{g,a,t}\mathrm{Se}_{I_{h,t}}{\psi_{X_{t}}\left( 1-\mathrm{Se}_{R_{t}} \right)\eta}_{S_{t}}\tau_{S_{h,k,t}}\mathrm{RR}_{X} \right)*I_{\mathrm{RX}_{g,a,h,k,t}}$

+$\left( d_{N}\gamma_{g,a,t}\mathrm{Se}_{N_{h,t}}{\psi_{X_{t}}\mathrm{Se}_{R_{t}}\eta}_{R_{t}}\tau_{R_{h,k,t}}+d_{N}\gamma_{g,a,t}\mathrm{Se}_{N_{h,t}}{\left( 1-\psi_{X_{t}} \right)\eta}_{S_{t}}\tau_{S_{h,k,t}}\mathrm{RR}_{X}+d_{N}\gamma_{g,a,t}\mathrm{Se}_{N_{h,t}}{\psi_{X_{t}}\left( 1-\mathrm{Se}_{R_{t}} \right)\eta}_{S_{t}}\tau_{S_{h,k,t}}\mathrm{RR}_{X} \right)*N_{\mathrm{RX}_{g,a,h,k,t}}$

$-\left( \lambda_{S_{g,a,t}}\left( 1-x_{a,h,k} \right)\alpha_{g,a,h,k,t}+\lambda_{S_{g,a,t}}\left( 1-x_{a,h,k} \right)\left( 1-\alpha_{g,a,h,k,t} \right)\left( 1-\iota\right)+\lambda_{R_{g,a,t}}\left( 1-x_{a,h,k} \right)\alpha_{g,a,h,k,t}+\nu_{g,a,h,k,t} \right)*L_{\mathrm{RP}_{g,a,h,k,t}}$

$+\left( r_{h}+ \gamma_{g,a,t}\mathrm{Se}_{I_{h,t}}{\psi_{P_{t}}\mathrm{Se}_{R_{t}}\eta}_{R_{t}}\tau_{R_{h,k,t}}+\gamma_{g,a,t}\mathrm{Se}_{I_{h,t}}{\left( 1-\psi_{P_{t}} \right)\eta}_{S_{t}}\tau_{S_{h,k,t}}\mathrm{RR}_{P}+\gamma_{g,a,t}\mathrm{Se}_{I_{h,t}}{\psi_{P_{t}}\left( 1-\mathrm{Se}_{R_{t}} \right)\eta}_{S_{t}}\tau_{S_{h,k,t}}\mathrm{RR}_{P} \right)*I_{\mathrm{RP}_{g,a,h,k,t}}$

$+\left( r_{h}+ d_{N}\gamma_{g,a,t}\mathrm{Se}_{N_{h,t}}{\psi_{P_{t}}\mathrm{Se}_{R_{t}}\eta}_{R_{t}}\tau_{R_{h,k,t}}+d_{N}\gamma_{g,a,t}\mathrm{Se}_{N_{h,t}}{\left( 1-\psi_{P_{t}} \right)\eta}_{S_{t}}\tau_{S_{h,k,t}}\mathrm{RR}_{P}+d_{N}\gamma_{g,a,t}\mathrm{Se}_{N_{h,t}}{\psi_{P_{t}}\left( 1-\mathrm{Se}_{R_{t}} \right)\eta}_{S_{t}}\tau_{S_{h,k,t}}\mathrm{RR}_{P} \right)*N_{\mathrm{RP}_{g,a,h,k,t}}$

$\frac{dI_{{RP}_{g,a,h,k,t}}}{\mathrm{dt}} =\left( \gamma_{g,a,t}\mathrm{Se}_{I_{h,t}}{\psi_{X_{t}}\mathrm{Sp}_{R_{t}}\eta}_{S_{t}}\xi_{h}+\gamma_{g,a,t}\mathrm{Se}_{I_{h,t}}{\left( 1-\psi_{X_{t}} \right)\eta}_{S_{t}}\xi_{h} \right) *I_{\mathrm{SX}_{g,a,h,k,t}}$

$+\left( \lambda_{R_{g,a,t}}\left( 1-x_{a,h,k} \right)\alpha_{g,a,h,k,t}\sigma_{a,h} \right)*L_{\mathrm{SP}_{g,a,h,k,t}}$

$$+\left( \gamma_{g,a,t}\mathrm{Se}_{I_{h,t}}{\psi_{P_{t}}\mathrm{Sp}_{R_{t}}\eta}_{S_{t}}\xi_{h}+\gamma_{g,a,t}\mathrm{Se}_{I_{h,t}}{\left( 1-\psi_{P_{t}} \right)\eta}_{S_{t}}\xi_{h} \right)*I_{\mathrm{SP}_{g,a,h,k,t}}$$

$+\left( \gamma_{g,a,t}\mathrm{Se}_{I_{h,t}}\psi_{X_{t}}\mathrm{Se}_{R_{t}}\eta_{R_{t}}\left( 1-\tau_{R_{h,k,t}} \right)+\gamma_{g,a,t}\mathrm{Se}_{I_{h,t}}\left( 1-\psi_{X_{t}} \right)\eta_{S_{t}}\left( 1-\tau_{S_{h,k,t}}\mathrm{RR}_{X} \right)+\gamma_{g,a,t}\mathrm{Se}_{I_{h,t}}\psi_{X_{t}}{\left( 1-\mathrm{Se}_{R_{t}} \right)\eta}_{S_{t}}\left( 1-\tau_{S_{h,k,t}}\mathrm{RR}_{X} \right) \right)*I_{\mathrm{RX}_{g,a,h,k,t}}$

$+\left( \lambda_{R_{g,a,t}}\left( 1-x_{a,h,k} \right)\alpha_{g,a,h,k,t}\sigma_{a,h}+\nu_{g,a,h,k,t}\sigma_{a,h} \right)*L_{\mathrm{RP}_{g,a,h,k,t}}$

$-\left( r_{h}+\gamma_{g,a,t}\mathrm{Se}_{I_{h,t}}\psi_{P_{t}}\mathrm{Se}_{R_{t}}\eta_{R_{t}}\tau_{R_{h,k,t}}+\gamma_{g,a,t}\mathrm{Se}_{I_{h,t}}\left( 1-\psi_{P_{t}} \right)\eta_{S_{t}}\tau_{S_{h,k,t}}\mathrm{RR}_{P}+\gamma_{g,a,t}\mathrm{Se}_{I_{h,t}}\psi_{P_{t}}\left( 1-\mathrm{Se}_{R_{t}} \right)\eta_{S_{t}}\tau_{S_{h,k,t}}\mathrm{RR}_{P} \right)*I_{\mathrm{RP}_{g,a,h,k,t}}$

$+\left( \theta_{h} \right)*N_{\mathrm{RP}_{g,a,h,k,t}}$

$\frac{dN_{\mathrm{RP}_{g,a,h,k,t}}}{\mathrm{dt}} =\left( d_{N}\gamma_{g,a,t}\mathrm{Se}_{N_{h,t}}\psi_{X_{t}}{\mathrm{Sp}_{R_{t}}\eta}_{S_{t}}\xi_{h}+d_{N}\gamma_{g,a,t}\mathrm{Se}_{N_{h,t}}\left( 1-\psi_{X_{t}} \right)\eta_{S_{t}}\xi_{h} \right)*N_{\mathrm{SX}_{g,a,h,k,t}}$

$+\left( \lambda_{R_{g,a,t}}\left( 1-x_{a,h,k} \right)\alpha_{g,a,h,k,t}\left( 1-\sigma_{a,h} \right) \right)*L_{\mathrm{SP}_{g,a,h,k,t}}$

$+\left( d_{N}\gamma_{g,a,t}\mathrm{Se}_{N_{h,t}}\psi_{P_{t}}{\mathrm{Sp}_{R_{t}}\eta}_{S_{t}}\xi_{h}+d_{N}\gamma_{g,a,t}\mathrm{Se}_{N_{h,t}}\left( 1-\psi_{P_{t}} \right)\eta_{S_{t}}\xi_{h} \right)*N_{\mathrm{SP}_{g,a,h,k,t}}$

$+\left( d_{N}\gamma_{g,a,t}\mathrm{Se}_{N_{h,t}}\psi_{X_{t}}\mathrm{Se}_{R_{t}}\eta_{R_{t}}\left( 1-\tau_{R_{h,k,t}} \right)+d_{N}\gamma_{g,a,t}\mathrm{Se}_{N_{h,t}}\left( 1-\psi_{X_{t}} \right)\eta_{S_{t}}\left( 1-\tau_{S_{h,k,t}}\mathrm{RR}_{X} \right)+d_{N}\gamma_{g,a,t}\mathrm{Se}_{N_{h,t}}\psi_{X_{t}}\left( 1-\mathrm{Se}_{R_{t}} \right)\eta_{S_{t}}\left( 1-\tau_{S_{h,k,t}}\mathrm{RR}_{X} \right) \right)*N_{\mathrm{RX}_{g,a,h,k,t}}$

$+\left( \lambda_{R_{g,a,t}}\left( 1-x_{a,h,k} \right)\alpha_{g,a,h,k,t}\left( 1-\sigma_{a,h} \right)+\nu_{g,a,h,k,t}\left( 1-\sigma_{a,h} \right) \right)*L_{\mathrm{RP}_{g,a,h,k,t}}$

$-\left( r_{h}+d_{N}\gamma_{g,a,t}\mathrm{Se}_{N_{h,t}}{\psi_{X_{t}}\mathrm{Se}_{R_{t}}\eta}_{R_{t}}\tau_{R_{h,k,t}}+d_{N}\gamma_{g,a,t}\mathrm{Se}_{N_{h,t}}{\left( 1-\psi_{P_{t}} \right)\eta}_{S_{t}}\tau_{S_{h,k,t}}\mathrm{RR}_{P}+d_{N}\gamma_{g,a,t}\mathrm{Se}_{N_{h,t}}{\psi_{P_{t}}\left( 1-\mathrm{Se}_{R_{t}} \right)\eta}_{S_{t}}\tau_{S_{h,k,t}}\mathrm{RR}_{P}+\theta_{h} \right)*N_{\mathrm{RP}_{g,a,h,k,t}}$ ^[[5]](#footnote-6)^

3 Model parameters

Parameter structures and adjustments across model strata, as well as prior ranges and data sources used for model calibration, are discussed below. Prior ranges for all parameters are summarised (and compared with posterior medians and ranges) in Section 6.1.

3.1 Force of infection

The force of infection for drug susceptible TB ($\lambda_{S_{g,a,h,t}})$ and for MDR TB ($\lambda_{R_{g,a,h,t}}$) are time-dependent parameters specific to sex, age, and HIV status. Terms for annual risk of infection for both drug susceptible TB and MDR TB acknowledge heterogeneous patterns of social contacts between men, women, and children and preferential mixing among HIV-positive individuals, as well as time-dependent sex-specific relative risks of infection attributable to tobacco smoking and constant sex-specific relative risk of infection attributable to biological or other factors.

The force of infection for drug susceptible TB (indicated by subscript *s*) is as follows:

$$\lambda_{S_{g,a,h,t}}=\left( {c_{M}}_{g,a}\frac{I_{S_{M,t}}+cN_{S_{M,t}}}{T_{t}}+{c_{F}}_{g,a}\frac{I_{S_{F,t}}+cN_{S_{F,t}}}{T_{t}}+{c_{C}}_{g,a}\frac{I_{S_{C,t}}+cN_{S_{C,t}}}{T_{t}} \right)\times$$

$$(z)\left( \mathrm{cscal} \right)\left( {amp}_{h} \right)\left( \mathrm{RR}_{s{m\_inf}_{g,a,t}}\mathrm{RR}_{\mathrm{sex}_{g,a}} \right)$$

for sex *g*, age *a*, HIV status *h*, and time-step *t*, where c_M*_g,a_* is the average number of contacts with men, $I_{S_{M,t}}$ is the number of men with drug susceptible smear-positive TB, *c* is the relative infectiousness of smear-negative TB compared to smear-positive TB, $N_{S_{M,t}}$ is the number of men with drug susceptible smear-negative TB, T is the total population, c_F*_g,a_* is the average number of contacts with women, $I_{S_{F,t}}$ is the number of women with drug susceptible smear-positive TB, $N_{S_{F,t}}$ is the number of women with drug susceptible smear-negative TB, c_C*_g,a_* is the average number of contacts with children, $I_{S_{C,t}}$ is the number of children with drug susceptible smear-positive TB, $N_{S_{C,t}}$ is the number of children with drug susceptible smear-negative TB, *z* is the probability of *Mtb* transmission per respiratory contact between an infectious and an uninfected individual, *cscal* is a scaling factor to adjust the total number of contacts, ${amp}_{h}$ is a scaling factor to acknowledge more frequent preferential mixing among HIV-positive individuals, ${RR}_{s{m\_inf}_{g,a,t}}$ is the relative risk of infection attributable to tobacco smoking, and ${RR}_{s{ex}_{g,a}}$ is the relative risk of infection attributable to additional sex- or gender-based risks. Prior ranges and data sources for parameters are described in Table 35 and described below.

The annual risk of infection for MDR TB (indicated by subscript *r*) is as follows:

$$\lambda_{R_{g,a,h,t}}=\left( {c_{M}}_{g,a}\frac{I_{R_{M,t}}+cN_{R_{M,t}}}{T_{t}}+{c_{F}}_{g,a}\frac{I_{R_{F,t}}+cN_{R_{F,t}}}{T_{t}}+{c_{C}}_{g,a}\frac{I_{R_{C,t}}+cN_{R_{C,t}}}{T_{t}} \right)\times$$

$$(z)(\varphi)\left( \mathrm{cscal} \right)\left( {amp}_{h} \right)(\mathrm{RR}_{s{m\_inf}_{g,a,t}}\mathrm{RR}_{\mathrm{sex}_{g,a}})$$

for sex *g*, age *a*, HIV status *h*, and time-step *t*, where $I_{R_{M,t}}$ is the number of men with drug susceptible smear-positive TB, $N_{R_{M,t}}$ is the number of men with drug susceptible smear-negative TB, $I_{R_{F,t}}$ is the number of women with drug susceptible smear-positive TB, $N_{R_{F,t}}$ is the number of women with drug susceptible smear-negative TB, $I_{R_{C,t}}$ is the number of children with drug susceptible smear-positive TB, $N_{R_{C,t}}$ is the number of children with drug susceptible smear-negative TB, *φ* is the relative fitness of MDR strains, and remaining terms are defined as above. Prior ranges and data sources for parameters are described in Table 5.

Table 5: Prior ranges and data sources for parameters related to annual risk of infection

| Parameter | Description | Prior range  or set value | Reference |
| --- | --- | --- | --- |
| *c* | Relative infectiousness of smear-negative TB compared to smear-positive TB | 0.10-0.37 | [1, 5-7] |
| *z* | Probability of transmission per respiratory contact between infectious and uninfected individuals | 0.1 | [8-10] |
| *cscal* | Scaling factor to adjust the total number of contacts | 10-25 | Assumption |
| φ | Relative fitness of MDR strains compared to drug-susceptible strains | 0.58-0.85 | [1, 7, 11] |

Adjustments for heterogeneous mixing

The force of infection acknowledges heterogeneous mixing by sex and age by incorporating the average number of contacts between men (male, age ≥ 15 years), women (female, age ≥ 15 years), and children (both sexes, age < 15 years). Estimates for average number of contacts are based on a 2007 social contact survey in northern Viet Nam [12]. The contact matrix (Table 6) reports the average number of close contacts within a 24-hour period between each participant group and each contact group, with smoothing per Baguelin et al. to ensure symmetry [13].

Table 6: Contact matrix

| Participants | Average number of close contacts | | |
| --- | --- | --- | --- |
|  | Men | Women | Children |
| Men | 3.64 | 3.12 | 1.36 |
| Women | 2.38 | 4.21 | 1.42 |
| Children | 1.44 | 1.97 | 3.46 |

Adjustments for tobacco smoking

The relative risk of *Mtb* infection attributable to tobacco smoking (${RR}_{sm_{g,a,t}}$) is defined as follows:

$$\mathrm{RR}_{s{m\_inf}_{g,a,t}}= p_{{sm}_{g,a,t}}*{RR}_{sm\_inf}+\left( 1-p_{{sm}_{g,a,t}} \right)$$

for sex *g*, age *a*, and time-step *t*, where $p_{{sm}_{g,a,t}}$ is the proportion of current smokers and ${RR}_{sm\_inf}$ refers to the relative risk of *Mtb* infection attributable to tobacco smoking.

The sex-specific proportion of current smokers ($p_{{sm}_{g,a,t}}$ ) is based on IHME estimates for the number of daily smokers (Figure 5) [14] divided by UN World Population Prospects population estimates [15]. Estimates are specific to the population age 15 years and older; the proportion of current smokers in ages 0-14 years is assumed 0. Trends in the proportion of current smokers over the period 1980 through 1984 were extended to generate estimates for 1970 through 1979. The proportions of male and female smokers were assumed constant from 2015 through 2035. IHME estimates were used because estimates are available annually from 1980 through 2015 to allow more accurate estimates of historical trends, compared to WHO estimates and projections, which were only available at five-year intervals from 2000 through 2025 [16].


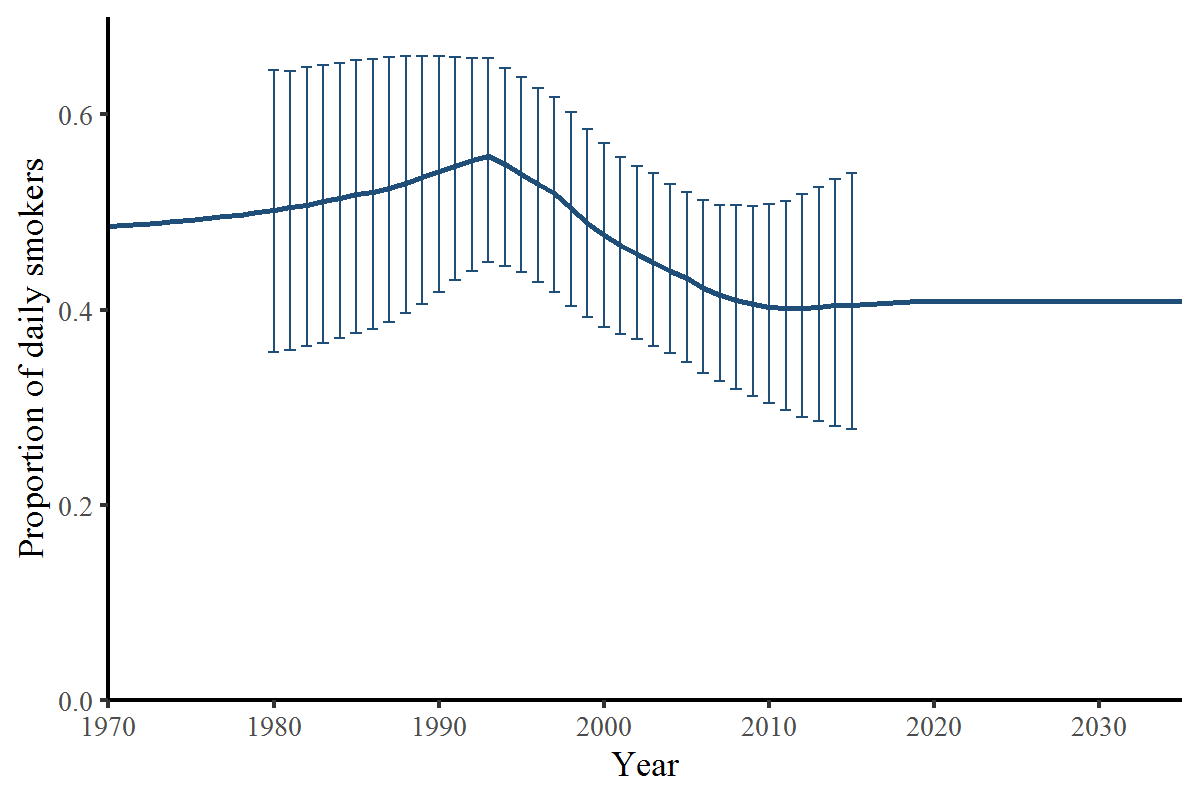

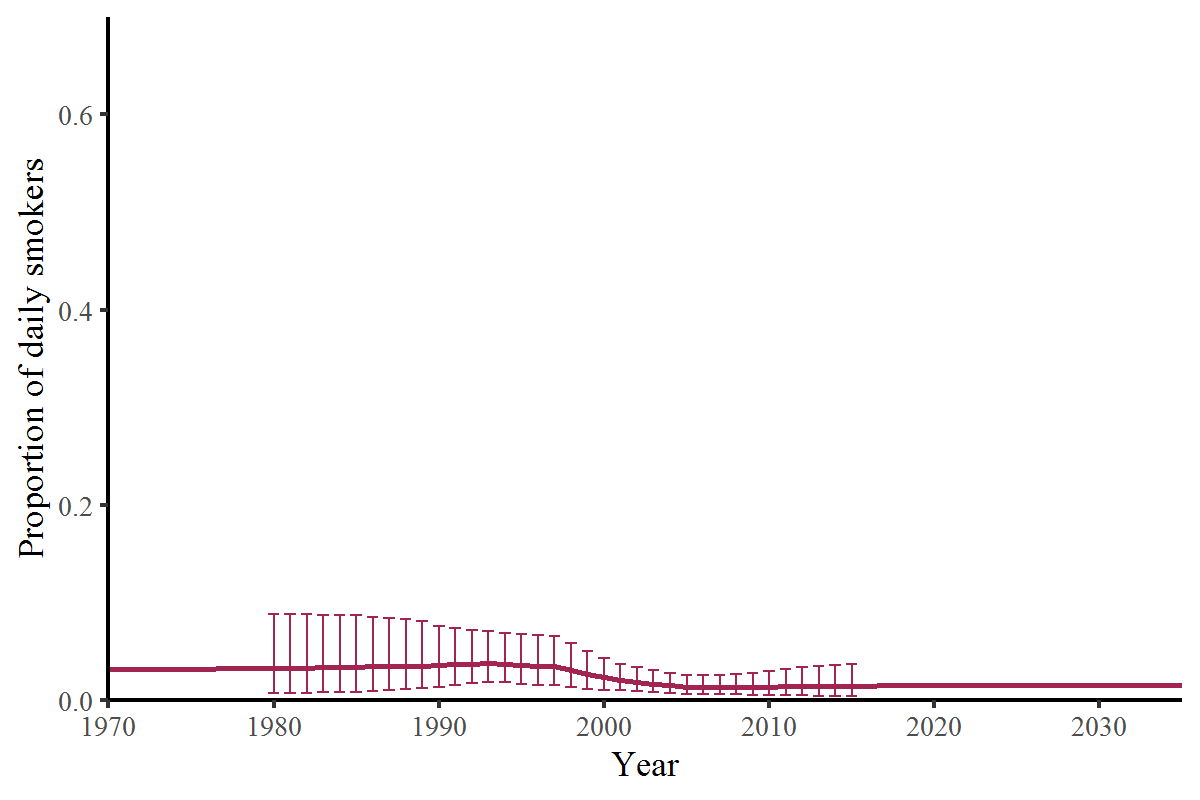


Figure 5: Model priors (line) and IHME estimates (error bars) for proportion of current tobacco smokers for men (on left in blue) and women (on right in red)

The relative risk of *Mtb* infection among current tobacco smokers (${RR}_{sm\_inf})$is based on estimates from systematic reviews and meta-analyses (Table 7).

Table 7: Prior range for relative risk of *Mtb* infection among current tobacco smokers relative to non-smokers

| Parameter | Description | Prior range  or set value | Reference |
| --- | --- | --- | --- |
| ${RR}_{sm\_inf}$ | Relative risk of *Mtb* infection among current smokers | 1.46-2.30 | [17-19] |

Adjustments for additional sex- or gender-based risks

Relative risk of infection attributable to additional sex- or gender-based risks ($\mathrm{RR}_{\mathrm{sex}_{g,a}})$ reflects further unspecified risks that may contribute to men’s increased risk of *Mtb* infection relative to women and children (Table 8). Such factors could include anatomical and immunological factors [20, 21], and their relative risks are considered constant over time. The relative risk of infection attributable to additional sex- or gender-based risks is assumed 1 for women (females, age ≥15 years) and children (both sexes, age 0-14 years).

Table 8: Prior ranges and data sources for relative risks of *Mtb* infection attributable to additional sex- or gender-based risks

| Parameter | Description | Prior range  or set value | Reference |
| --- | --- | --- | --- |
| $\mathrm{RR}_{\mathrm{sex}_{g=M,a\geq15}}$ | Relative risk of infection attributable to additional sex- or gender-based risks in men | 1-2 | Assumption |
| $\mathrm{RR}_{\mathrm{sex}_{g=F,a\geq15}}$ | Relative risk of infection attributable to additional sex- or gender-based risks in women | 1 | Assumption |
| $\mathrm{RR}_{\mathrm{sex}_{g,a=0-14}}$ | Relative risk of infection attributable to additional sex- or gender-based risks in children | 1 | Assumption |

Amplified mixing among HIV-positive individuals

Contacts between HIV-positive individuals, regardless of TB, CD4, or ART category, are amplified by a scaling factor (${amp}_{h=HIV+}$) to acknowledge more frequent preferential mixing among HIV-positive individuals within the context of a concentrated HIV epidemic (Table 9). We do not assume any further amplification of mixing among HIV-negative individuals.

Table 9: Prior range for scaling factor to acknowledge more frequent preferential mixing among HIV-positive individuals

| Parameter | Description | Prior range  or set value | Reference |
| --- | --- | --- | --- |
| $\mathrm{amp}_{h=HIV+}$ | Scaling factor to amplify contacts between HIV-positive individuals | 1-5 | Assumption |
| $\mathrm{amp}_{h=HIV-}$ | Scaling factor to amplify contacts between HIV-negative individuals | 1 | Assumption |

3.2 Progression to active disease

3.2.1 Proportion protection due to previous infection against progression to active disease following reinfection

The proportion protection due to previous infection against progression to active disease following reinfection ($x_{a,h,k})$ is a constant parameter specific to age, HIV status, and ART duration.

Prior ranges and data sources for proportion protection against progression to active disease following reinfection due to previous infection in HIV-negative individuals are shown in Table 10.

Table 10: Prior ranges and data sources for proportion protection due to previous infection against progression to active disease following reinfection in HIV-negative individuals

| Parameter | Description | Prior range  or set value | Reference |
| --- | --- | --- | --- |
| $x_{a\geq15}$ | Proportion protection due to previous infection against progression to active disease following reinfection for age ≥ 15 years | 0.37-0.90 | [1, 6, 7, 22, 23] |
| $x_{a=0-14}$ | Proportion protection due to previous infection against progression to active disease following reinfection for ages 0-14 years | 0.37-0.90 | [1, 6, 7, 22, 23] |

HIV-positive strata

Following the structure used by Houben et al. [1], proportion protection due to previous infection against progression to active disease following reinfection is adjusted for HIV status based on CD4 count using two relative risks: RR1 as the initial change in risk due to HIV infection, and RR2 as the change in risk attributable to each 100 cell/μL change in CD4 count (Table 11). The CD4-dependent value for the proportion protection due to previous infection against progression to active disease following reinfection is defined as follows:

$$x_{i}=x*{RR}_{1}*{RR}_{2}^{\frac{(500-{mid}_{i})}{100}}$$

where *i* indicates the CD4 category, *RR_1_* and *RR_2_* are parameter dependent relative risks, and *mid_i_* is the midpoint of CD4 category *i*. The midpoint of the CD4 category for counts greater than 500 cells/μL is defined as 500 such that $x_{>500}=x*{RR}_{1}$.

Table 11: Prior ranges and data sources for risk ratios for protection due to previous infection against progression to active disease following reinfection in HIV-positive individuals

| Parameter | Description | Prior range  or set value | Reference |
| --- | --- | --- | --- |
| *RR1_x_* | Risk ratio for protection due to previous infection against progression to active disease following reinfection in HIV-positive individuals with CD4 > 500 cells/μL | 0.60-1.00 | [1, 6, 7, 23] |
| *RR2_x_* | Risk ratio for protection due to previous infection against progression to active disease following reinfection in HIV-positive individuals for each 100 cell/μL change in CD4 | 0.50-1.00 | [1, 6, 7, 23] |

ART strata

ART reduces the difference between the CD4-dependent value and the value for an HIV-negative individual, with increasing effect for increasing duration of ART. ART increases the proportion protection due to previous infection against progression to active disease following reinfection as follows:

$$x_{j,l}^{A}=min(1-1-\left( x_{j}^{H} \right)*\left( 1-{ART}_{l} \right),x)$$

where *A* refers to HIV-positive individuals on ART, *H* refers to HIV-positive individuals not on ART, *j* refers to CD4 category, and *l* refers to ART duration such that *ART_l_* is the protective effect of ART by ART duration (Table 12). The *min* function ensures that ART does not raise protection above that experienced by HIV-negative individuals.

Table 12: Prior ranges and data sources for protective effect of ART on TB disease progression by ART duration

| Parameter | Description | Prior range  or set value | Reference |
| --- | --- | --- | --- |
| ${ART}_{{TB}_{l<6}}$ | Protective effect of ART on TB disease progression for duration < 6 months | 0.16-0.27 | [1, 24] |
| ${ART}_{{TB}_{l=6-12}}$ | Protective effect of ART on TB disease progression for duration 6-12 months | 0.43-0.73 | [1, 24] |
| ${ART}_{{TB}_{l\geq12}}$ | Protective effect of ART on TB disease progression for duration ≥ 12 months | 0.54-0.92* | [1, 24] |

*${ART}_{l\geq12}$ must be greater than ${ART}_{l=6-12}$

3.2.2 Proportion of new infections developing primary disease

The proportion of new infections developing primary disease ($\alpha_{g,a,h,k,t})$ is a time-dependent parameter specific to sex, age, HIV status, and ART duration.

The proportion of new infections developing primary disease in HIV-negative individuals is defined as follows:

$$\alpha_{g,a}=\alpha_{a}\mathrm{RR}_{s{m\_prog}_{g,a,t}}\mathrm{RR}_{\mathrm{alc}_{g,a,t}}{RR}_{BCG}$$

for sex *g*, age *a*, and time-step *t*, where $\alpha_{a}$ is the base proportion of new infections developing primary disease (Table 13), ${RR}_{s{m\_prog}_{g,a,t}}$ is the relative risk of progression from *Mtb* infection to disease attributable to tobacco smoking, ${RR}_{{alc}_{g,a,t}}$ is the relative risk of progression from *Mtb* infection to disease attributable to alcohol consumption, and ${RR}_{BCG}$ is the relative risk of progression from *Mtb* infection to disease attributable to BCG vaccination.

Table 13: Prior ranges and data sources for base proportion of new infections developing primary disease in HIV-negative individuals

| Parameter | Description | Prior range  or set value | Reference |
| --- | --- | --- | --- |
| $\alpha_{a\geq15}$ | Base proportion of new infections developing primary disease in ages ≥ 15 years | 0.08-0.15* | [1, 6, 7, 25] |
| $\alpha_{a=10-14}$ | Base proportion of new infections developing primary disease in ages 10-14 years | 0.032-0.104† | [1, 7, 25, 26] |
| $\alpha_{a=5-9}$ | Base proportion of new infections developing primary disease in ages 5-9 years | 0.080-0.260‡ | [1, 7, 25, 26] |
| $\alpha_{a=0-4}$ | Base proportion of new infections developing primary disease in ages 0-4 years | 0.151-0.495 | [1, 7, 25, 26] |

* $\alpha_{a\geq15}$ must be greater than $\alpha_{a=10-14}$

† $\alpha_{a=10-14}$ must be greater than $\alpha_{a=5-9}$

‡ $\alpha_{a=5-9}$ must be greater than $\alpha_{a=0-4}$

*Adjustments for tobacco smoking*

The relative risk of progression from *Mtb* infection to disease attributable to tobacco smoking (${RR}_{s{m\_prog}_{g,a,t}}$) is defined as follows:

$$\mathrm{RR}_{s{m\_prog}_{g,a,t}}= p_{{sm}_{g,a,t}}*{RR}_{sm\_prog}+\left( 1-p_{{sm}_{g,a,t}} \right)$$

for sex *g*, age *a*, and time-step *t*, where $p_{{sm}_{g,a,t}}$ is the proportion of current smokers (see Section 3.1.1) and ${RR}_{sm\_prog}$ refers to the relative risk of progression from *Mtb* infection to disease attributable to tobacco smoking (Table 14).

Table 14: Prior ranges and data sources for relative risks of progression from *Mtb* infection to disease among current tobacco smokers relative to non-smokers

| Parameter | Description | Prior range  or set value | Reference |
| --- | --- | --- | --- |
| ${RR}_{sm\_prog}$ | Relative risk of progression from *Mtb* infection to disease among current smokers | 1.46-2.30 | [17-19] |

*Adjustments for alcohol consumption*

The relative risk of progression from *Mtb* infection to disease attributable to alcohol consumption (${RR}_{{alc}_{g,a,t}}$) is defined as follows:

$$\mathrm{RR}_{{alc}_{g,a,t}}= p_{{alc}_{g,a,t}}*{cons}_{{alc}_{g,a,t}}{RR}_{alc}+\left( 1-p_{{alc}_{g,a,t}} \right)$$

for sex *g*, age *a*, and time-step *t*, where $p_{{alc}_{g,a,t}}$ is the proportion of current alcohol drinkers, ${cons}_{{alc}_{g,a,t}}$ is the standard drinks (in grams) consumed daily by current alcohol drinkers, and ${RR}_{alc}$ refers to the relative risk of progression from *Mtb* infection to disease attributable to alcohol consumption.

The sex-specific proportion of current alcohol drinkers ($p_{{alc}_{g,a,t}}$ ) is based on IHME estimates (Figure 6) [27]. Although the model is designed to use alcohol consumption estimates for ages 15 years and older, only age-standardised prevalence estimates were available; the proportion of current alcohol drinkers in ages 0-14 years is assumed 0. Estimates are available for five-year intervals from 1990 through 2016; estimates for intermediate time points assume linear trends within each five-year interval. Trends in the proportion of current alcohol drinkers over the period 1980 through 1984 were extended to generate estimates for 1970 through 1979. Proportions of male and female drinkers were assumed constant from 2015 through 2035.


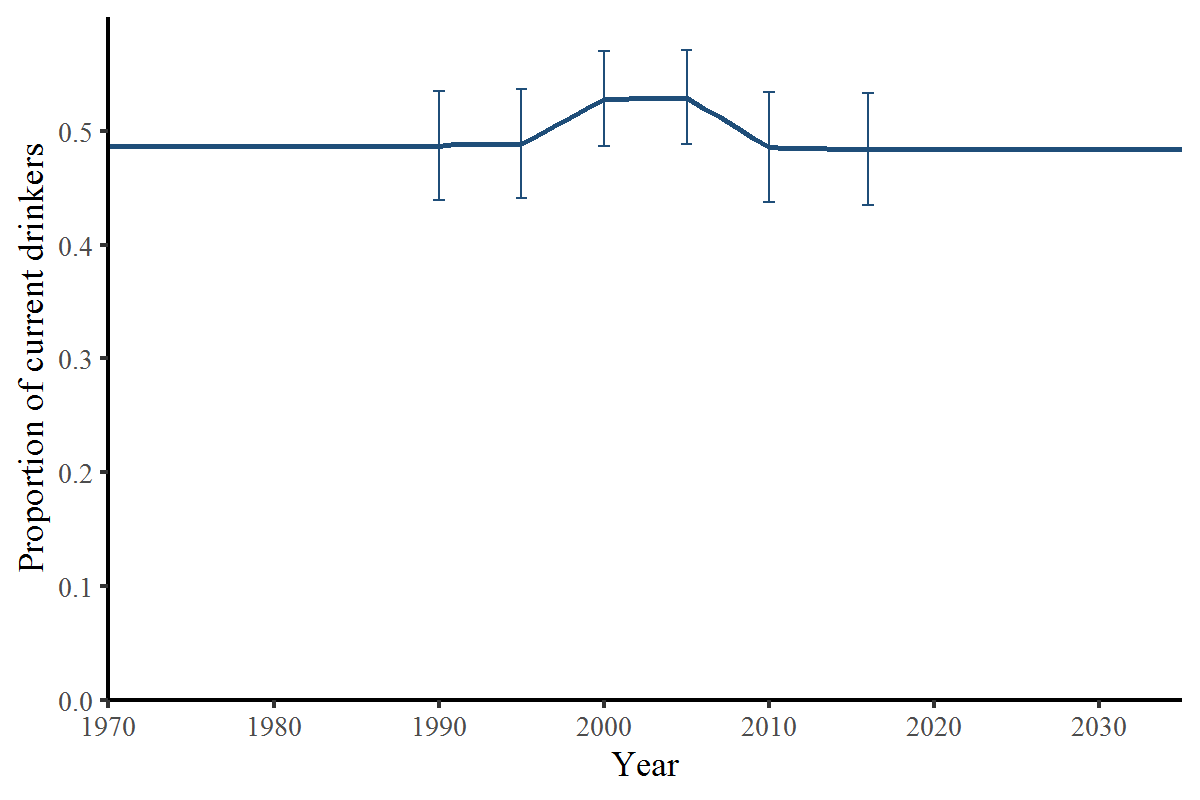

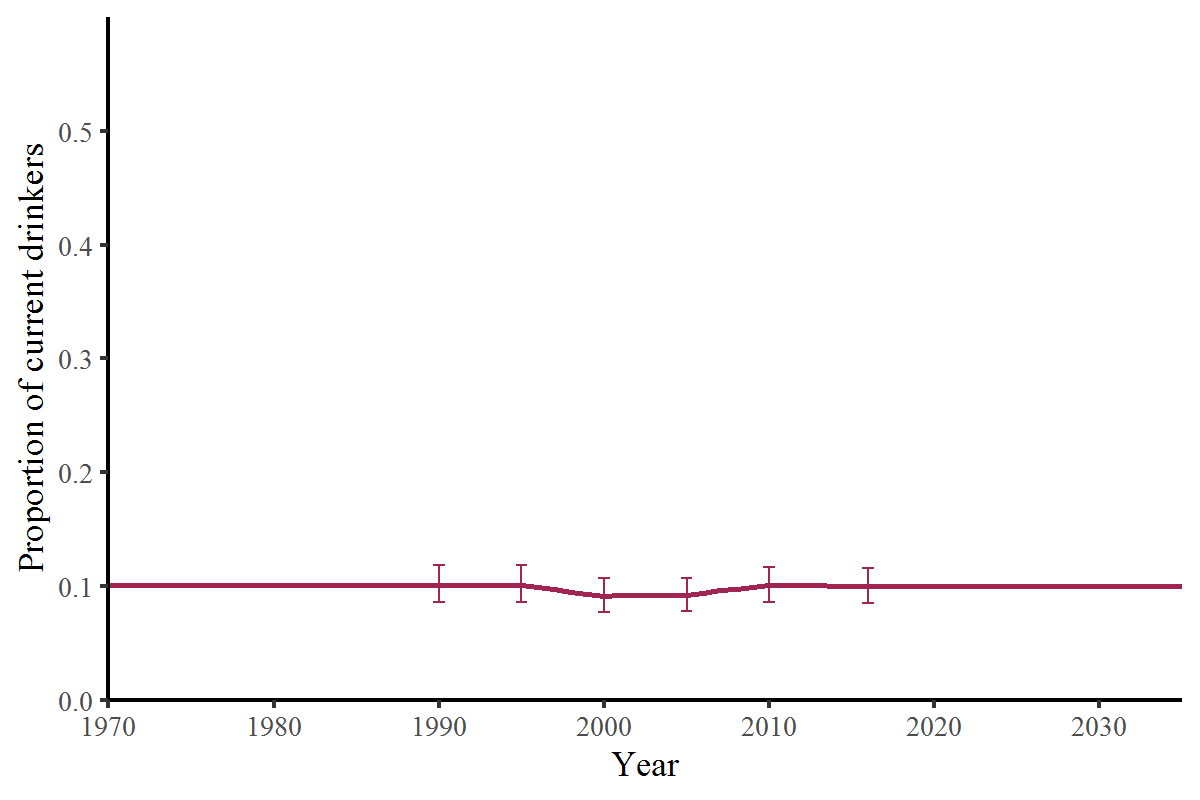


Figure 6: Model priors (line) and IHME estimates (error bars) for proportion of current alcohol drinkers for men (on left in blue) and women (on right in red)

Estimates for standard drinks (in grams) consumed daily by current alcohol drinkers (${cons}_{{alc}_{g,a,t}}$) are based on IHME estimates (Figure 7) [27]. Although the model is designed to use alcohol consumption estimates for ages 15 years and older, only age-standardised prevalence estimates were available; the proportion of current alcohol drinkers in ages 0-14 years is assumed 0. Estimates are available for five-year intervals from 1990 through 2016. We assume linear trends between five-year estimates during this time period. We assume continued increase in alcohol consumption over the period 2016-2020 proportional to the declining rate of increase observed between 2005-2010 and 2010-2015. Alcohol consumption is assumed constant from 2020 through 2035. IHME estimates were used because estimates are available at five-year intervals from 1990 through 2016 to allow more accurate estimates of historical trends, compared to World Bank estimates and projections, which were only available for 2010 and 2016 [28, 29].


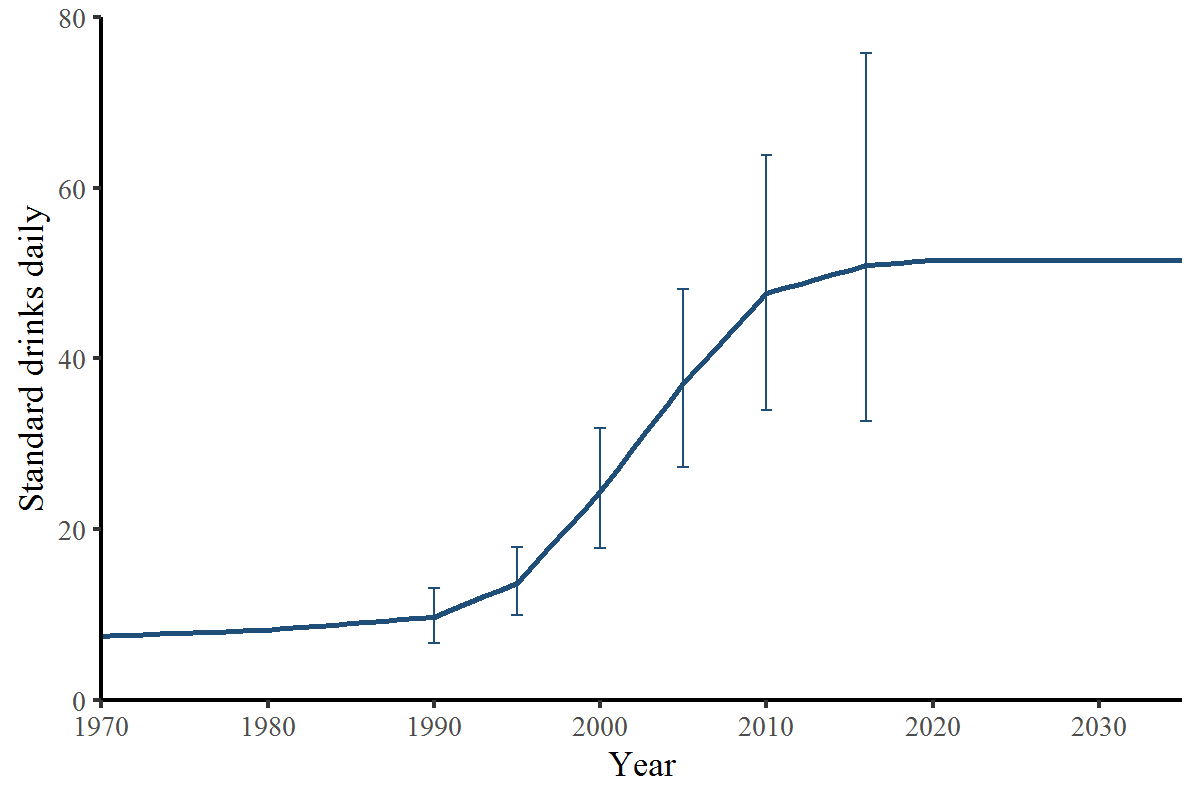

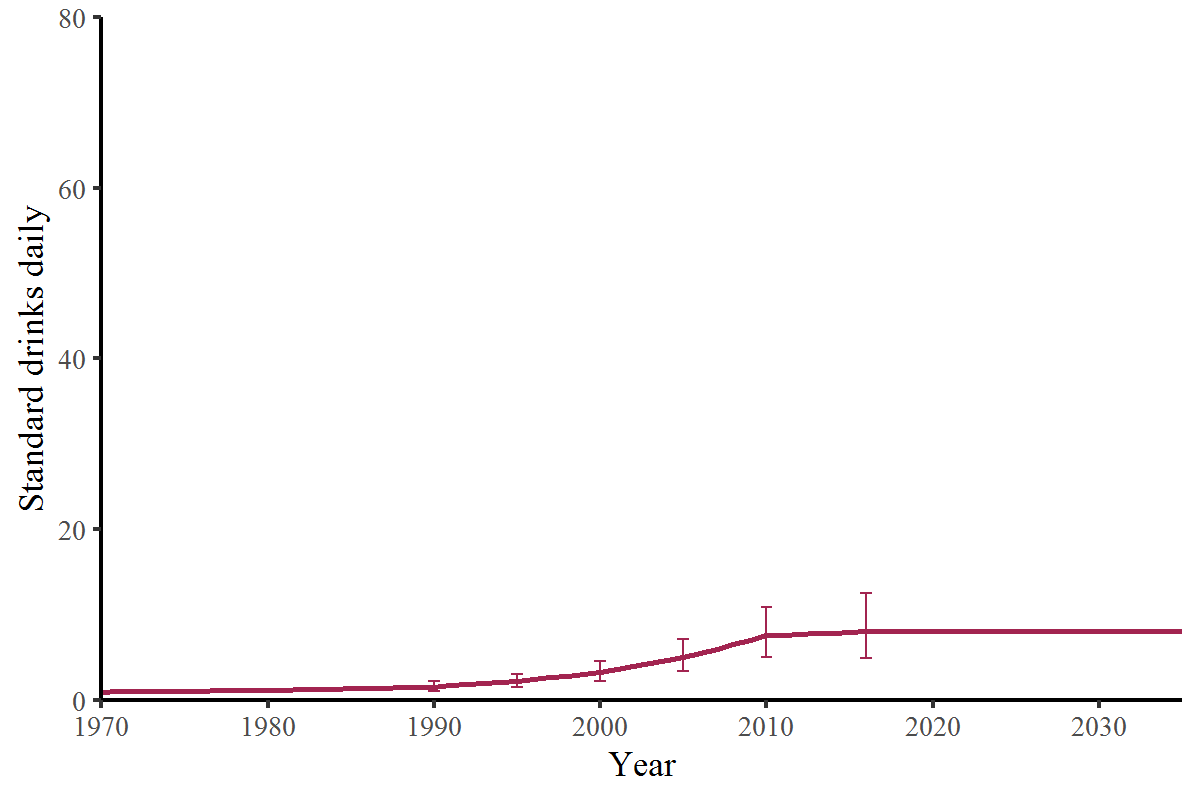


Figure 7: Model priors (line) and IHME estimates (error bars) for alcohol consumption (grams per day) for men (on left in blue) and women (on right in red)

The relative risk of TB disease associated with alcohol consumption is based on a systematic review and meta-analysis (Table 15). The review examined associations between alcohol consumption and TB disease and was not able to differentiate an association with *Mtb* infection from an association with progression from *Mtb* infection to disease. We assume increased risk of progression from *Mtb* infection to disease based on another study that controlled for infection status suggests such a causal pathway [30].

Table 15: Prior ranges and data sources for relative risks of progression from *Mtb* infection to disease among current alcohol drinkers

| Parameter | Description | Prior range  or set value | Reference |
| --- | --- | --- | --- |
| ${RR}_{alc}$ | Relative risk of progression from *Mtb* infection to disease among current alcohol drinkers | 0.004-0.032 | [31] |

Overlap between the proportion of the population classified as tobacco smokers and those classified as alcohol drinkers is not directly acknowledged in the model.

*Adjustments for BCG vaccination*

The relative risk of progression from Mtb infection to disease attributable to BCG vaccination is defined as follows:

$$\mathrm{RR}_{B{CG}_{a}}= {BCG}_{cov}*\left( 1-{BCG}_{{eff}_{a}} \right)+\left( 1-{BCG}_{cov} \right)$$

for age *a*, where${BCG}_{cov}$ is the coverage of BCG vaccination in infants and ${BCG}_{{eff}_{a}}$ is the efficacy of BCG vaccination.

The coverage of BCG vaccination in infants is shown in Figure 8. Vaccine coverage is 0 prior to introduction in 1984 [32] and is then scaled up from 48% in 1984 to 95% in 2018 according to WHO estimates [33]. We assume the average coverage from 2014 through 2018 is maintained through 2035. A 2009 WHO Expanded Programme on Immunizations survey found similar coverage for boys and girls [34]; therefore BCG coverage is assumed equal by sex.


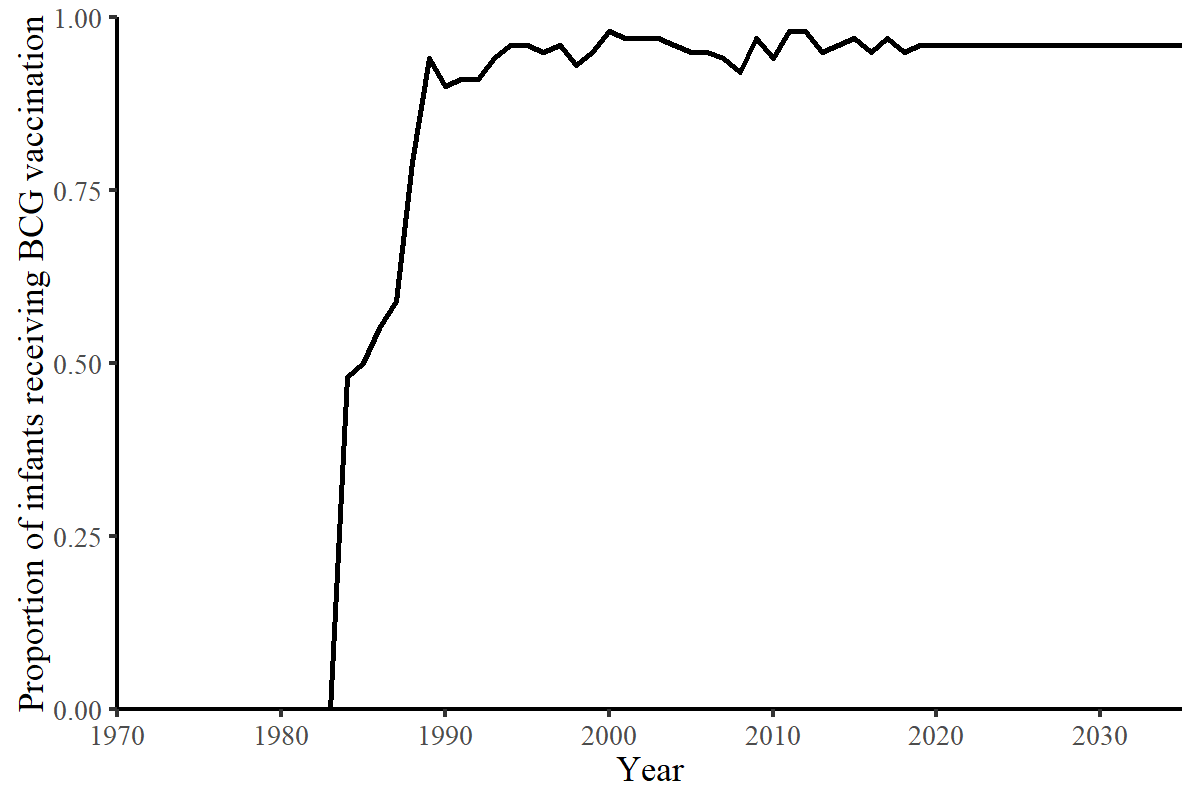


Figure 8: Proportion of infants receiving BCG vaccination

The efficacy of BCG vaccination is shown in Table 16.

Table 16: Prior ranges and data sources for efficacy of BCG vaccination

| Parameter | Description | Prior range  or set value | Reference |
| --- | --- | --- | --- |
| ${BCG}_{{eff}_{a=0-14}}$ | Efficacy of BCG vaccination in ages 0-14 years | 0.39-0.72 | [1, 35] |
| ${BCG}_{{eff}_{a\geq15}}$ | Efficacy of BCG vaccination in ages ≥ 15 years | 0 | Assumption |

HIV-positive strata

The proportion of new infections developing primary disease is adjusted for HIV status following the structure described in Section 3.2.1 using relative risks shown in Table 17.

Table 17: Prior ranges and data sources for risk ratios for proportion of new infections developing primary disease in HIV-positive individuals

| Parameter | Description | Prior range  or set value | Reference |
| --- | --- | --- | --- |
| *RR1_α_* | Risk ratio for new infections developing primary disease in HIV-positive individuals with CD4 > 500 cells/μL | 2.11-3.20 | [1, 25, 36] |
| *RR2_α_* | Risk ratio for new infections developing primary disease in HIV-positive individuals for each 100 cell/μL change in CD4 | 1.30-1.42 | [1] |

ART strata

ART reduces the difference between the CD4-dependent value and the value for an HIV-negative individual, with increasing effect for increasing duration of ART. The proportion of new infections developing primary disease as follows:

$$x_{i,j,l}^{A}=max(\left( x_{i,j}^{H} \right)*\left( 1-{ART}_{l} \right),x_{i})$$

where *A* refers to HIV-positive individuals on ART, *H* refers to HIV-positive individuals not on ART, *i* refers to age, *j* refers to CD4 category, and *l* refers to ART duration such that ART_l_ is the protective effect of ART by ART duration (Table ). The *max* function ensures that ART does not reduce risks below those experienced by HIV-negative individuals of age *i*.

3.2.3 Reactivation rate

The reactivation rate ($\upsilon_{g,a,h,k,t})$ is a time-dependent parameter specific to sex, age, HIV status, and ART duration.

The reactivation rate in HIV-negative individuals is defined as follows:

$$\upsilon_{g,a}=\upsilon_{a}\mathrm{RR}_{s{m\_prog}_{g,a,t}}\mathrm{RR}_{\mathrm{alc}_{g,a,t}}$$

for sex *g*, age *a*, and time-step *t*, where $\upsilon_{a}$ is the base proportion of new infections developing primary disease (Table 18), ${RR}_{s{m\_prog}_{g,a,t}}$ is the relative risk of progression from *Mtb* infection to disease attributable to tobacco smoking (see Section 3.2.2), and ${RR}_{{alc}_{g,a,t}}$ is the relative risk of progression from *Mtb* infection to disease attributable to alcohol consumption (see Section 3.2.2).

Table 18: Prior ranges and data sources for base reactivation rate in HIV-negative individuals

| Parameter | Description | Prior range  or set value | Reference |
| --- | --- | --- | --- |
| $\upsilon_{a\geq15}$ | Base reactivation rate in ages ≥ 15 years | 0.0001-0.0025 | [1, 6, 7, 25, 37] |
| $\upsilon_{a=0-14}$ | Base reactivation rate in ages 0-14 years | 0.0001-0.0025 | [1, 6, 7, 25, 37] |

HIV-positive strata

The reactivation rate is adjusted for HIV status following the structure described in Section 3.2.1 using two relative risks shown in Table 19.

Table 19: Prior ranges and data sources for protective effect of ART on reactivation rate by ART duration

| Parameter | Description | Prior range  or set value | Reference |
| --- | --- | --- | --- |
| *RR1_ν_* | Risk ratio for reactivation rate in HIV-positive individuals with CD4 > 500 cells/μL | 2.11-3.20 | [1, 6, 25, 36, 38] |
| *RR2_ν_* | Risk ratio for reactivation rate in HIV-positive individuals for each 100 cell/μL change in CD4 | 1.30-1.42 | [1, 6, 25, 36, 38] |

ART strata

The reactivation rate is adjusted for ART duration following the structure and protective effects described in Section 3.2.1.

3.3 Infectious disease

3.3.1 Proportion of cases developing smear-positive disease

The proportion of cases developing smear-positive disease ($\sigma_{a,h,k})$ is a constant parameter specific to age, HIV status, and ART duration. Prior ranges and data sources for proportion of cases developing smear-positive disease in HIV-negative individuals are shown in Table 20.

Table 20: Prior ranges and data sources for proportion of cases developing smear-positive disease in HIV-negative individuals

| Parameter | Description | Prior range  or set value | Reference |
| --- | --- | --- | --- |
| $\sigma_{a\geq15}$ | Proportion of cases developing smear-positive disease in ages ≥ 15 years | 0.40-0.80* | [1, 6, 7, 25, 39] |
| $\sigma_{a=0-14}$ | Proportion of cases developing smear-positive disease in ages 10-14 years | 0.228-0.792 | [1, 7, 25, 40] |
| $\sigma_{a=5-9}$ | Proportion of cases developing smear-positive disease in ages 5-9 years | 0.116-0.400 | [1, 7, 25, 40] |
| $\sigma_{a=0-4}$ | Proportion of cases developing smear-positive disease in ages 0-4 years | 0.008-0.024 | [1, 7, 25, 40] |

* $\sigma_{a\geq15}$ must be greater than $\sigma_{a=10-14}$

HIV-positive strata

The proportion of cases developing smear-positive disease in HIV-positive individuals is defined as:

$$\sigma_{a,h=HIV+}=\sigma_{a,h=HIV-} RR\sigma_{HIV}$$

for age *a* and HIV status h, where $\sigma_{a,h=HIV-}$is proportion of cases developing smear-positive disease in HIV-negative individuals and $RR\sigma_{HIV}$ is the relative risk for proportion of cases developing smear-positive disease in HIV-positive individuals (Table 21).

Table 21: Prior ranges and data sources for proportion of cases developing smear-positive disease in HIV-negative individuals

| Parameter | Description | Prior range  or set value | Reference |
| --- | --- | --- | --- |
| $RR\sigma_{HIV}$ | Relative risk for proportion of cases developing smear-positive disease in HIV-positive individuals | 0.548-0.850 | [1, 7] |

3.3.2 Rate of conversion from smear-negative to smear-positive disease

The rate of conversion from smear-negative to smear-positive disease ($\theta_{h})$ is a constant parameter specific to HIV status. Prior ranges and data sources for the rate of conversion from smear-negative to smear-positive disease are shown in Table 22.

Table 22: Prior ranges and data sources for rate of conversion from smear-negative to smear-positive disease

| Parameter | Description | Prior range  or set value | Reference |
| --- | --- | --- | --- |
| $\theta_{h=HIV-}$ | Rate of conversion from smear-negative to smear-positive disease in HIV-negative individuals | 0.007-0.030 | [1, 6, 7, 25] |
| $\theta_{h=HIV+}$ | Rate of conversion from smear negative to smear positive in HIV-positive individuals | 0.015-0.030 | [1] |

3.4 Self-cure

The rate of self-cure ($r_{h})$ is a constant parameter specific to HIV status. Prior ranges and data sources for the rate of self-cure are shown in Table 23.

Table 23: Prior ranges and data sources for rate of self-cure

| Parameter | Description | Prior range  or set value | Reference |
| --- | --- | --- | --- |
| $r_{h=HIV-}$ | Rate of TB self-cure in HIV-negative individuals | 0.10-0.25* | [1, 7, 11, 25, 41] |
| $r_{h=HIV+}$ | Rate of TB self-cure in HIV-positive individuals | 0.06-0.16 | [1, 7, 23] |

* $r_{h=HIV-}$ must be greater than $r_{h=HIV+}$

3.5 Care cascade

3.5.1 Rate of access to TB care

The rate of access to TB care ($\gamma_{g,a,t})$ is a time-dependent parameter specific to sex and age. Rates of access to TB care are defined by generalised logistic functions as follows:

$$\gamma_{g,a,t}=\gamma min\frac{{\gamma max}_{g,a}-\gamma min}{\left( 1+e^{-{\gamma growth}_{g,a}\left( t-{\gamma midyear}_{g,a} \right)} \right)^{\frac{1}{\gamma shape}}}$$

for sex *g*, age *a*, and time-step *t*, where $\gamma min$ is the lower asymptote, ${\gamma max}_{g,a}$ is the upper asymptote, and ${\gamma growth}_{g,a}$, ${\gamma year}_{g,a}$, and $\gamma shape$ are parameters controlling the shape of the function. Rates of access to TB care are assumed the same for HIV-negative and HIV-positive individuals, regardless, among the latter, of ART duration.

Upper asymptote values were determined following the methodology used in Horton et al. [41] to approximate untreated disease duration. We estimate upper and lower limits for 2017 untreated disease duration by sex using results from the second national prevalence survey [42] and relate untreated disease duration to screening rate as follows:

$$\gamma\approx\frac{1}{untreated disease duration*Se*\eta}$$

where γ is the rate of access to TB care , Se is the net sensitivity of the diagnostic algorithm, and η is the proportion of diagnosed individuals linked to care.

Prior ranges and values and data sources for parameters related to the rate of access to TB care are shown in Table 24, and prior curves for rates of access to TB care in men and women are shown in Figure 9.

Table 24: Prior ranges and data sources for parameters related to rate of access to TB care in adults

| Parameter | Description | Prior range  or set value | Reference |
| --- | --- | --- | --- |
| $\gamma shape$ | Shape parameter | 5 | Assumption |
| ${\gamma growth}_{g=M,a\geq15}$ | Growth parameter in men (male, age ≥ 15 years) | 0.5 | Assumption |
| ${\gamma growth}_{g=F,a\geq15}$ | Growth parameter in women (female, age ≥ 15 years) | 0.3 | Assumption |
| ${\gamma growth}_{g,a=0-14}$ | Growth parameter in children (both sexes, age 0-14 years) | 0.5 | Assumption |
| ${\gamma year}_{g=M,a\geq15}$ | Midyear parameter in men (male, age ≥ 15 years) | 1990-2015 | Assumption |
| ${\gamma year}_{g=F,a\geq15}$ | Midyear parameter in women (female, age ≥ 15 years) | 1990-2015 | Assumption |
| ${\gamma year}_{g,a=0-14}$ | Midyear parameter in children (both sexes, age 0-14 years) | 2012 | Assumption |
| $\gamma min$ | Lower asymptote | 0 | Assumption |
| ${\gamma max}_{g=M,a\geq15}$ | Upper asymptote in men (male, age ≥ 15 years) | 1.12-2.53 | [41] |
| ${\gamma max}_{g=F,a\geq15}$ | Upper asymptote in women (female, age ≥ 15 years) | 0.91-2.82 | [41] |
| ${\gamma max}_{g,a=0-14}$ | Upper asymptote in children (both sexes, age 0-14 years) | 0.05-0.20 | Assumption |


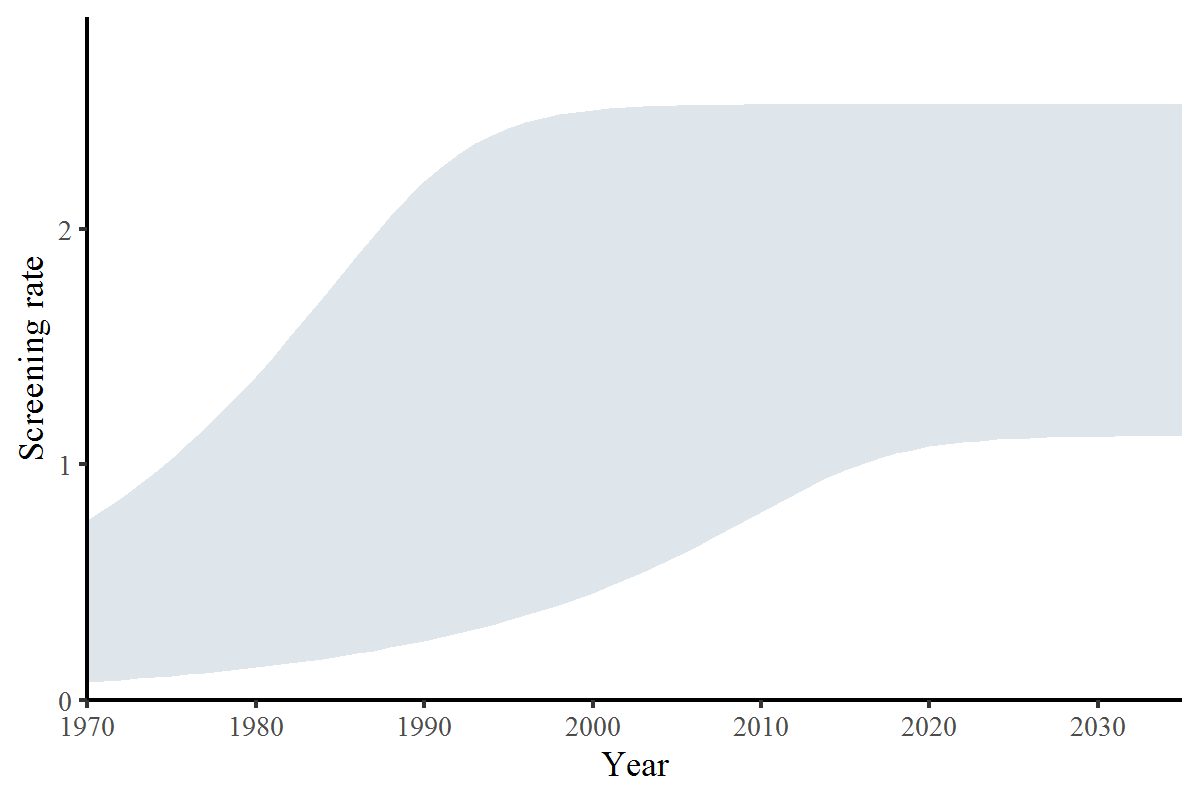

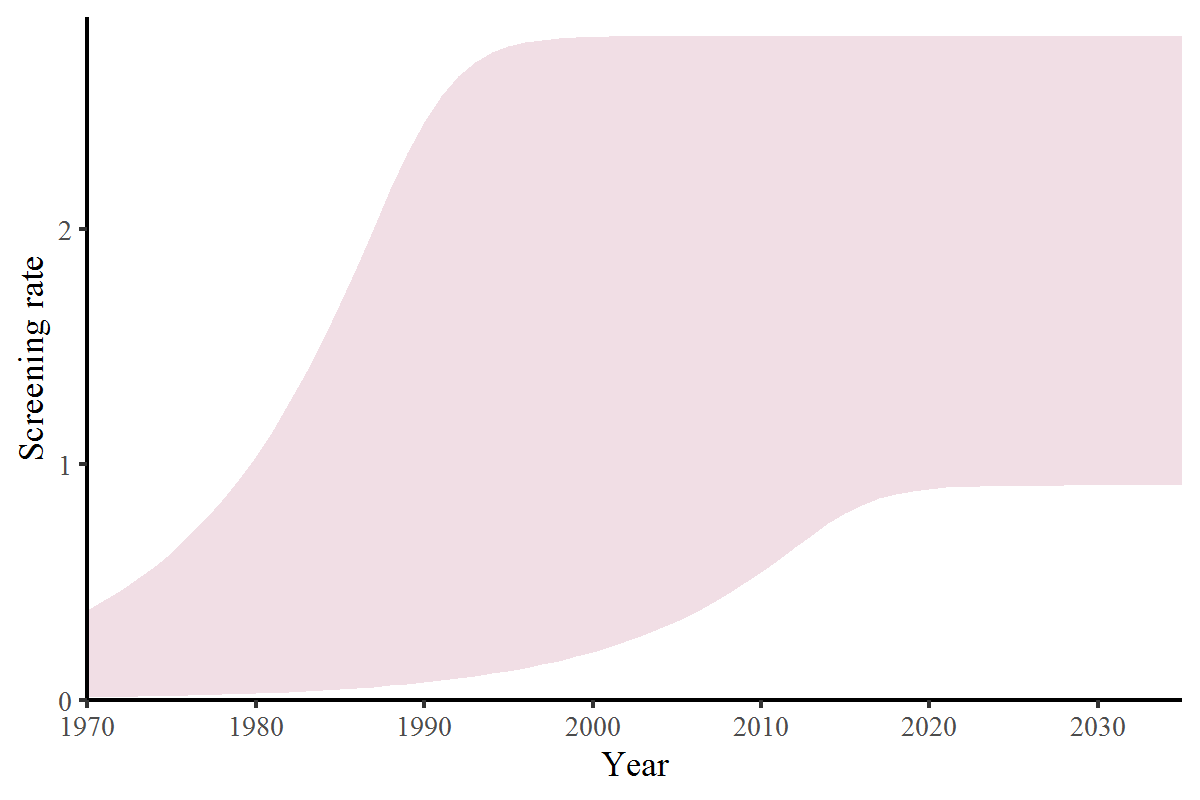


Figure 9: Prior rates of access to TB care ranges for men (on left in blue) and women (on right in red)

Relative rates of access to TB care for individuals with smear-negative TB and for healthy individuals (with neither smear-positive nor smear-negative TB), both relative to individuals with smear-positive TB, are shown in Table 25.

Table 25: Prior ranges and data sources for relative rates of access to TB care in individuals with smear-negative TB and in healthy individuals

| Parameter | Description | Prior range  or set value | Reference |
| --- | --- | --- | --- |
| $d_{N}$ | Relative rate of access to TB care for smear-negative TB compared to smear-positive TB | 0.5 | [43] |
| $d_{health}$ | Relative rate of access to TB care for healthy individuals (with neither smear-positive nor smear-negative TB) relative to smear-positive TB | 0.0058 | [43] |

3.5.2 Diagnostic algorithm

The diagnostic algorithm encompasses the net sensitivity and specificity of diagnostic algorithms for drug susceptible and MDR TB, as well as the coverage of DST. All parameters are time-dependent; the net sensitivity and specificity for drug susceptible TB (${Se}_{I_{h,t}},{Se}_{N_{h,t}}, {Sp}_{h,t}$) are also specific to HIV status. Net sensitivities and specificities are a weighted average based on the coverage of different diagnostic algorithms in a given year under the national guidelines for TB diagnosis in Viet Nam. Values for the net sensitivity, specificity, and DST coverage are shown in Table 26.

Table 26: Values for sensitivity, specificity, and DST coverage in HIV-negative individuals

| Parameter | Description | Years | Prior range  or set value | Reference |
| --- | --- | --- | --- | --- |
| ${Se}_{I_{h=HIV-,t}}$ | Net sensitivity of diagnostic algorithm for drug susceptible, smear-positive TB in HIV-negative individuals | 1970-2035 | 0.5780 | [43] |
| ${Se}_{I_{h=HIV+,t}}$ | Net sensitivity of TB diagnostic algorithm for drug susceptible, smear-positive TB in HIV-positive individuals | 1970-2035 | 0.5780 | [43] |
| ${Se}_{N_{h=HIV-,t}}$ | Net sensitivity of diagnostic algorithm for drug susceptible, smear-negative TB in HIV-negative individuals | 1970-2011 | 0.2400 | [43] |
|  |  | 2012-2035 | 0.2457 |  |
| ${Se}_{N_{h=HIV+,t}}$ | Net sensitivity of TB diagnostic algorithm for drug susceptible, smear-negative TB in HIV-positive individuals | 1970-2011 | 0.2400 | [43] |
|  |  | 2012-2035 | 0.2531 |  |
| ${Sp}_{h=HIV-,t}$ | Net specificity of diagnostic algorithm for drug susceptible TB in HIV-negative individuals | 1970-2011 | 0.9300 | [43] |
|  |  | 2012-2035 | 0.9330 |  |
| ${Sp}_{h=HIV+,t}$ | Net specificity of diagnostic algorithm for drug susceptible TB in HIV-positive individuals | 1970-2011 | 0.9300 | [43] |
|  |  | 2012-2035 | 0.9400 |  |
| $\psi_{X_{t}}$ | Proportion DST coverage in treatment-naïve individuals | 1970-1999 | 0 | [43] |
|  |  | 2000-2035 | 0.0570 |  |
| $\psi_{P_{t}}$ | Proportion DST coverage in previously-treated individuals | 1970-1999 | 0 | [43] |
|  |  | 2000-2035 | 0.0480 |  |
| ${Se}_{R_{t}}$ | Net sensitivity of diagnostic algorithm for MDR TB | 1970-2035 | 1 | [43] |
| ${Sp}_{R_{t}}$ | Net specificity of diagnostic algorithm for MDR TB | 1970-2035 | 1 | [43] |

3.5.3 Linkage to care

The proportion of diagnosed individuals who are linked to treatment for drug-susceptible TB ($\eta_{S_{t}}$) and MDR TB ($\eta_{R_{t}}$) are time-dependent parameters, as shown in Figure 10 [43].


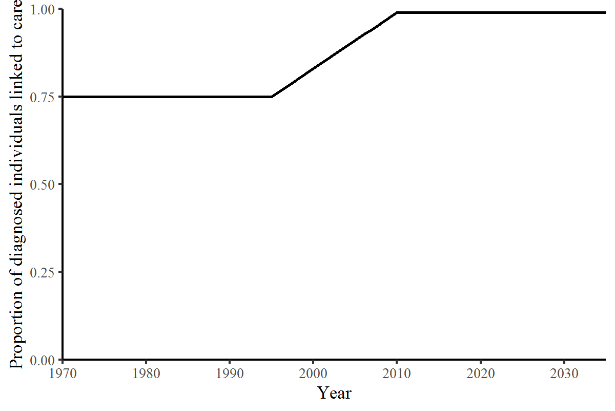

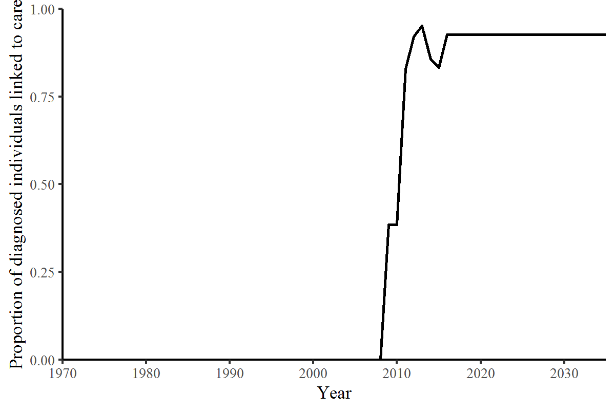


Figure 10: Proportion of diagnosed individuals linked to care for drug-susceptible TB (on left) and MDR TB (on right)

3.5.4 Treatment success

The proportion of individuals linked to care who successfully complete treatment for drug-susceptible TB ($\tau_{S_{h,k,t}}$) and MDR TB ($\tau_{R_{h,k,t}}$) are time-dependent parameters specific to HIV status and ART duration, as shown in Figure 11, Figure 12, and Figure 13 [43].


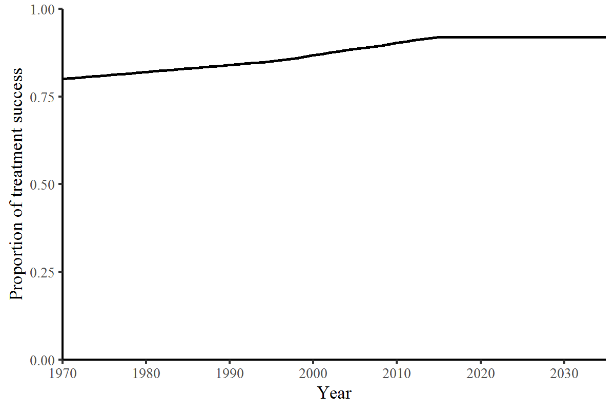

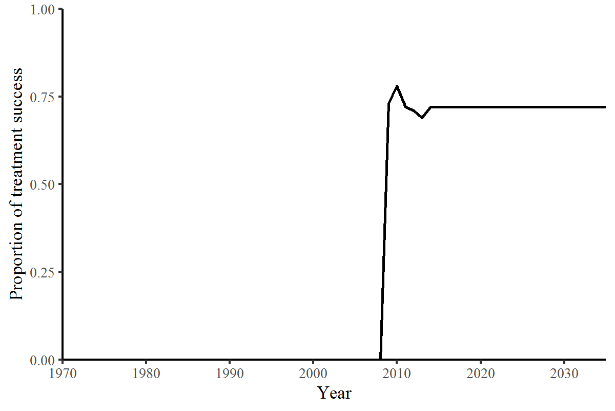


Figure 11: Proportion treatment success in HIV-negative individuals for drug-susceptible TB (on left) and MDR TB (on right)


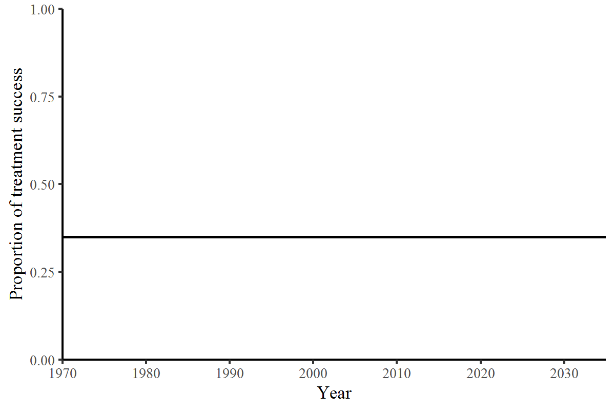

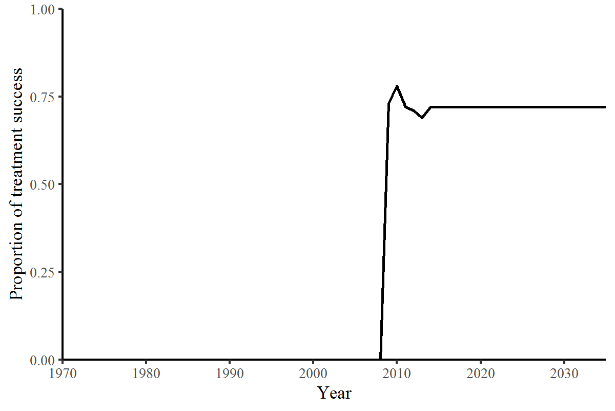


Figure 12: Proportion treatment success in HIV-positive individuals for drug-susceptible TB (on left) and MDR TB (on right)


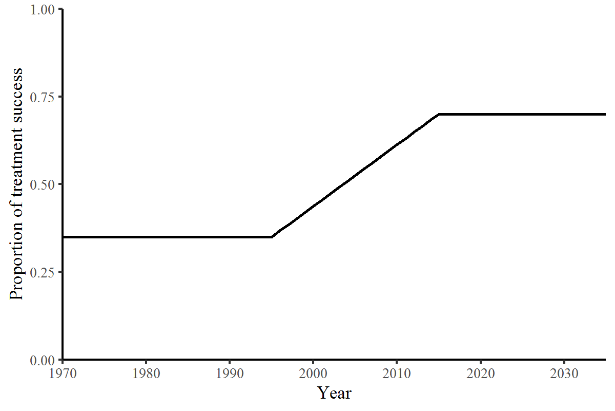

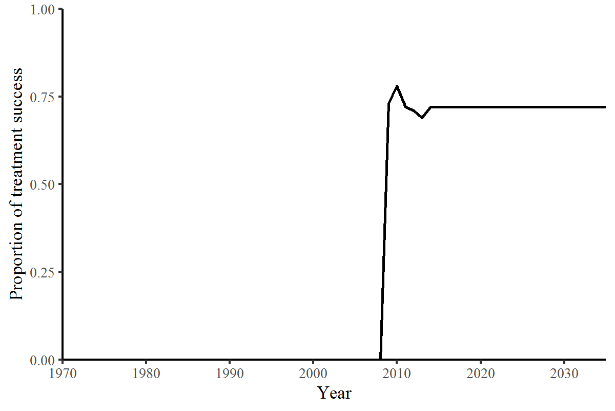


Figure 13: Proportion treatment success in HIV-positive individuals on ART for drug-susceptible TB (on left) and MDR TB (on right)

Prior ranges and data sources for the relative efficacy of first-line treatment for MDR TB in treatment naïve and previously treated individuals are shown in Table 27.

Table 27: Prior ranges and data sources for TB mortality in HIV-negative individuals

| Parameter | Description | Prior range  or set value | Reference |
| --- | --- | --- | --- |
| $\mathrm{RR}_{X}$ | Relative success of using first-line treatment for MDR in treatment naïve individuals | 0.53-0.70 | [1, 44] |
| $\mathrm{RR}_{P}$ | Relative success of using first-line treatment for MDR in previously treated individuals | 0.35-0.58 | [1, 44] |

3.6 Mortality

Mortality rates for smear-positive TB ($\mu_{I_{a,h,k}})$ and smear-negative TB ($\mu_{I_{a,h,k}})$ are constant parameters specific to age, HIV status, and ART duration. Prior ranges and data sources for TB mortality rates in HIV-negative individuals are shown in Table 28.

Table 28: Prior ranges and data sources for TB mortality in HIV-negative individuals

| Parameter | Description | Prior range  or set value | Reference |
| --- | --- | --- | --- |
| $\mu_{I}$ | Smear-positive TB mortality rate | 0.10-0.41 | [1, 7, 23, 25, 41] |
| $\mu_{N}$ | Smear-negative TB mortality rate | 0.09-0.25* | [1, 7, 23, 25, 41] |

* $\mu_{I}$ must be greater than $\mu_{N}$

The mortality rate in individuals ages 0-4 years is defined as:

$\mu_{I_{a=0-4}}=\mu_{I}RR\mu_{I_{0}}$ and $\mu_{N_{a=0-4}}=\mu_{N}RR\mu_{N_{0}}$

for age *a* and HIV status h, where$\mu_{I}$ and $\mu_{N}$ are mortality rates for smear-positive TB and smear-negative TB, respectively, in HIV-negative individuals and $RR\mu_{I_{0}}$ and $RR\mu_{N_{0}}$ are the relative risks for mortality in individuals ages 0-4 years for smear-positive TB and smear-negative TB, respectively (Table 29).

Table 29: Prior ranges and data sources for TB mortality in HIV-negative individuals

| Parameter | Description | Prior range  or set value | Reference |
| --- | --- | --- | --- |
| $RR\mu_{I_{0}}$ | Relative risk of smear-positive TB mortality in ages 0-4 years | 1.70-2.98 | [1] |
| $RR\mu_{N_{0}}$ | Relative risk of smear-negative TB mortality in ages 0-4 years | 1.70-2.98 | [1] |

HIV-positive strata

The mortality rate in HIV-positive individuals is defined as:

$$\mu_{a,h=HIV+}=\mu_{a,h=HIV-} RR\mu_{HIV}$$

for age *a* and HIV status h, where $\mu_{a,h=HIV-}$is mortality rate in HIV-negative individuals and $RR\mu_{HIV}$ is the relative risk for mortality in HIV-positive individuals (Table 30).

Table 30: Prior ranges and data sources for TB mortality in HIV-negative individuals

| Parameter | Description | Prior range  or set value | Reference |
| --- | --- | --- | --- |
| *RRμ_HIV_* | Relative risk of smear-positive or smear-negative TB mortality in HIV-positive individuals | 2 | [1] |

ART strata

The reactivation rate is adjusted for ART duration following the structure described in Section 3.2.1 and protective effects in Table 31.

Table 31: Protective effect of ART by ART duration

| Parameter | Description | Prior range  or set value | Reference |
| --- | --- | --- | --- |
| ${ART}_{\mu_{l<6}}$ | Protective effect of ART for duration < 6 months | 0.11-0.28 | [1, 24] |
| ${ART}_{\mu_{l=6-12}}$ | Protective effect of ART for duration 6-12 months | 0.51-0.75 | [1, 24] |
| ${ART}_{\mu_{l\geq12}}$ | Protective effect of ART for duration ≥ 12 months | 0.64-0.95* | [1, 24] |

* ${ART}_{l\geq12}$ must be greater than ${ART}_{l=6-12}$

3.7 MDR

The movement of individuals with latent superinfection is determined by parameter $\iota=\frac{\varphi}{1+\varphi}$ with φ as defined in Section 3.1.

The rate of acquisition of MDR during treatment ($\xi_{h})$ is specific to HIV status. Prior ranges and data sources for the rate of acquisition of MDR during treatment are shown in Table 32.

Table 32: Rate of acquisition of MDR during treatment in HIV-negative individuals by HIV status

| Parameter | Description | Prior range  or set value | Reference |
| --- | --- | --- | --- |
| $\xi$ | Rate of acquisition of MDR during treatment in HIV-negative individuals | 0.010-0.017 | [1, 45] |
| $\xi_{HIV}$ | Rate of acquisition of MDR during treatment in HIV-positive individuals | 0.010-0.017 | [1, 45] |

4 Model implementation

The model is initialised with the 1970 population with sex and age structure and 50 smear-positive, drug-susceptible, treatment-naïve TB cases in the age group 20-24 years. The model is run for 200 years with all parameters set at 1970 values and no HIV.

The resulting population is then scaled to the 1970 population, and the model is run through 2035 with time-dependent parameter values and HIV and ART.

The model is implemented in R [46]. Model equations are implemented as ordinary differential equations with a time step of 0.5 years and are solved using the fourth order Runge-Kutta integration method [47].

5 Calibration

The model was calibrated to demographic and epidemiologic targets in a two phase approach.

**5.1 Population calibration**

The model was first manually calibrated to demographic data on population size estimates from UN World Population Prospects [15]. Calibration targets included estimates for total population, men, women, and children for 2000, 2005, 2010, and 2015, as well as projections for 2035 (Table 33). Model estimates fell within 10% of each point estimate for population size.

Table 33: Demographic calibration targets

| Demographic | Population size (in thousands) | | | | |
| --- | --- | --- | --- | --- | --- |
|  | 2000 | 2005 | 2010 | 2015 | 2035 |
| Total | 79 910 | 83 833 | 87 968 | 92 677 | 106 296 |
| Men | 26 635 | 29 856 | 32 951 | 35 013 | 42 217 |
| Women | 28 045 | 31 256 | 34 232 | 36 321 | 43 495 |
| Children | 25 231 | 22 720 | 20 784 | 21 343 | 20 584 |

**5.2 Epidemiological calibration**

The model was calibrated to a set of epidemiological targets to reflect the magnitude and time trends of the TB epidemic (Table 34) using an adaptive approximate Bayesian computation (ABC) Markov chain Monte Carlo (MCMC) method [48] with a modified version of the easyABC package that accepts seed parameter values [49] in R [46]. MCMC was initially seeded with the 14 best-fitting parameter sets from 2 million random parameter sets. Acceptance criterion were increased iteratively from 30 to 37 calibration targets, with the best fits from each iteration used to seed chains in subsequent iterations. Epidemiological calibration was reached when we sampled from a posterior distribution consistent with all 37 epidemiological targets .

Table 34: Epidemiological calibration targets

| Category | Calibration target | Year | Range | Reference |
| --- | --- | --- | --- | --- |
| Incidence | Incidence per 100,000 | 2000 | 157-479 | [50] |
|  |  | 2010 | 143-342 |  |
|  |  | 2018 | 116-263 |  |
|  | HIV-positive incidence per 100,000 | 2018 | 4-9 | [50] |
|  | Proportion of incidence in children * | 2018 | 0.04-0.06 | [50] |
| Mortality | Mortality per 100,000 † | 2000 | 25-59 | [50] |
|  |  | 2010 | 18-40 |  |
|  |  | 2018 | 9-25 |  |
|  | Mortality per 100,000  in HIV-positive individuals | 2018 | 1.5-3.4 | [50] |

| Category | Calibration target | Year | Range | Reference |
| --- | --- | --- | --- | --- |
| Case notification rate | Total case notification rate per 100,000 ‡ | 2000 | 112-176 | [51, 52] |
|  |  | 2010 | 111-173 |  |
|  |  | 2018 | 104-163 |  |
|  | New smear-positive case notification rate per 100,000 in men § | 2000 | 136-175 | [51, 52] |
|  |  | 2010 | 117-151 |  |
|  | New and relapse case notification rate per 100,000 in men § | 2018 | 197-253 | [51, 52] |
|  | New smear-positive case notification rate per 100,000 in women § | 2000 | 60-93 | [51, 52] |
|  |  | 2010 | 39-62 |  |
|  | New and relapse case notification rate per 100,000 in women § | 2018 | 72-112 | [51, 52] |
|  | Proportion of case notifications  in children < 15 years of age | 2015 | 0.01-0.02 | [51] |
|  | Proportion of case notifications  in HIV-positive individuals ‖ | 2015 | 0.015-0.060 | [43] |
| Prevalence | Bacteriologically positive prevalence  per 100,000 adults | 2007 | 415-507 | [53] |
|  |  | 2017 | 260-399 | [42] |
|  | Bacteriologically positive prevalence  per 100,000 men ¶ | 2007 | 697-818 | [53] |
|  |  | 2017 | 420-648 | [42] |
|  | Bacteriologically positive prevalence  per 100,000 women ¶ | 2007 | 168-266 | [53] |
|  |  | 2017 | 89-198 | [42] |
|  | Smear-positive prevalence  per 100,000 adults | 2017 | 55-115 | [42] |
|  | Smear-positive prevalence  per 100,000 men | 2017 | 78-180 | [42] |
|  | Smear-positive prevalence  per 100,000 women | 2017 | 23-79 | [42] |
| M:F ratios | M:F ratio in total prevalence | 2007 | 2.6-4.9 | [53] |
|  |  | 2017 | 2.8-5.8 | [42] |
|  | M:F ratio smear-positive prevalence | 2017 | 1.4-5.6 | [42] |
|  | M:F ratio in *Mtb* infection prevalence | 2015 | 1.4-2.2 | [54] |
| MDR | Proportion MDR in new cases | 2005 | 0.020-0.037 | [43] |
|  |  | 2011 | 0.025-0.054 |  |
|  | Proportion MDR in retreatment cases | 2005 | 0.140-0.250 |  |
|  |  | 2011 | 0.167-0.299 |  |

* Calculated as incidence in ages 0-14 years divided by total incidence with range ± 20%

† Upper limit for 2018 extended to match linear trend from previous (2000 and 2010) estimates

‡ Calculated as total new and relapse cases and cases with unknown previous TB treatment history, divided by population estimate, with ranges assuming 70-95% of cases reported to NTP

§ Ranges assuming 70-95% of cases reported to NTP

‖ Range ± 50%

¶ Estimates for 2007 calculated by applying upper and lower limit of M:F ratio to upper and lower limit of revised estimate for overall prevalence

6 Calibrated model

6.1 Posterior parameters

Median values and ranges for posterior parameter estimates are summarised, with prior ranges for comparison, in Table 35.

Table 35: Summary of prior and posterior parameter values

| Parameter | Parameter definition | Prior range | Posterior median  and range |
| --- | --- | --- | --- |
| *c* | Relative infectiousness of smear-negative TB compared to smear-positive TB | 0.10-0.37 | 0.25 (0.10-0.36) |
| *cscal* | Scaling factor to adjust the total number of contacts | 6-12 | 10.67 (8.54-11.99) |
| φ | Relative fitness of MDR strains compared to drug-susceptible strains | 0.58-0.85 | 0.75 (0.65-0.85) |
| ${RR}_{sm\_inf}$ | Relative risk of *Mtb* infection among current smokers | 1.46-2.30 | 2.19 (1.69-2.30) |
| $\mathrm{RR}_{\mathrm{sex}_{g=M,a\geq15}}$ | Relative risk of infection attributable to additional sex- or gender-based risks in men | 1-2 | 1.57 (1.35-1.94) |
| ${amp}_{HIV}$ | Scaling factor to amplify contacts between HIV-positive individuals | 1-5 | 4.47 (3.46-4.99) |
| $x_{a\geq15}$ | Proportion protection due to previous infection against progression to active disease following reinfection for age ≥ 15 years | 0.37-0.90 | 0.50 (0.37-0.64) |
| $x_{a=0-14}$ | Proportion protection due to previous infection against progression to active disease following reinfection for ages 0-14 years | 0.37-0.90 | 0.54 (0.37-0.78) |
| *RR1_x_* | Risk ratio for protection due to previous infection against progression to active disease following reinfection in HIV-positive individuals with CD4 > 500 cells/μL | 0.60-1.00 | 0.82 (0.60-0.99) |
| *RR2_x_* | Risk ratio for protection due to previous infection against progression to active disease following reinfection in HIV-positive individuals for each 100 cell/μL change in CD4 | 0.50-1.00 | 0.58 (0.50-0.72) |
| ${ART}_{{TB}_{l<6}}$ | Protective effect of ART for duration < 6 months | 0.16-0.27 | 0.21 (0.16-0.27) |
| ${ART}_{{TB}_{l=6-12}}$ | Protective effect of ART for duration 6-12 months | 0.43-0.73 | 0.46 (0.43-0.59) |
| ${ART}_{{TB}_{l\geq12}}$ | Protective effect of ART for duration ≥ 12 months | 0.54-0.92 | 0.66 (0.54-0.87) |
| $\alpha_{a\geq15}$ | Base proportion of new infections developing primary disease in ages ≥ 15 years | 0.08-0.15 | 0.11 (0.08-0.14) |
| $\alpha_{a=10-14}$ | Base proportion of new infections developing primary disease in ages 10-14 years | 0.032-0.104 | 0.059 (0.032-0.098) |

| Parameter | Parameter definition | Prior range | Posterior median  and range |
| --- | --- | --- | --- |
| $\alpha_{a=5-9}$ | Base proportion of new infections developing primary disease in ages 5-9 years | 0.080-0.260 | 0.182 (0.081-0.240) |
| $\alpha_{a=0-4}$ | Base proportion of new infections developing primary disease in ages 0-4 years | 0.151-0.495 | 0.248 (0.159-0.365) |
| ${RR}_{sm\_prog}$ | Relative risk of progression from *Mtb* infection to disease among current smokers | 1.46-2.30 | 1.21 (1.14-1.58) |
| ${RR}_{alc}$ | Relative risk of progression from *Mtb* infection to disease among current alcohol drinkers | 0.004-0.032 | 0.014 (0.007-0.020) |
| ${BCG}_{eff}$ | Efficacy of BCG vaccination | 0.39-0.72 | 0.42 (0.39-0.52) |
| *RR1_α_* | Risk ratio for new infections developing primary disease in HIV-positive individuals with CD4 > 500 cells/μL | 2.11-3.20 | 2.78 (2.18-3.19) |
| *RR2_α_* | Risk ratio for new infections developing primary disease in HIV-positive individuals for each 100 cell/μL change in CD4 | 1.30-1.42 | 1.38 (1.33-1.42) |
| $\upsilon_{a\geq15}$ | Base reactivation rate in ages ≥ 15 years | 0.0001-0.0025 | 0.0013 (0.0008-0.0019) |
| $\upsilon_{a=0-14}$ | Base reactivation rate in ages 0-14 years | 0.0001-0.0025 | 0.0010 (0.0001-0.0025) |
| *RR1_ν_* | Risk ratio for reactivation rate in HIV-positive individuals with CD4 > 500 cells/μL | 2.11-3.20 | 2.61 (2.11-3.19) |
| *RR2_ν_* | Risk ratio for reactivation rate in HIV-positive individuals for each 100 cell/μL change in CD4 | 1.30-1.42 | 1.32 (1.30-1.38) |
| $\sigma_{a\geq15}$ | Proportion of cases developing smear-positive disease in ages ≥ 15 years | 0.40-0.80 | 0.58 (0.49-0.64) |
| $\sigma_{a=10-14}$ | Proportion of cases developing smear-positive disease in ages 10-14 years | 0.228-0.792 | 0.425 (0.229-0.595) |
| $\sigma_{a=5-9}$ | Proportion of cases developing smear-positive disease in ages 5-9 years | 0.116-0.400 | 0.140 (0.116-0.221) |
| $\sigma_{a=0-4}$ | Proportion of cases developing smear-positive disease in ages 0-4 years | 0.008-0.024 | 0.010 (0.008-0.019) |
| $RR\sigma_{HIV}$ | Relative risk for proportion of cases developing smear-positive disease in HIV-positive individuals | 0.548-0.850 | 0.76 (0.55-0.85) |
| $\theta_{h=HIV-}$ | Rate of conversion from smear-negative to smear-positive disease in ages ≥ 15 years | 0.007-0.030 | 0.019 (0.010-0.030) |
| $\theta_{h=HIV+}$ | Rate of conversion from smear negative to smear positive in HIV-positive individuals | 0.015-0.030 | 0.023 (0.015-0.029) |
| $r_{h=HIV-}$ | Rate of TB self-cure in HIV-negative individuals | 0.10-0.25 | 0.13 (0.10-0.17) |
| $r_{h=HIV+}$ | Rate of TB self-cure in HIV-positive individuals | 0.06-0.16 | 0.07 (0.06-0.11) |
| ${\gamma max}_{g=M,a\geq15}$ | Upper asymptote in men (male, age ≥ 15 years) | 1.12-2.53 | 1.45 (1.18-1.78) |
| ${\gamma max}_{g=F,a\geq15}$ | Upper asymptote in women (female, age ≥ 15 years) | 0.91-2.82 | 1.37 (1.01-1.63) |
| ${\gamma max}_{a=0-14}$ | Upper asymptote in ages 0-14 years | 0.05-0.20 | 0.13 (0.10-0.20) |
| ${\gamma year}_{g=M,a\geq15}$ | Midyear parameter in men (male, age ≥ 15 years) | 1990-2015 | 2010 (2005-2015) |
| Parameter | Parameter definition | Prior range | Posterior median  and range |
| ${\gamma year}_{g=F,a\geq15}$ | Midyear parameter in women (female, age ≥ 15 years) | 1990-2015 | 2000 (1990-2007) |
| $\mu_{I}$ | Smear-positive TB mortality rate | 0.10-0.41 | 0.11 (0.10-0.13) |
| $\mu_{N}$ | Smear-negative TB mortality rate | 0.09-0.25 | 0.09 (0.09-0.10) |
| *RRμ_I0_* | Relative risk of smear-positive TB mortality in ages 0-4 years | 1.70-2.98 | 2.34 (1.92-2.78) |
| *RRμ_N0_* | Relative risk of smear-negative TB mortality in ages 0-4 years | 1.70-2.98 | 2.10 (1.70-2.73) |
| ${ART}_{\mu_{l<6}}$ | Protective effect of ART for duration < 6 months | 0.11-0.28 | 0.26 (0.18-0.28) |
| ${ART}_{\mu_{l=6-12}}$ | Protective effect of ART for duration 6-12 months | 0.51-0.75 | 0.67 (0.51-0.75) |
| ${ART}_{\mu_{l\geq12}}$ | Protective effect of ART for duration ≥ 12 months | 0.64-0.95 | 0.82 (0.69-0.95) |
| $\xi$ | Rate of acquisition of MDR during treatment in HIV-negative individuals | 0.010-0.017 | 0.014 (0.011-0.017) |
| $\xi_{HIV}$ | Rate of acquisition of MDR during treatment in HIV-positive individuals | 0.010-0.017 | 0.012 (0.010-0.017) |
| ${RR}_{X}$ | Relative success of using first-line treatment for MDR in treatment naïve individuals | 0.53-0.70 | 0.66 (0.56-0.70) |
| ${RR}_{P}$ | Relative success of using first-line treatment for MDR in previously treated individuals | 0.35-0.58 | 0.46 (0.35-0.56) |


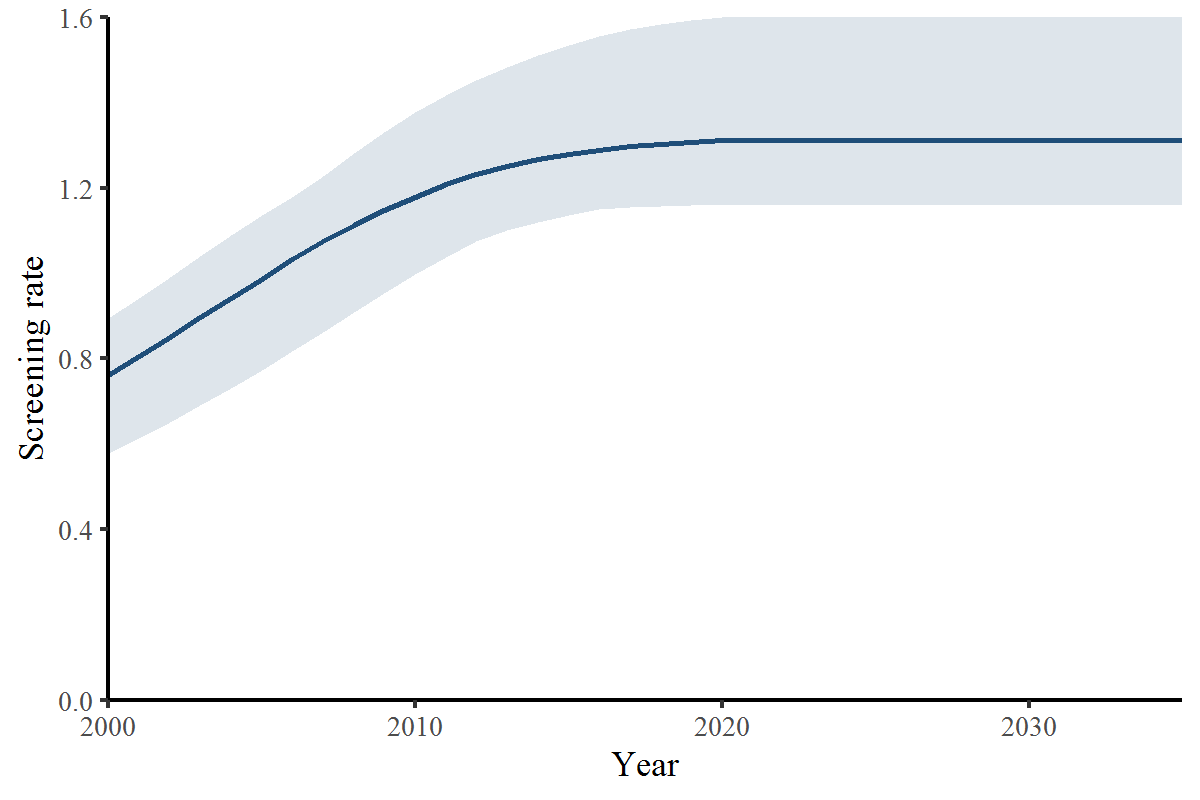

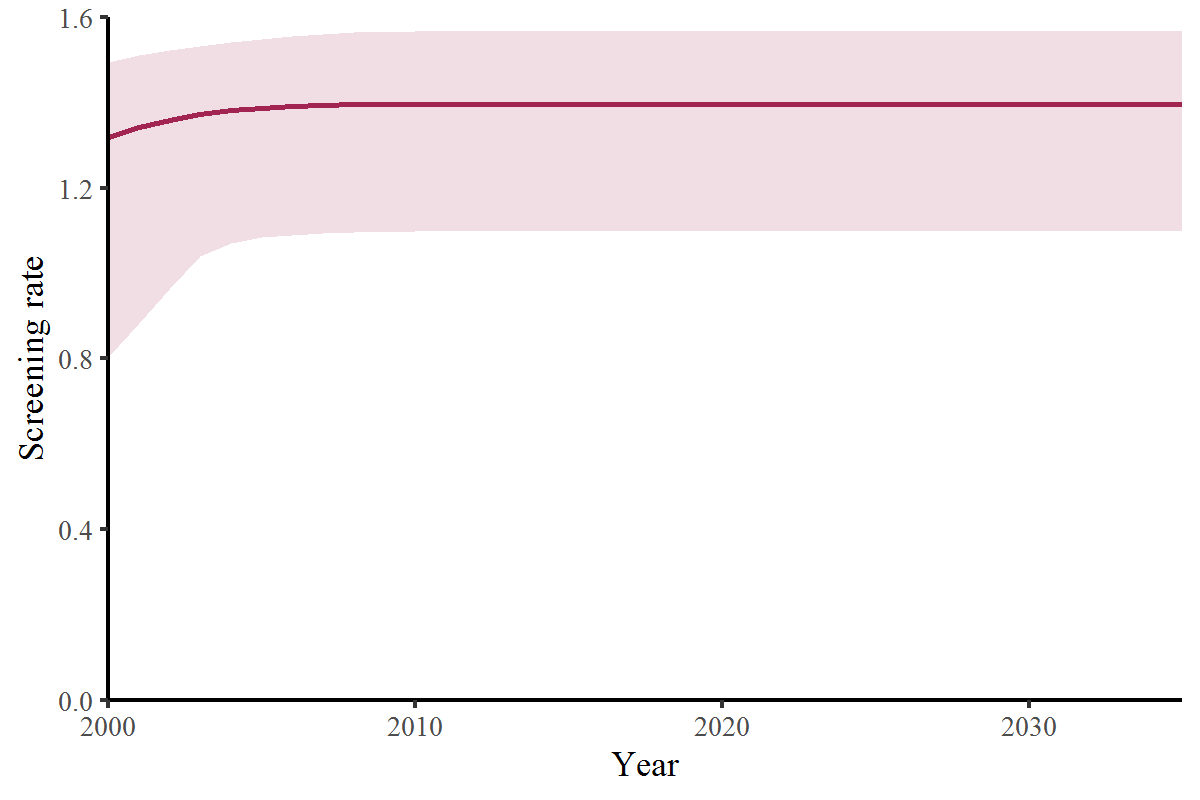


Figure 14: Rate of access to TB care for men (blue on left) and women (red on right) for the calibrated model. Figure shows median values (line) and range (shaded area).

6.2 Population calibration


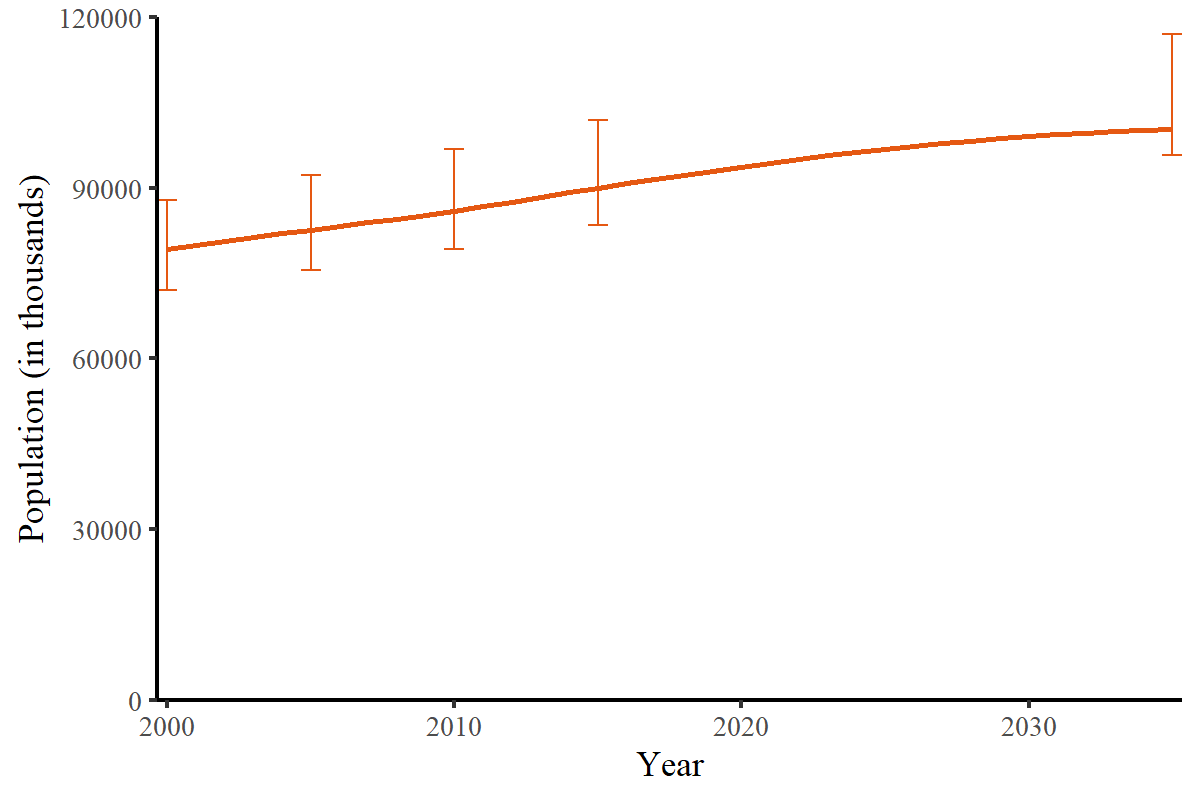


Figure 15: Population for total population for the calibrated model. Figure shows median model estimates (line) and calibration targets (error bars).


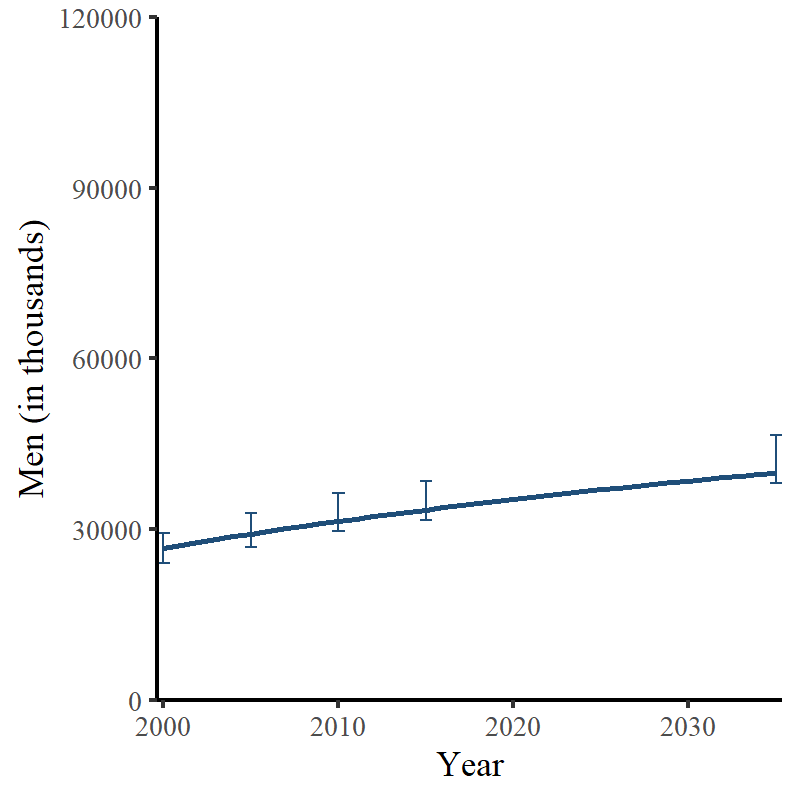

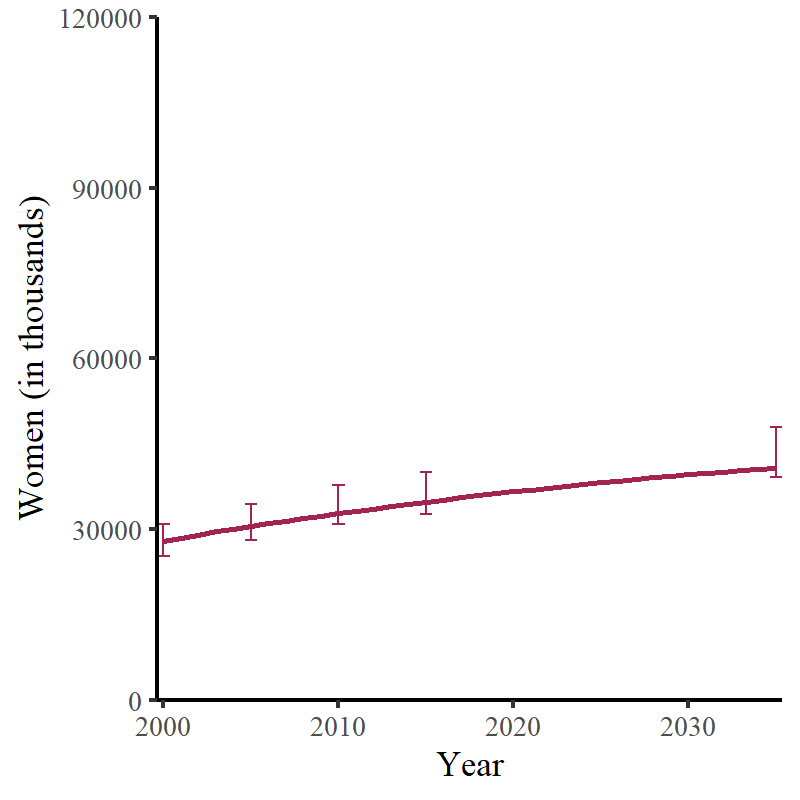

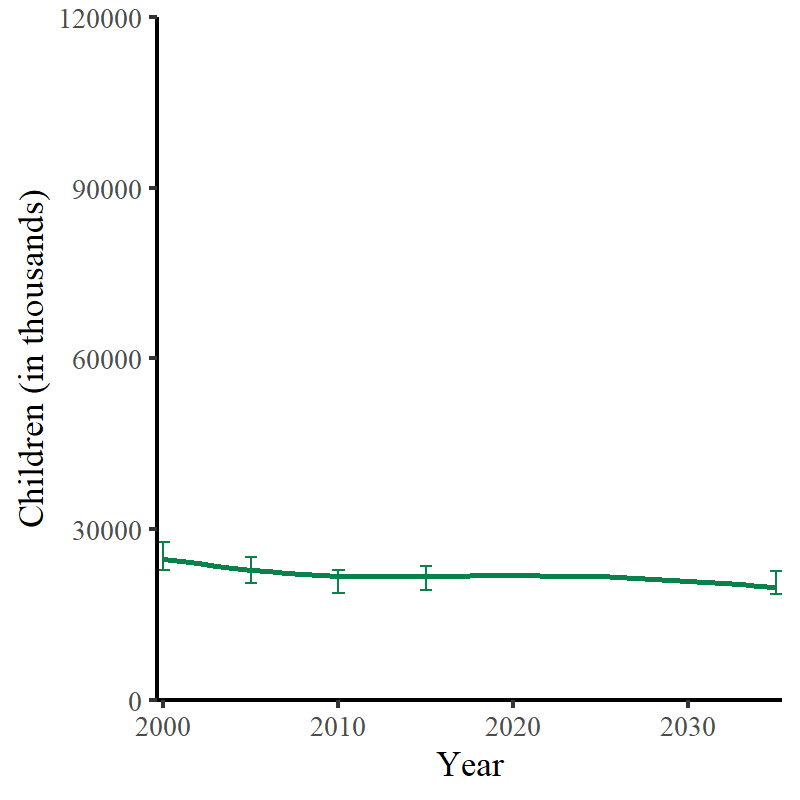


**Figure 16:** **Population for men (left in blue), women (centre in red), and children (right in green) for the calibrated model. Figure shows median model estimates (line) and calibration targets (error bars).**

6.3 Epidemiological calibration

6.3.1 Incidence


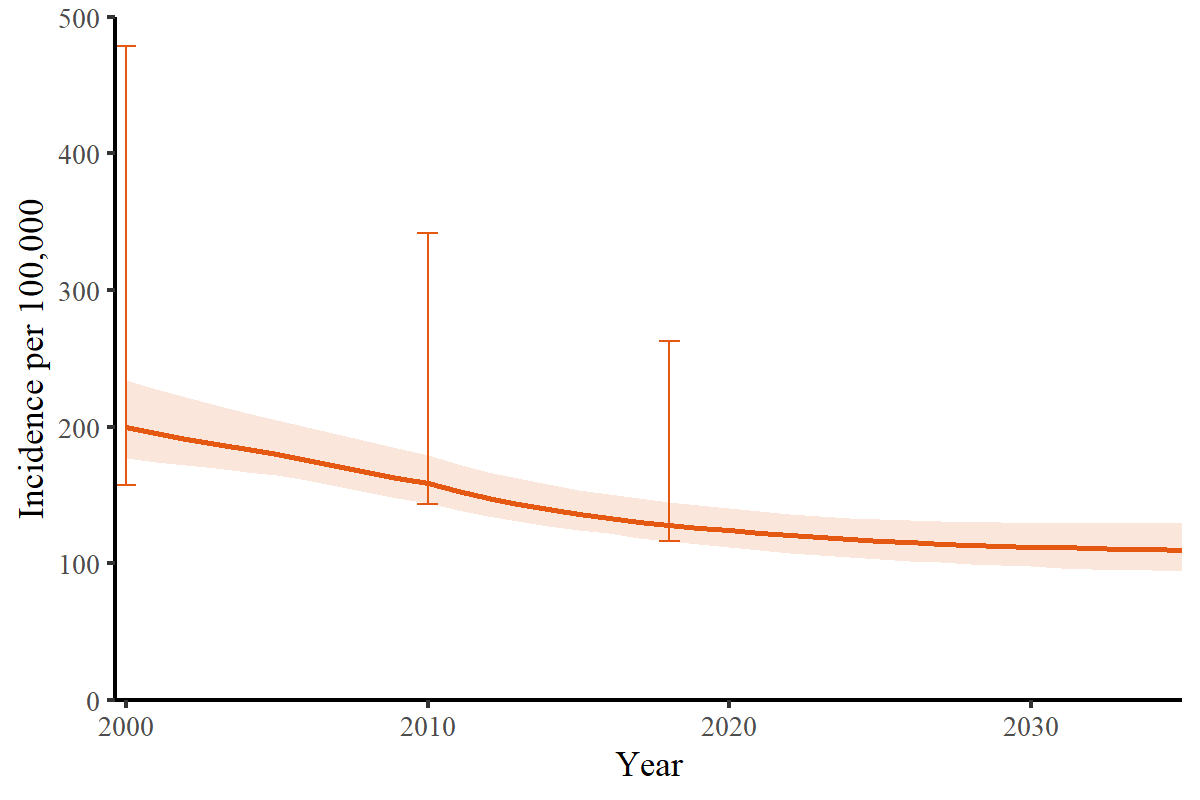


Figure 17: Incidence for total population for the calibrated model. Figure shows median model estimates (line), model uncertainty (shaded area), and calibration targets (error bars).


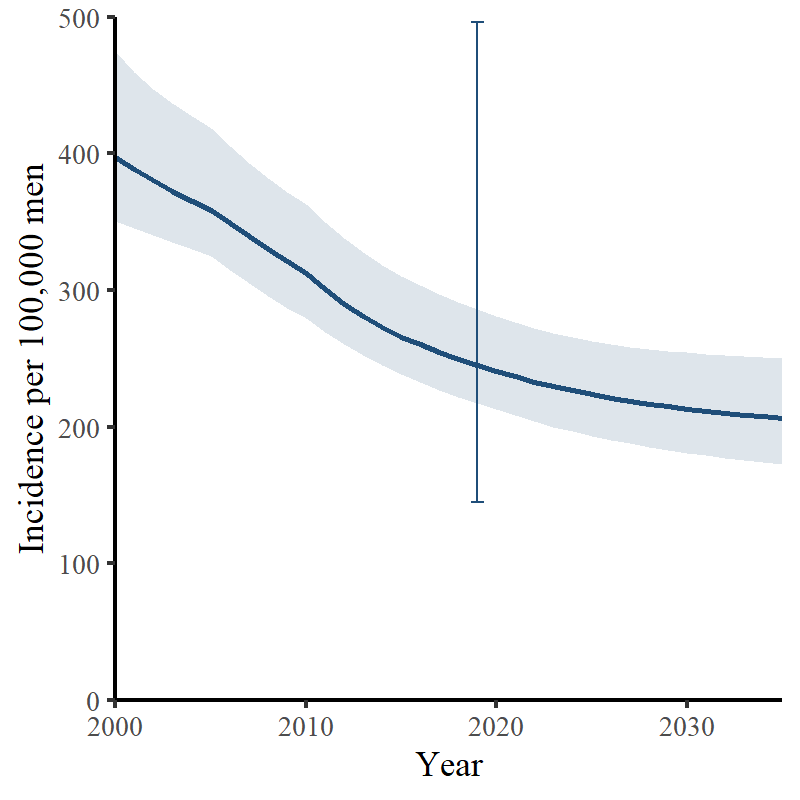

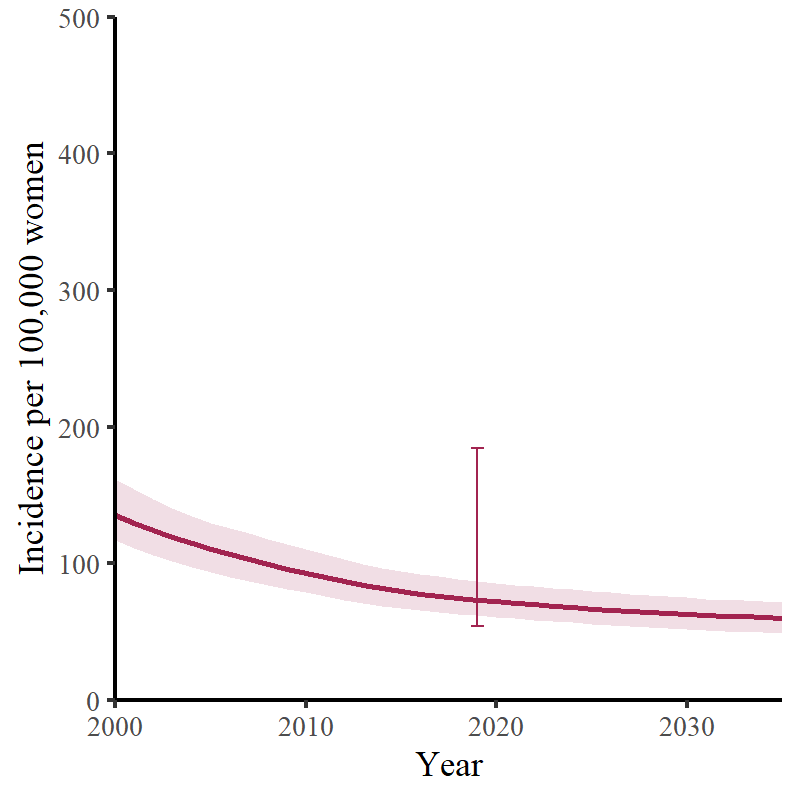

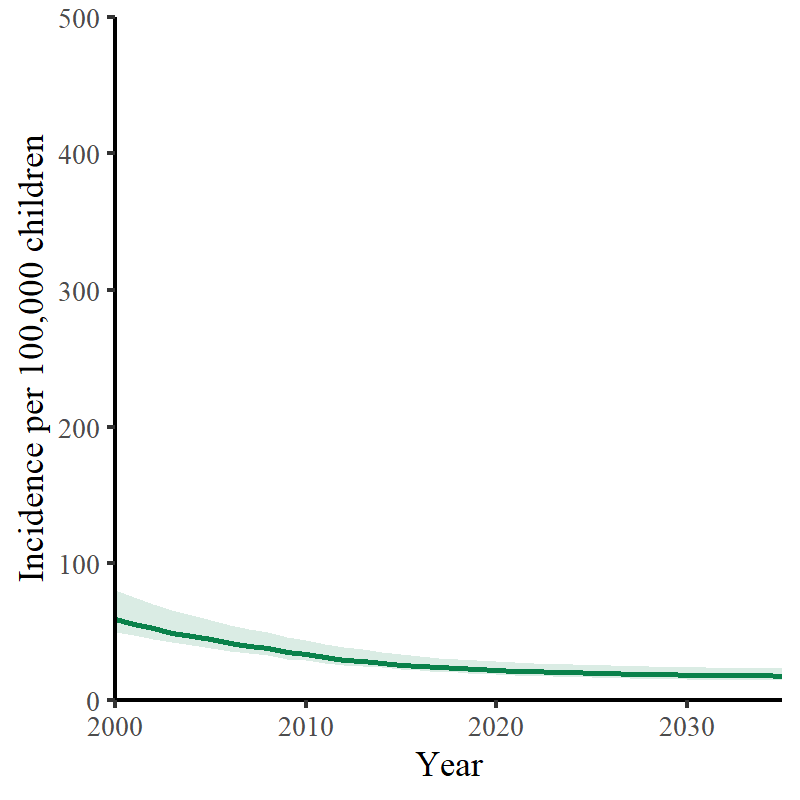


Figure 18: Incidence for men (left in blue), women (centre in red), and children (right in green) for the calibrated model. Figures show median model estimates (line) and model uncertainty (shaded area).


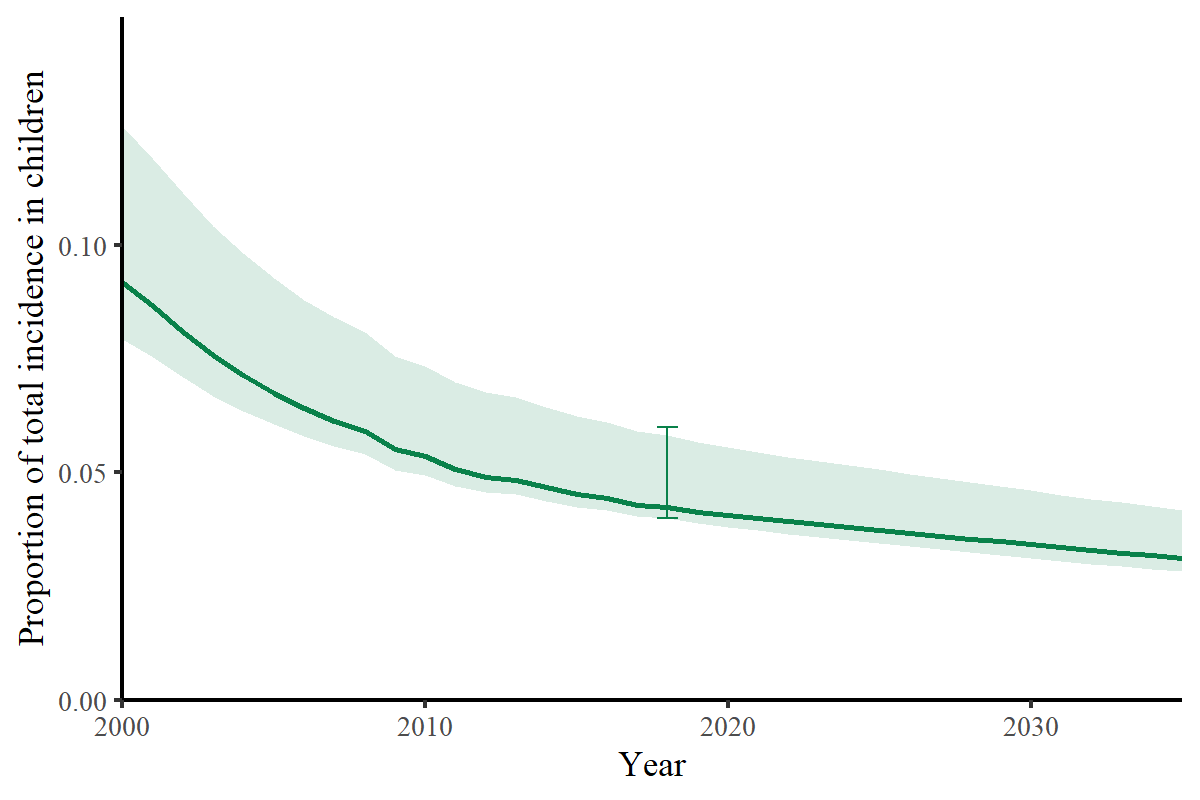


Figure 19: Proportion of total incidence in children for the calibrated model. Figure shows median model estimates (line), model uncertainty (shaded area), and calibration targets (error bars).


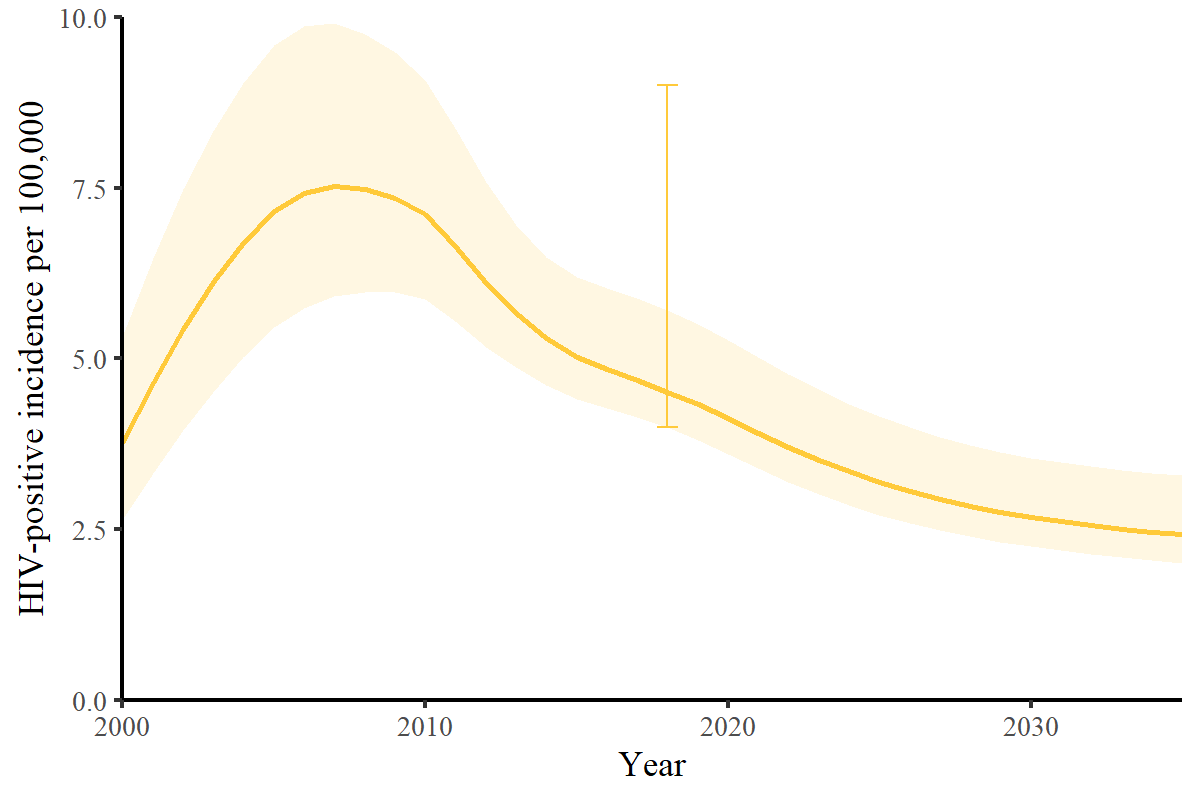


Figure 20: HIV-positive TB incidence for the calibrated model. Figure shows median model estimates (line), model uncertainty (shaded area), and calibration targets (error bars).


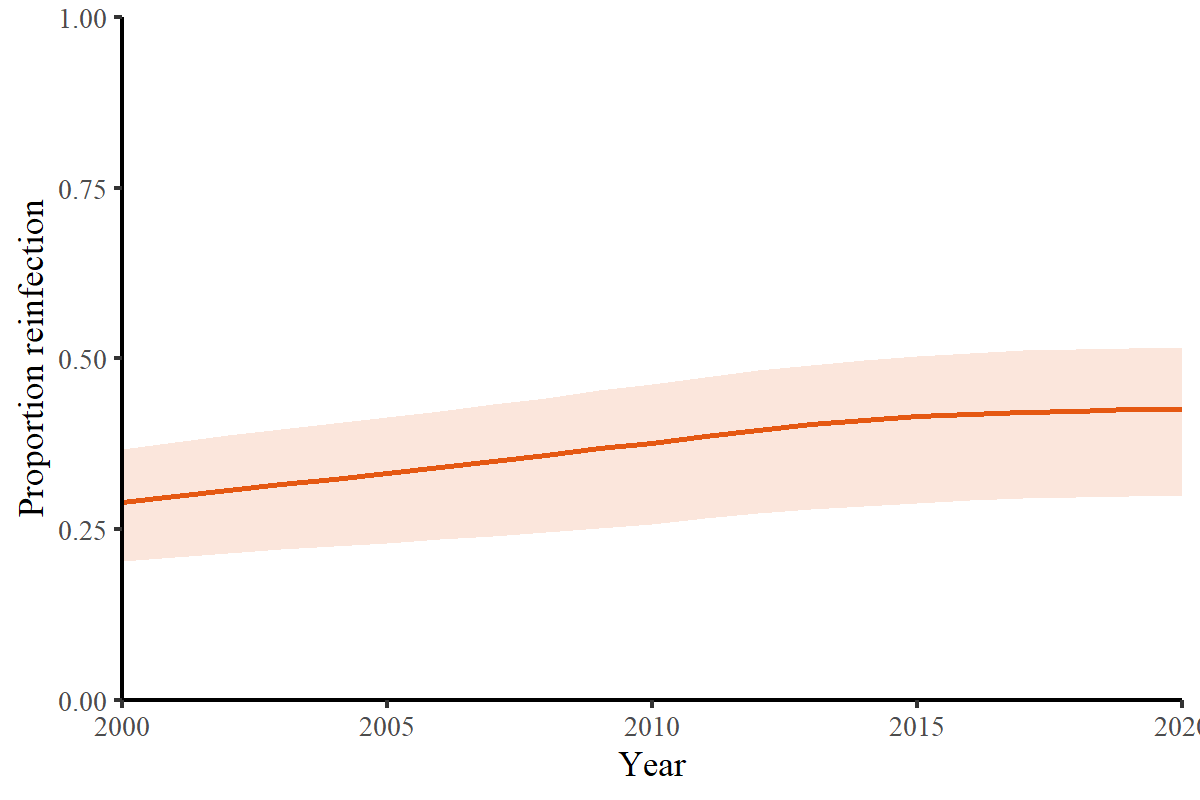


Figure 21: Proportion of incident cases attributable to reactivation for total population for the calibrated model. Figures show median model estimates (lines) and model uncertainty (shaded area).


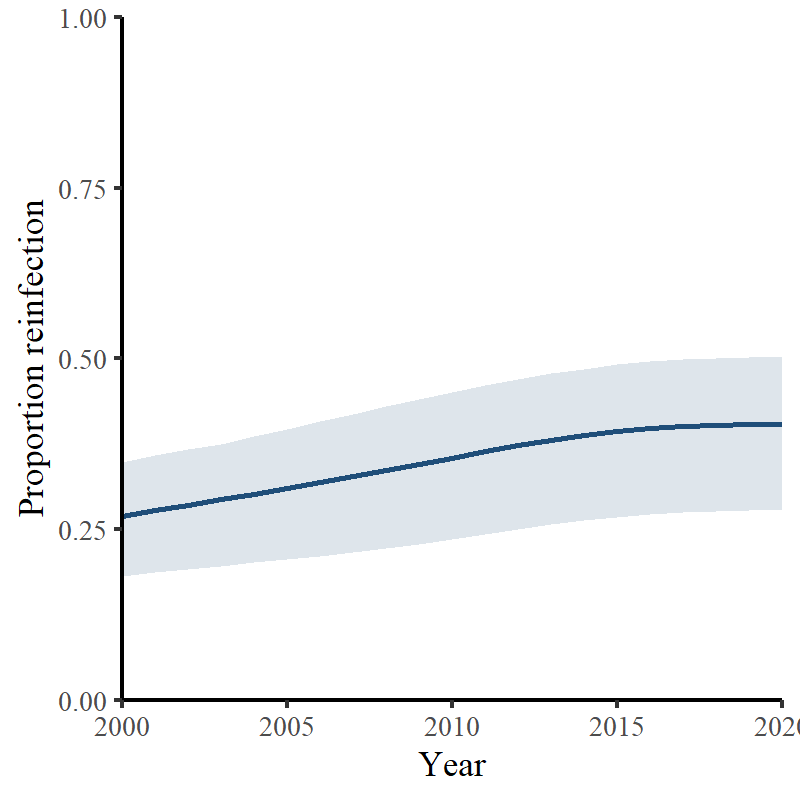

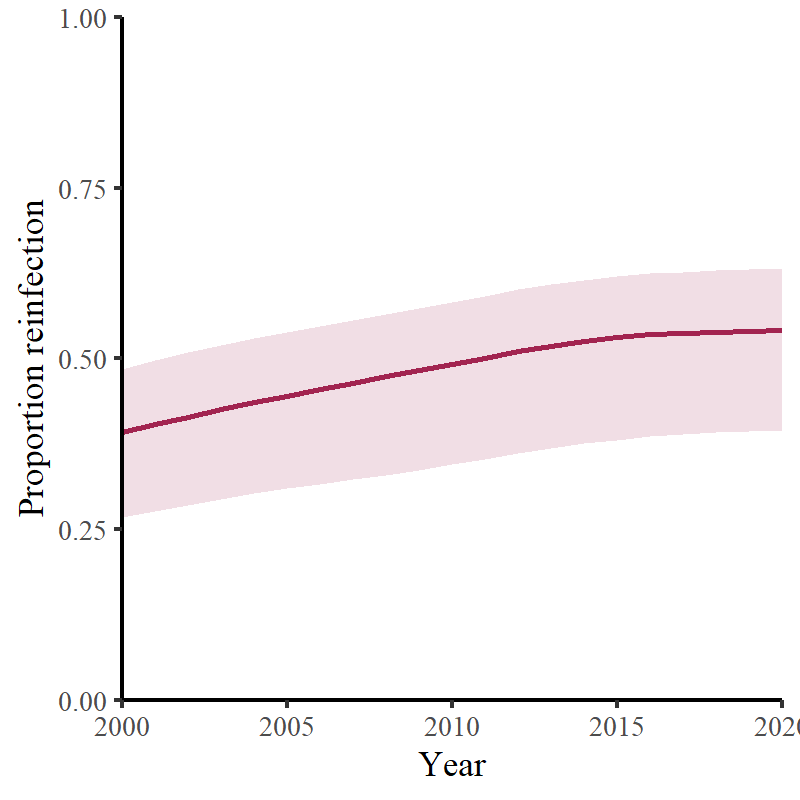

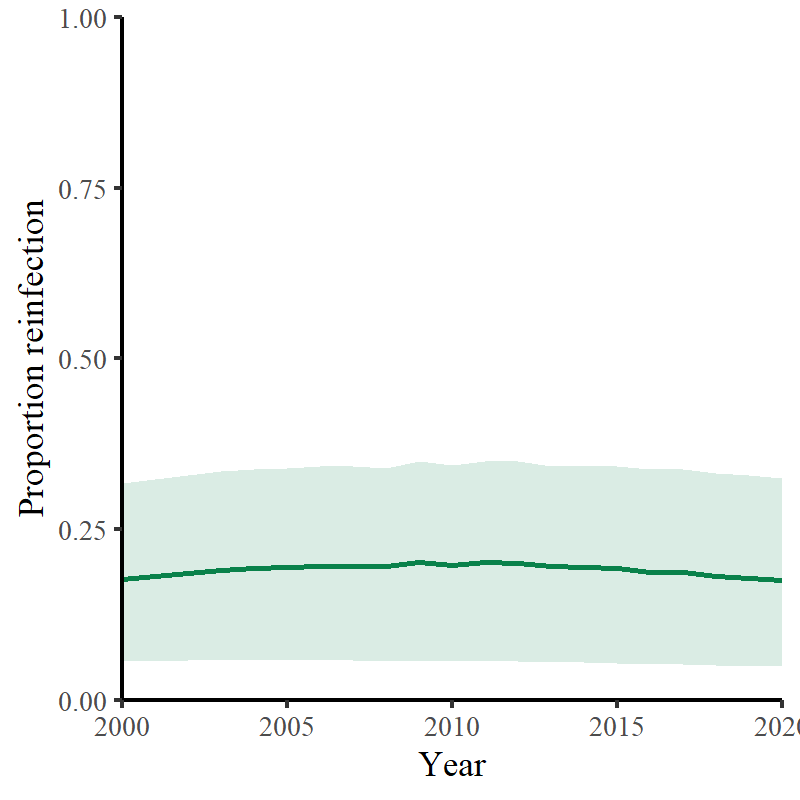


Figure 22: Proportion of incident cases attributable to reactivation for men (left in blue), women (centre in red), and children (right in green) for the calibrated model. Figures show median model estimates (lines) and model uncertainty (shaded area).

6.3.2 Mortality


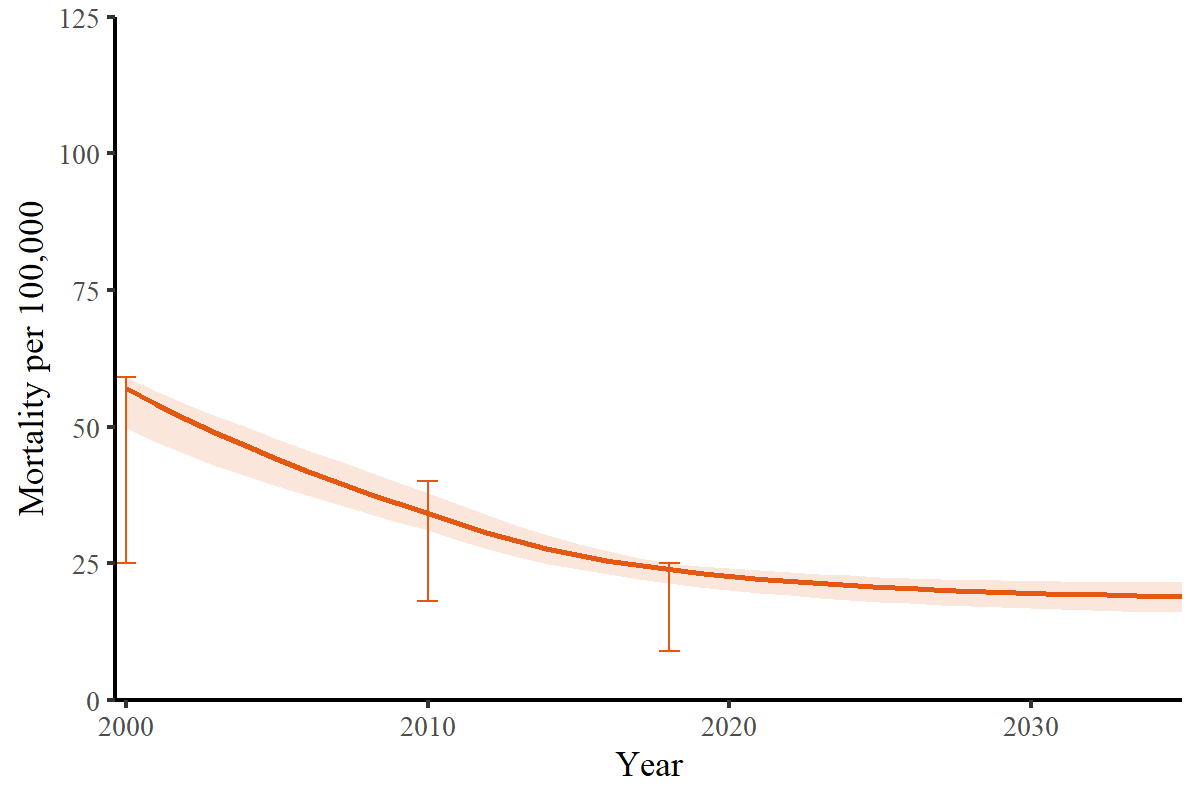


Figure 23: Mortality for total population for the calibrated model. Figure shows median model estimates (line), model uncertainty (shaded area), and calibration targets (error bars).


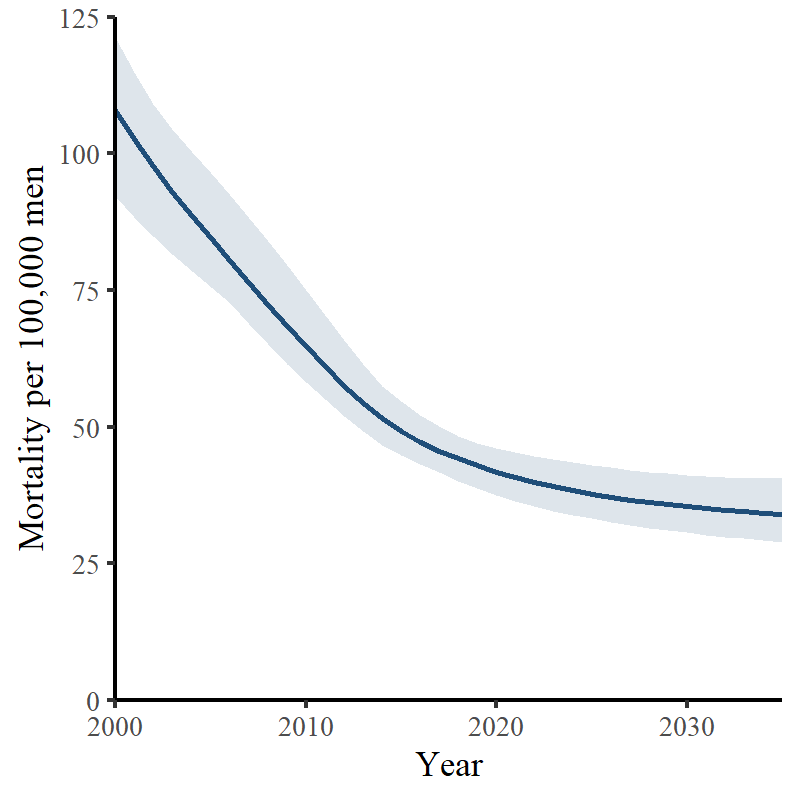

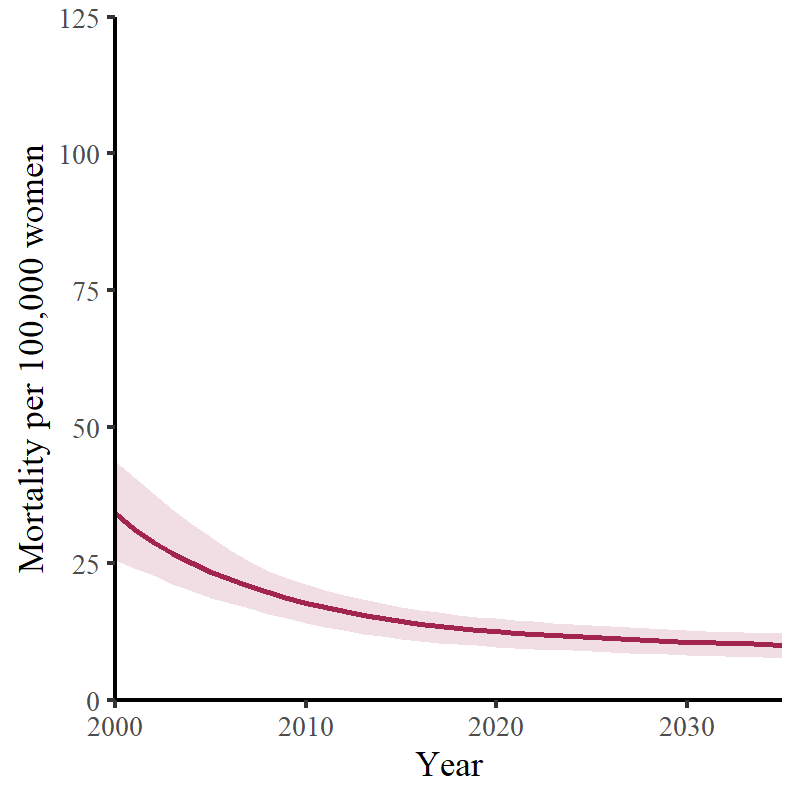

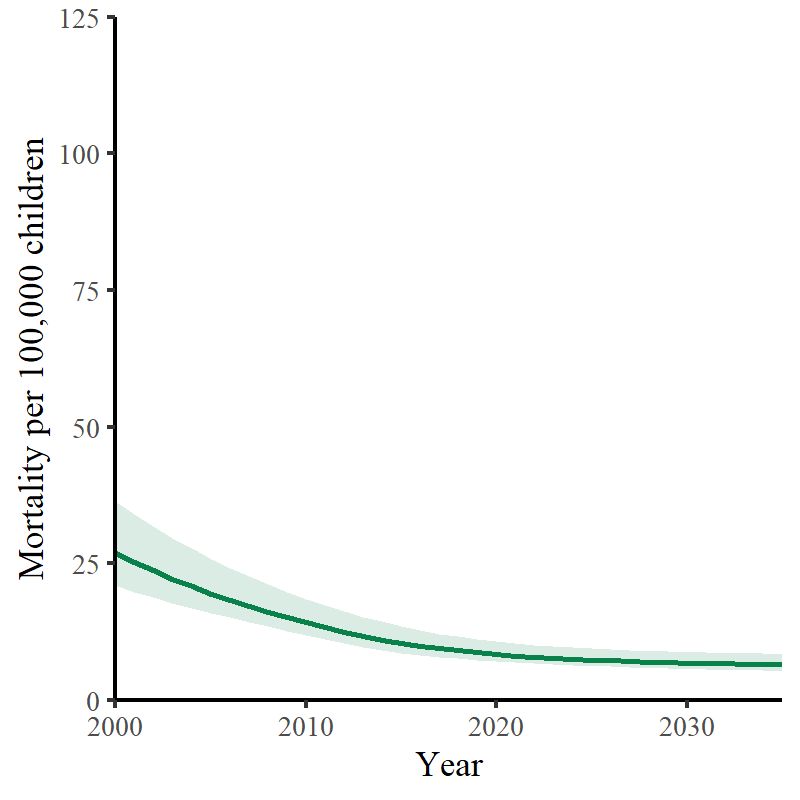


Figure 24: Mortality for men (left in blue), women (centre in red), and children (right in green) for the calibrated model. Figures show median model estimates (line) and model uncertainty (shaded area).


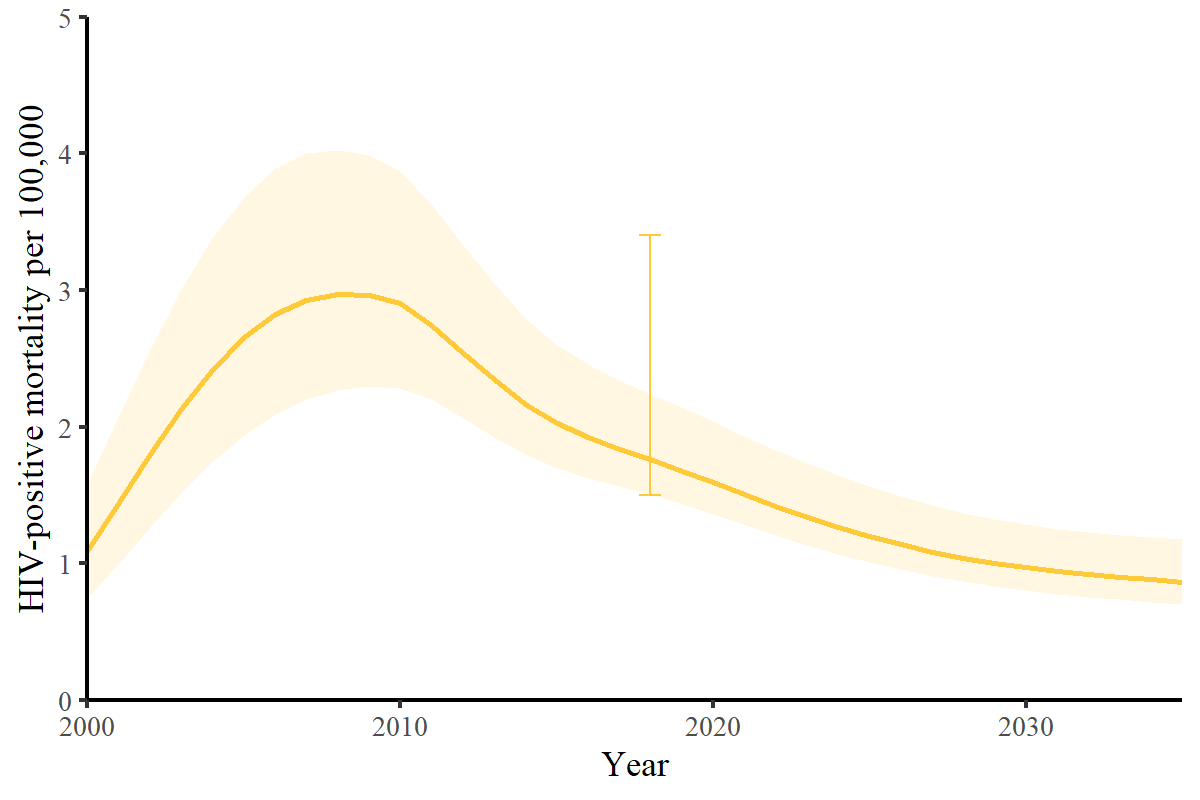


Figure 25: HIV-positive TB mortality for the calibrated model. Figure shows median model estimates (line), model uncertainty (shaded area), and calibration targets (error bars).

6.3.3 Case notification rates


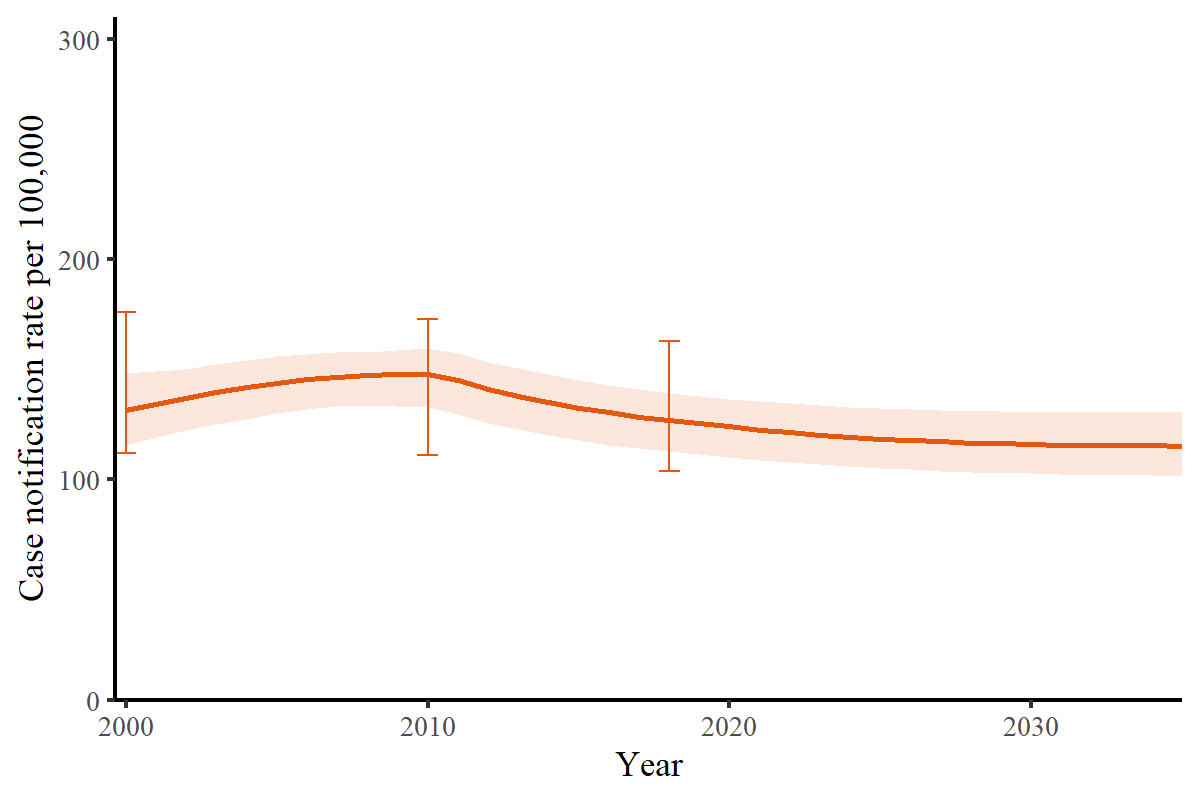


Figure 26: Case notification rate for total population for the calibrated model. Figure shows median model estimates (line), model uncertainty (shaded area), and calibration targets (error bars).


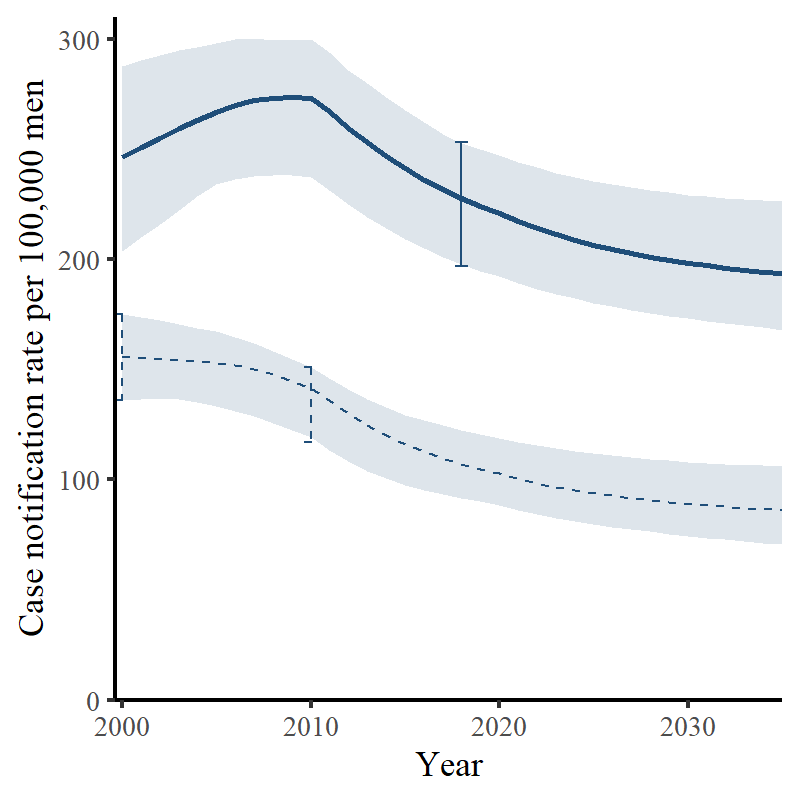

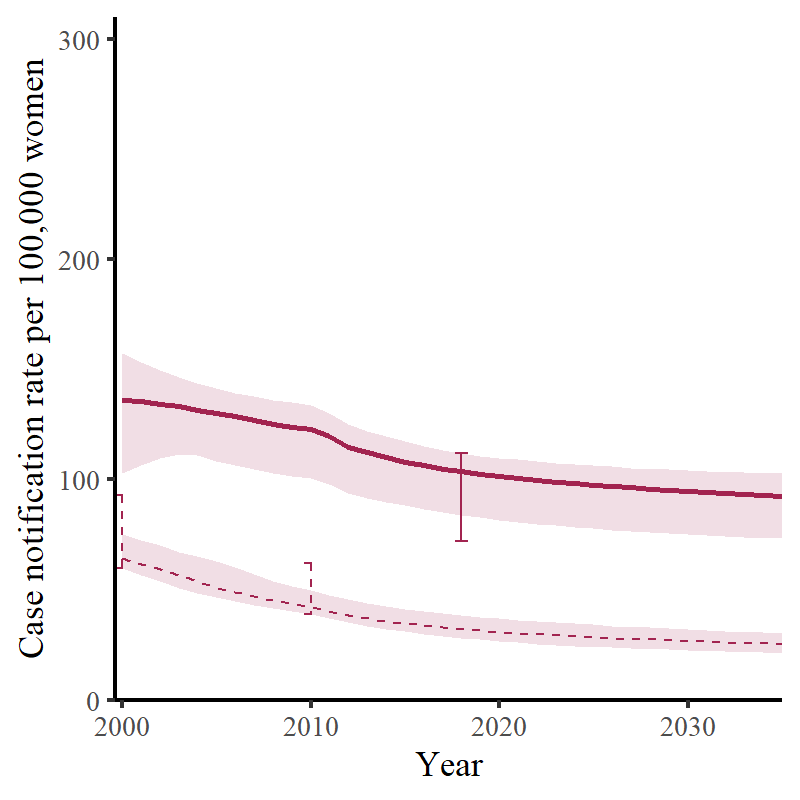

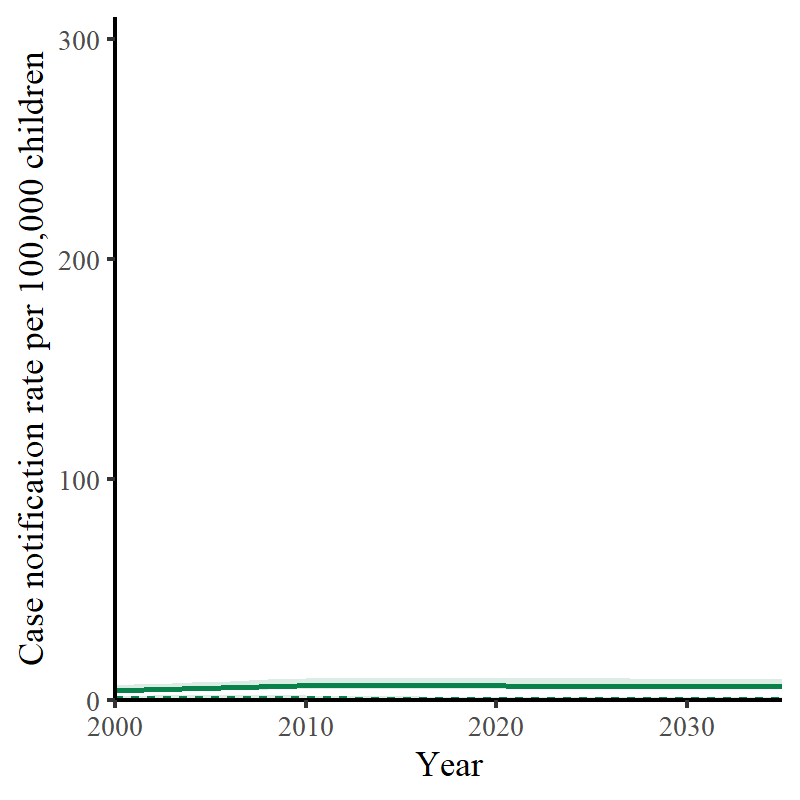


Figure 27: Case notification rate (smear-positive in dashed line, total in solid line) for men (left in blue), women (centre in red), and children (right in green) for the calibrated model. Figures show median model estimates (line), model uncertainty (shaded area), and calibration targets (error bars).


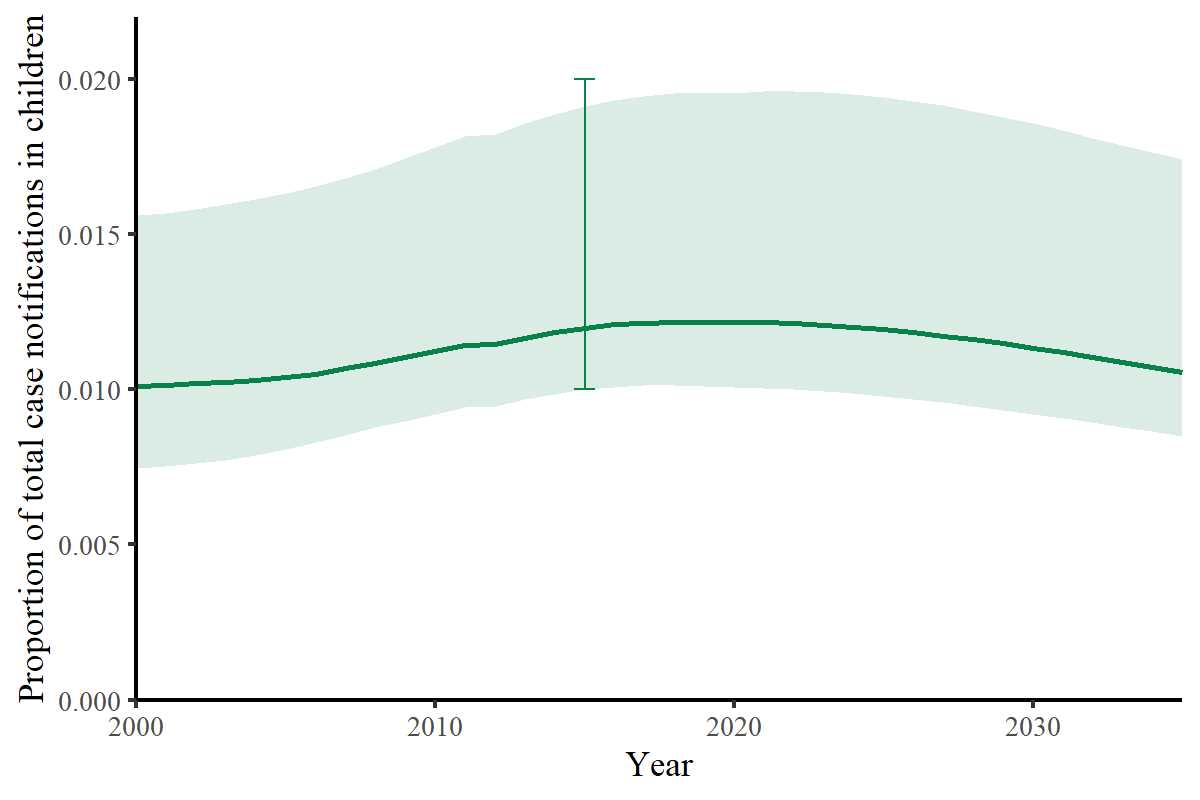


Figure 28: Proportion of total case notifications in children for the calibrated model. Figure shows median model estimates (line), model uncertainty (shaded area), and calibration targets (error bars).


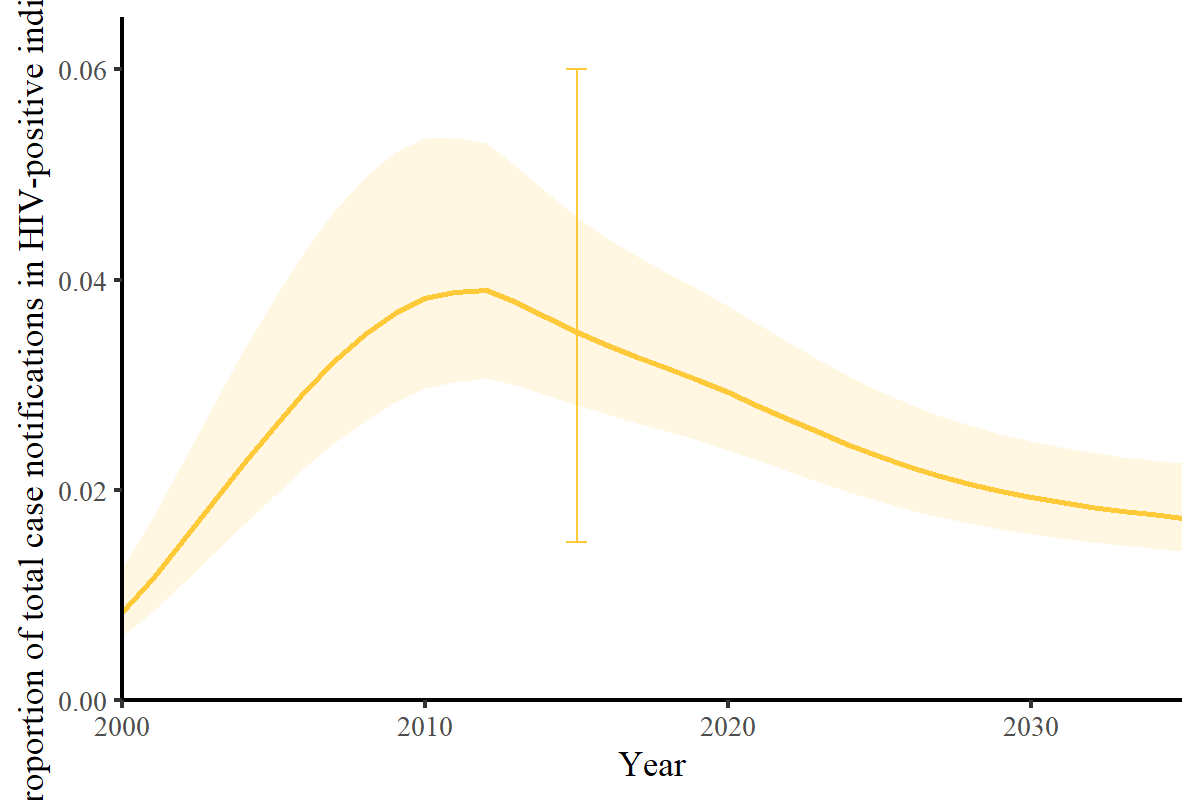


Proportion of total case notifications in
HIV-positive individuals

Figure 29: Proportion of total case notifications in HIV-positive individuals. Median model estimate (line), model uncertainty (shaded area) and calibration targets (error bars).

6.3.4 Prevalence


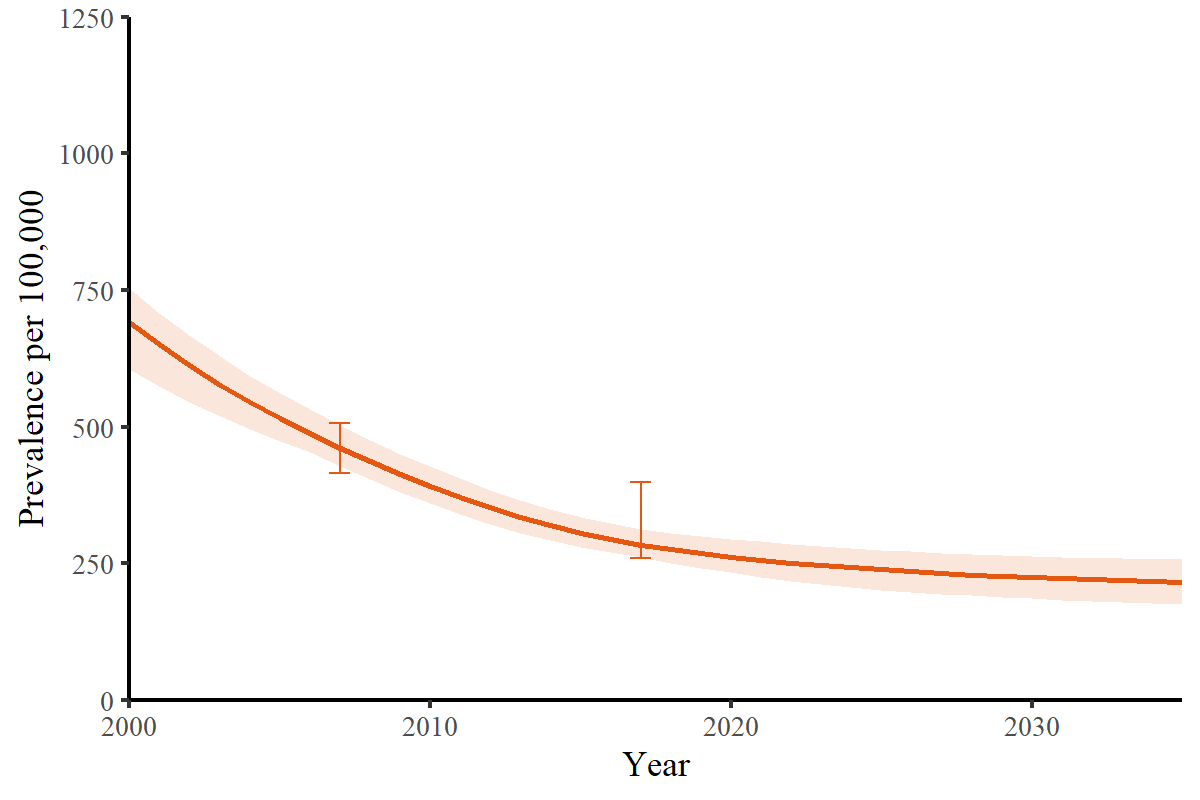


Figure 30: Prevalence for total population for the calibrated model. Figure shows median model estimates (line), model uncertainty (shaded area), and calibration targets (error bars).


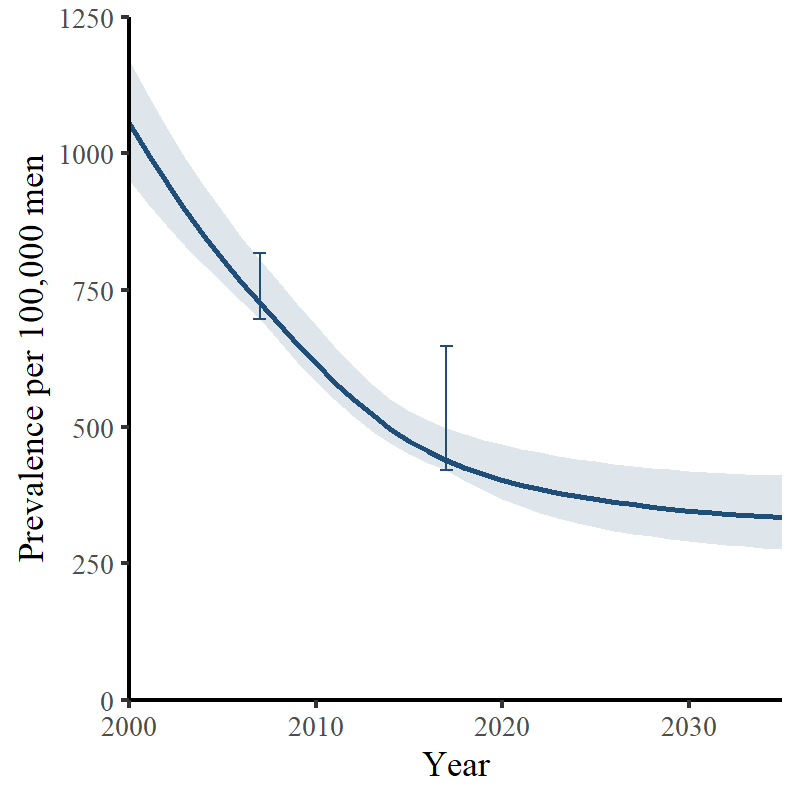

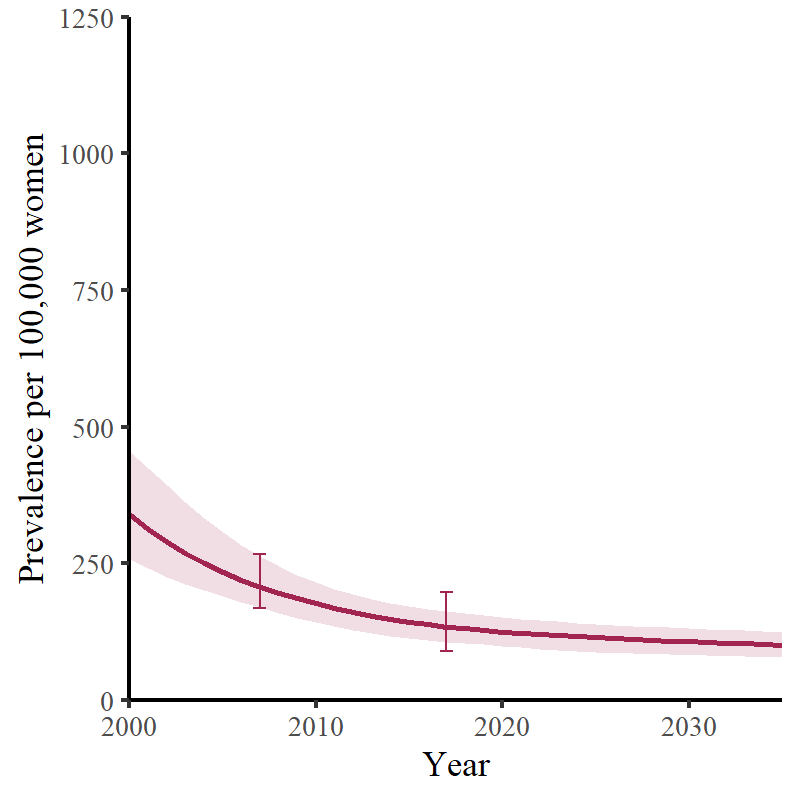

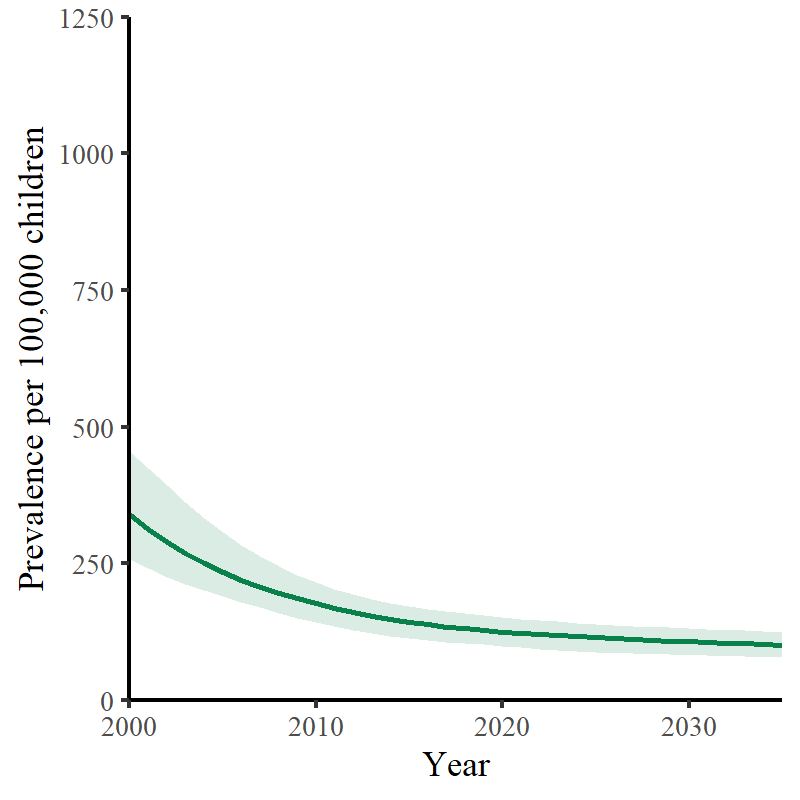


Figure 31: Prevalence for men (left in blue), women (centre in red), and children (right in green) for the calibrated model. Figures show median model estimates (line), model uncertainty (shaded area), and calibration targets (error bars).


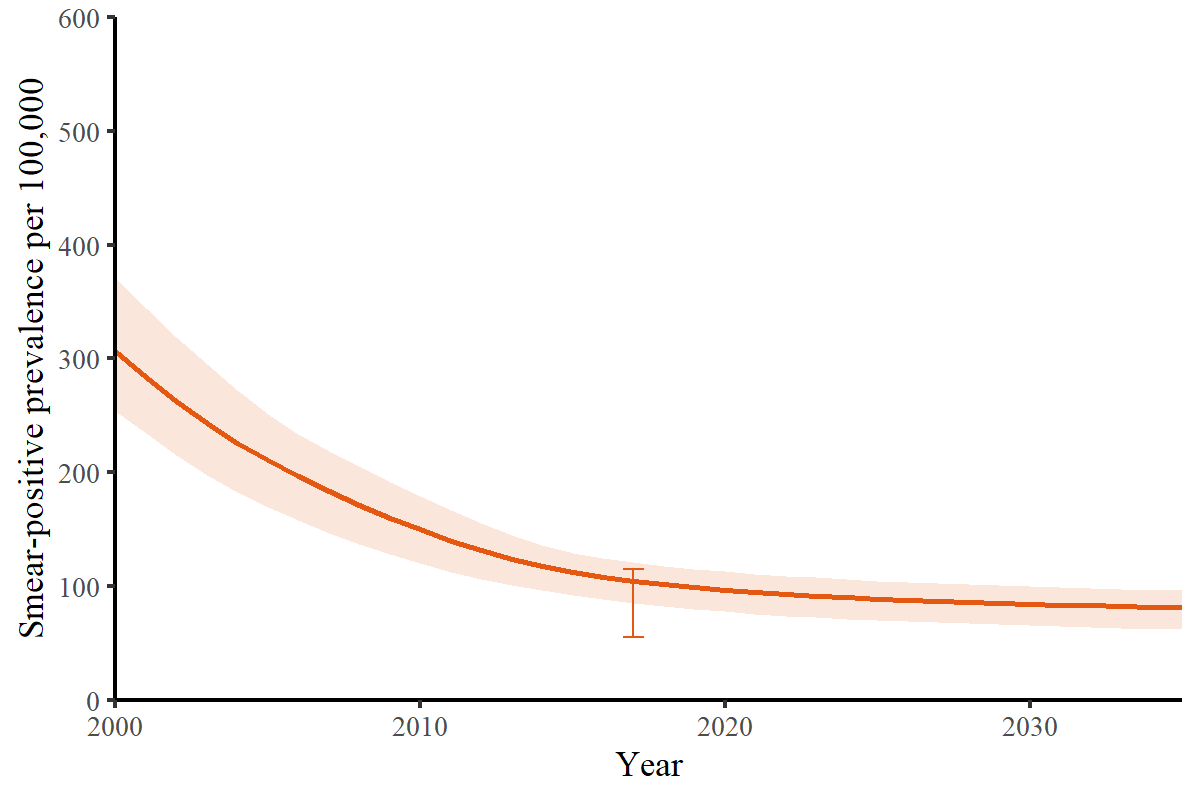


Figure 32: Smear-positive prevalence for total population for the calibrated model. Figure shows median model estimates (line), model uncertainty (shaded area), and calibration targets (error bars).


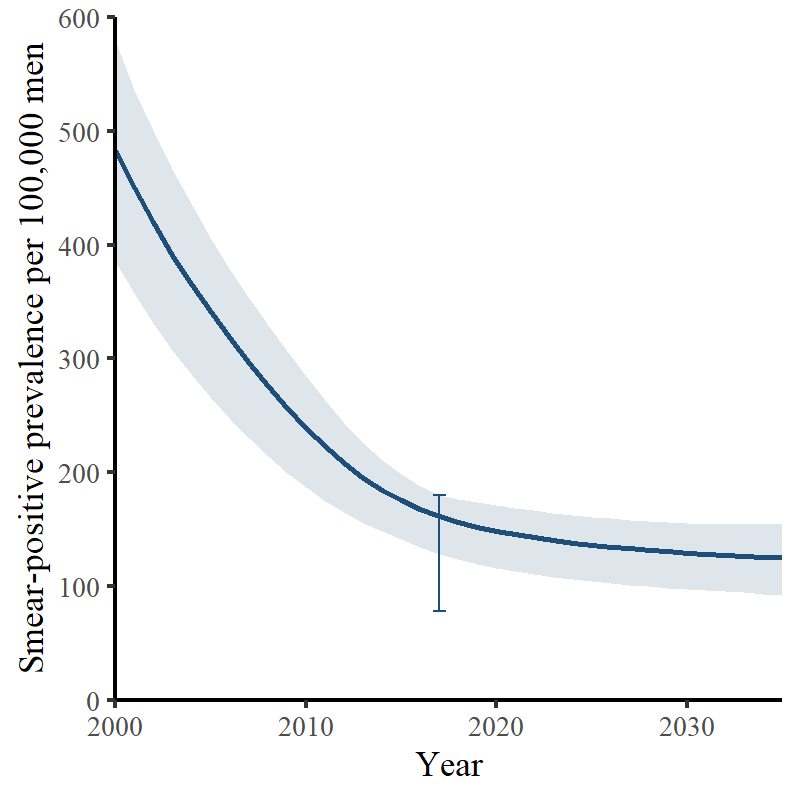

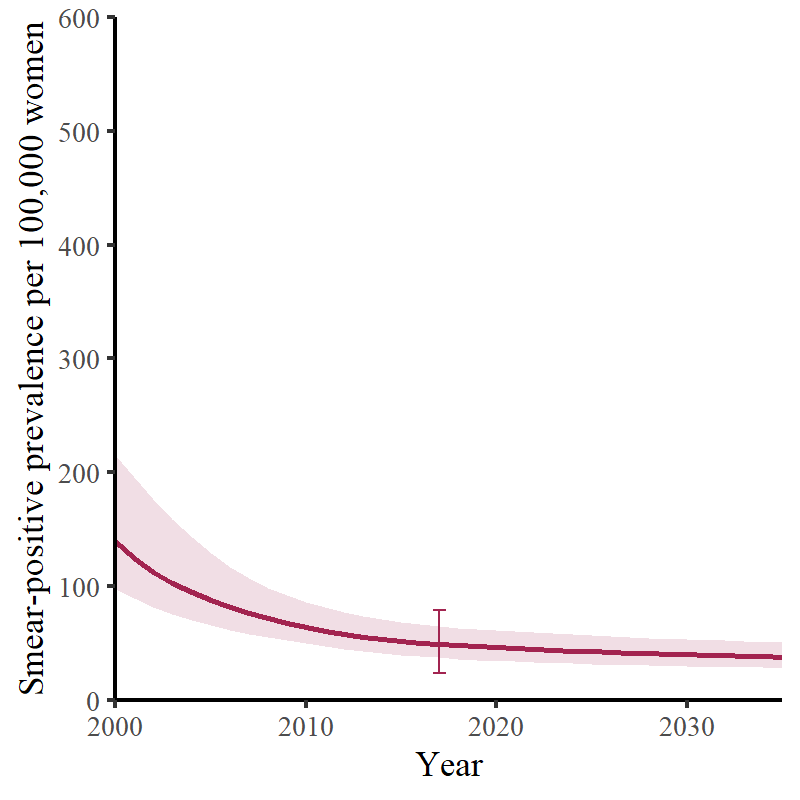

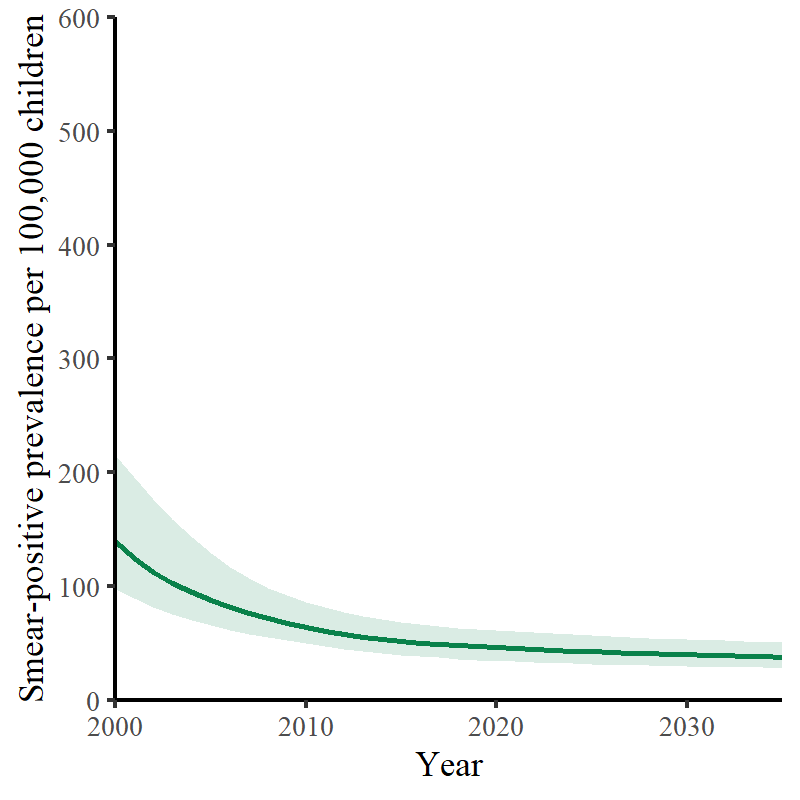


Figure 33: Smear-positive prevalence for men (left in blue), women (centre in red), and children (right in green) for the calibrated model. Figures show median model estimates (line), model uncertainty (shaded area), and calibration targets (error bars).

6.3.5 Male-to-female ratios


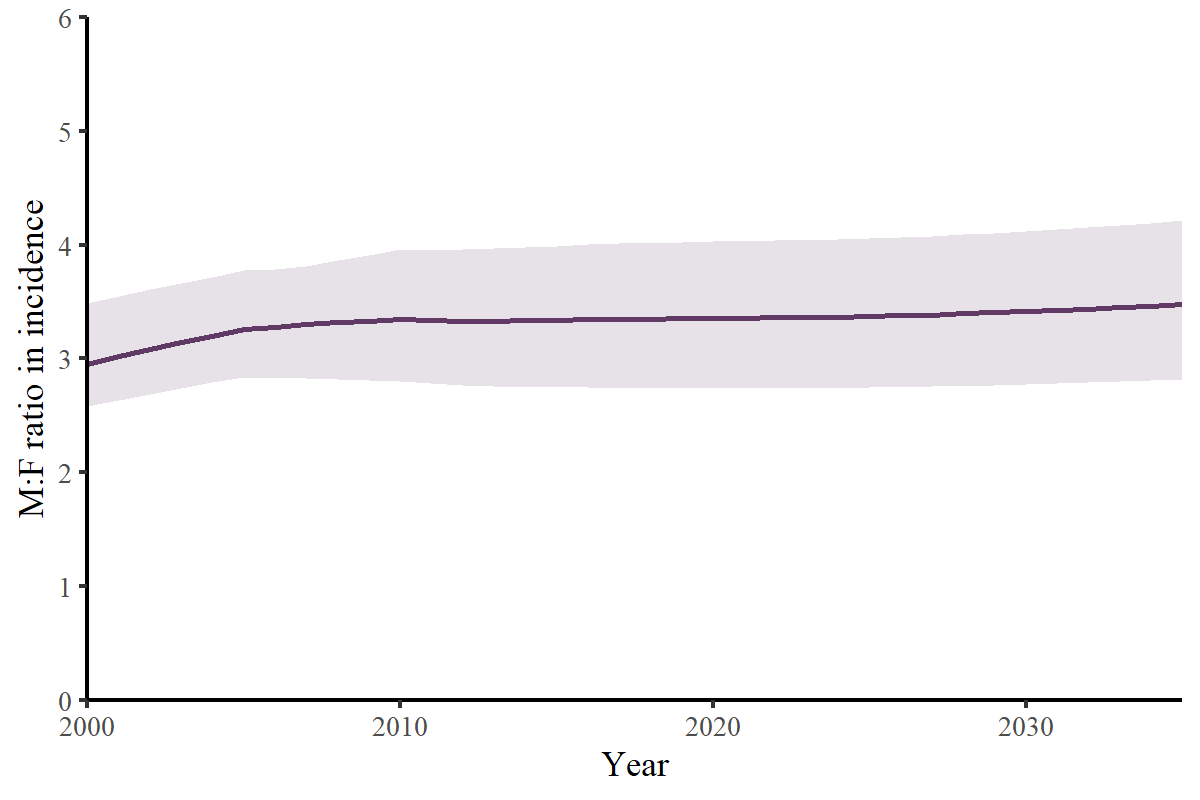


Figure 34: Male-to-female ratio in adult incidence for the calibrated model. Figure shows median model estimates (line) and model uncertainty (shaded area).


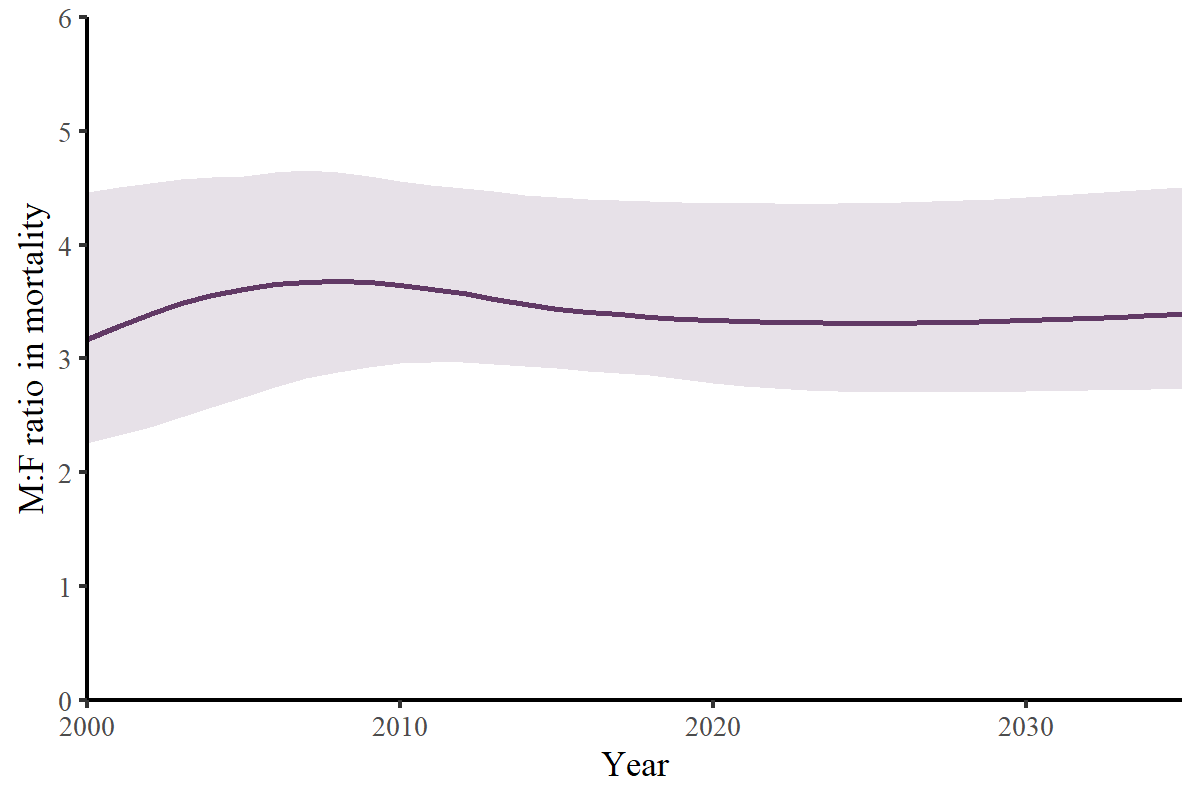


Figure 35: Male-to-female ratio in adult mortality for the calibrated model. Figure shows median model estimates (line) and model uncertainty (shaded area).


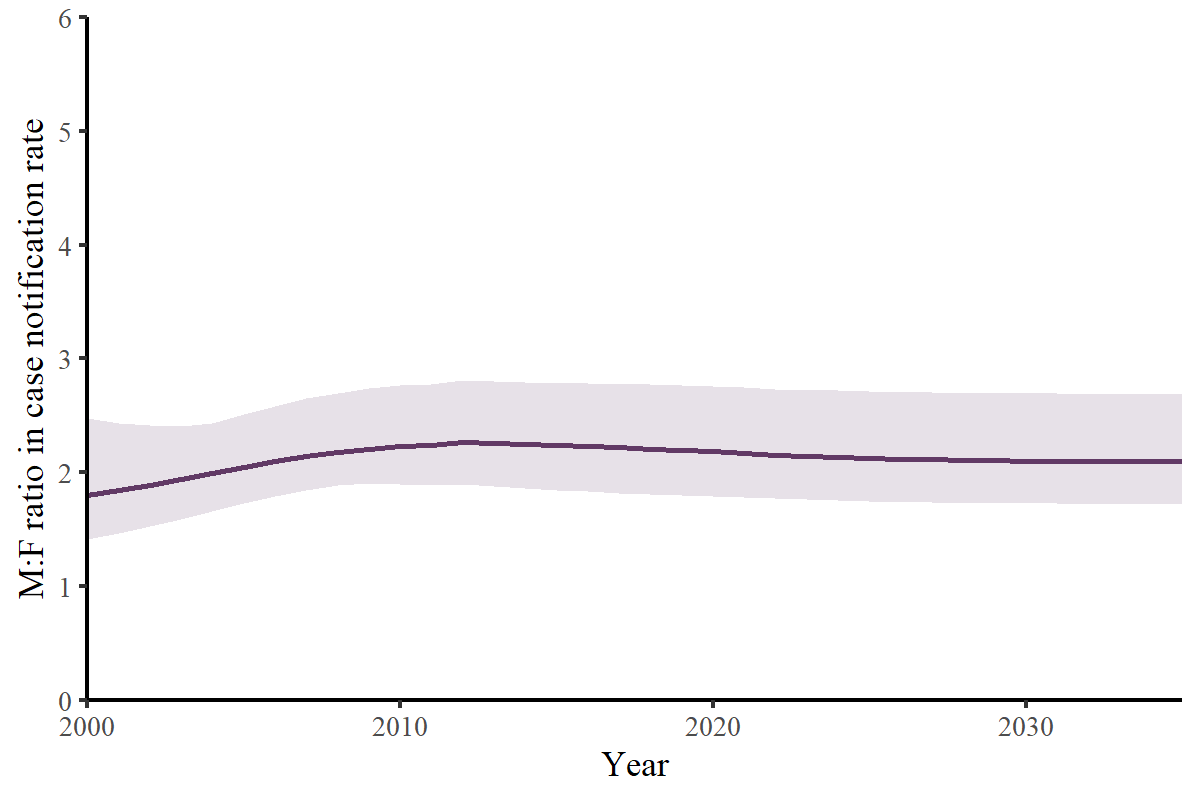

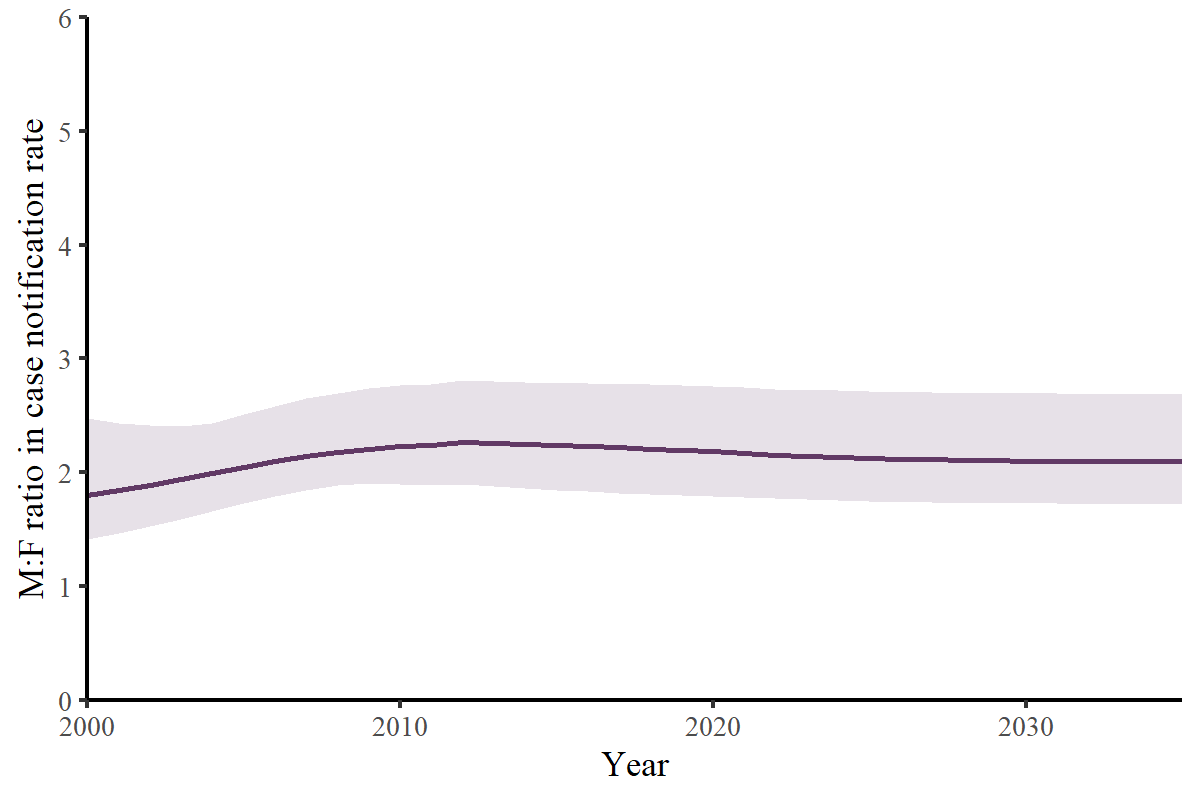


Figure 36: Male-to-female ratio in adult case notification rate (total on left, smear-positive on right) for the calibrated model. Figure shows median model estimates (line) and model uncertainty (shaded area).


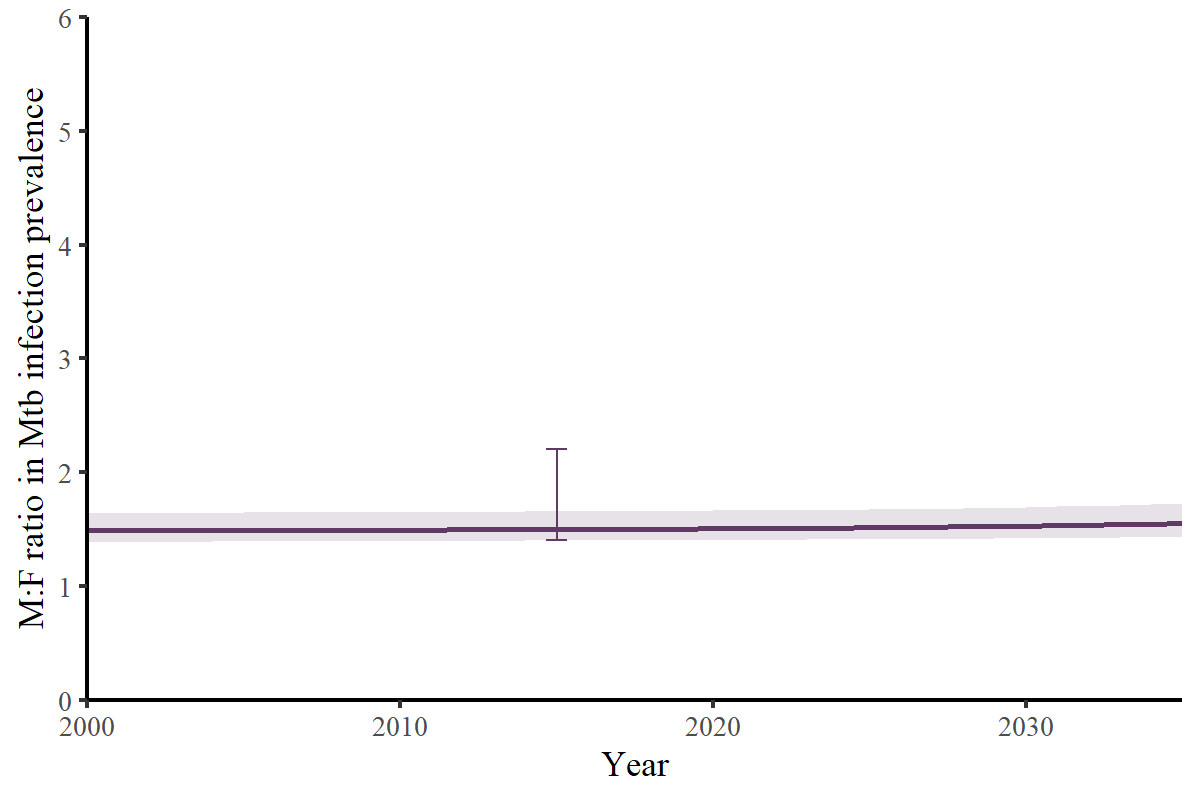


Figure 37: Male-to-female ratio in adult *Mtb* infection prevalence for the calibrated model. Figure shows median model estimates (line), model uncertainty (shaded area), and calibration targets (error bars).


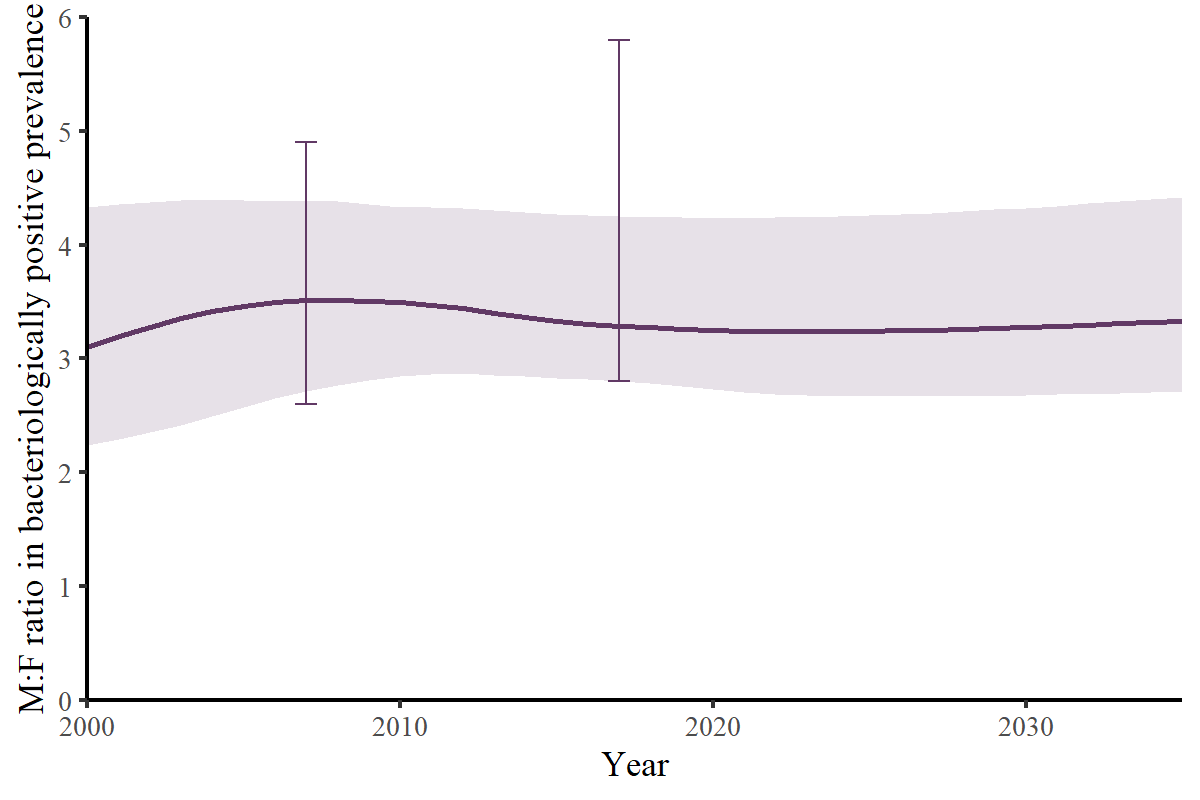

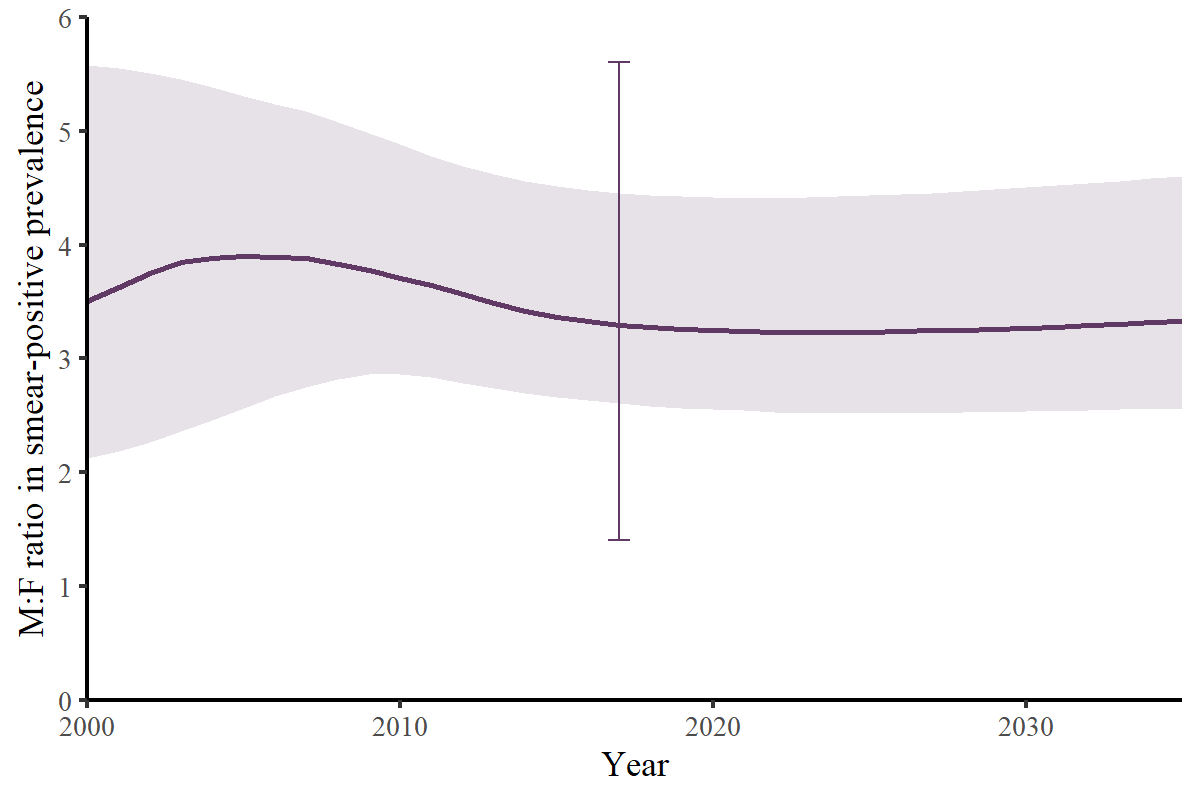


Figure 38: Male-to-female ratio in adult *Mtb* infection prevalence (bacteriologically positive on left, smear-positive on right) for the calibrated model. Figure shows median model estimates (line), model uncertainty (shaded area), and calibration targets (error bars).

6.3.6 MDR TB


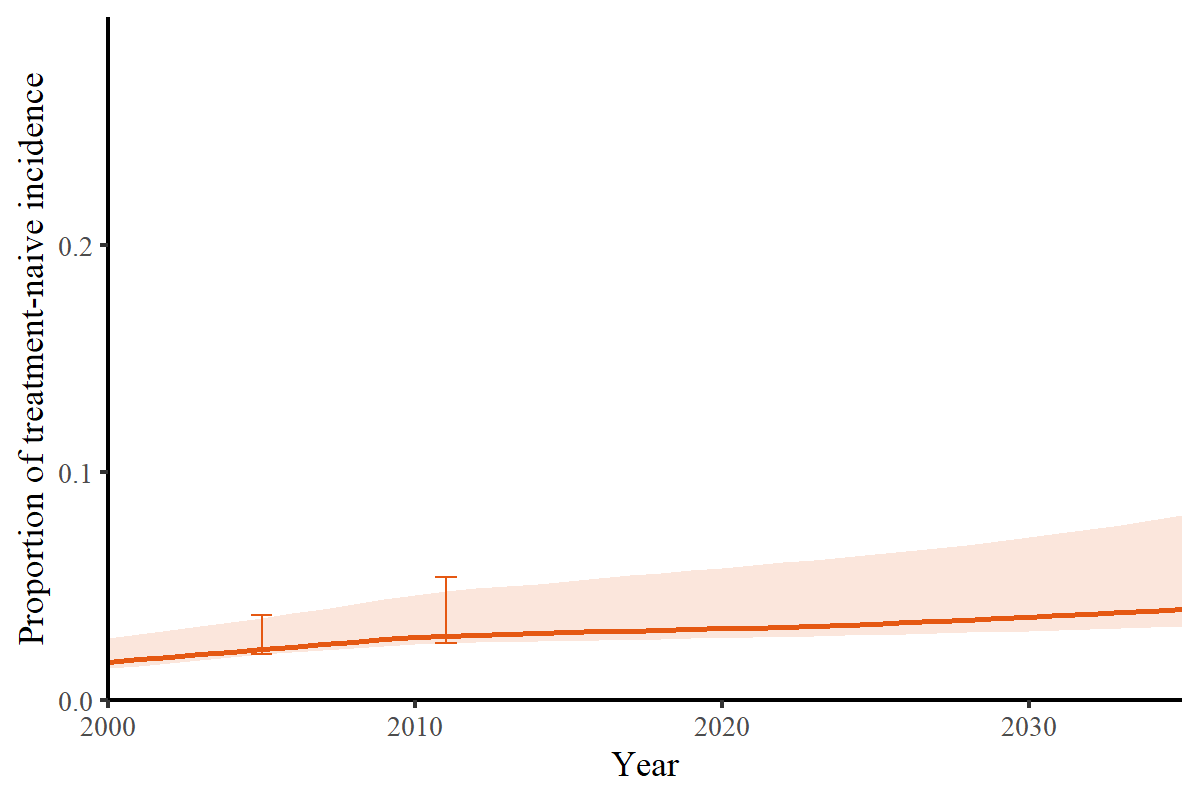

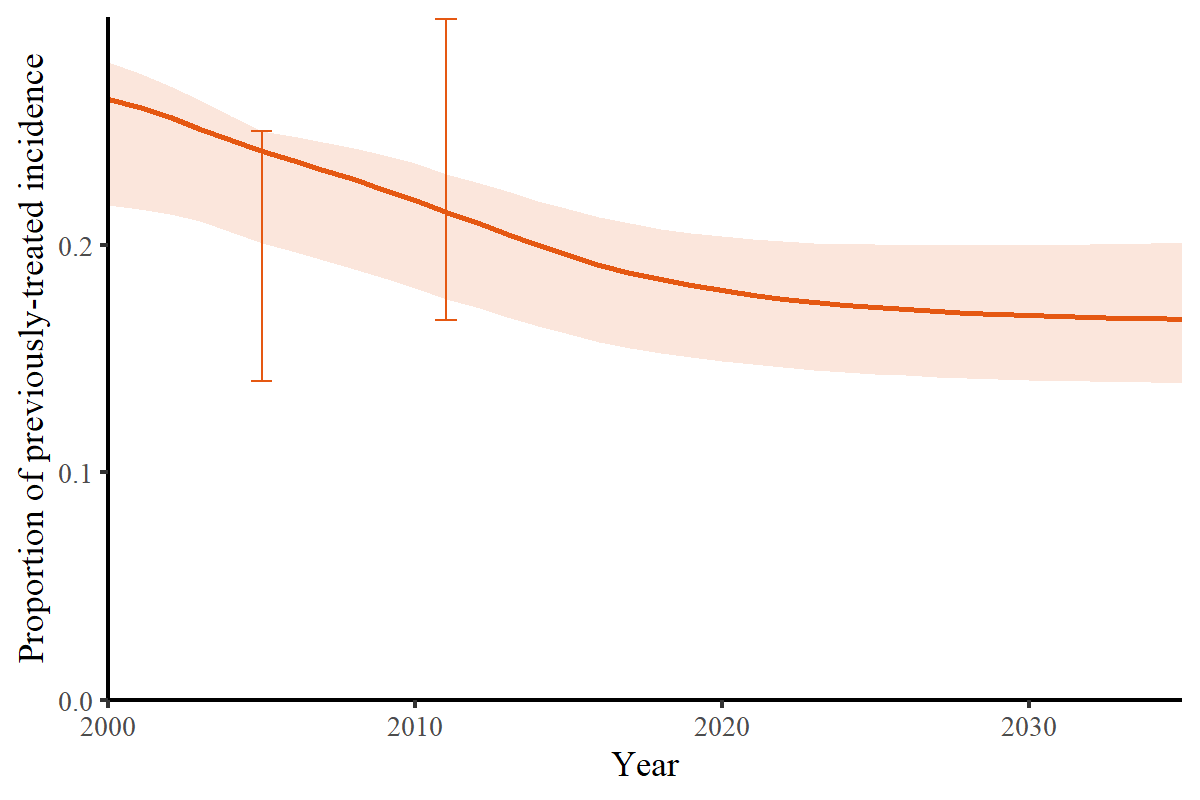


Figure 39: Proportion of MDR TB in incident TB (treatment naïve on left, previously-treated on right) for the calibrated model. Figure shows median model estimates (line), model uncertainty (shaded area), and calibration targets (error bars).

7 References

1. Houben R, Lalli M, Sumner T, Hamilton M, Pedrazzoli D, Bonsu F, et al. TIME Impact–a new user-friendly tuberculosis (TB) model to inform TB policy decisions. BMC Medicine. 2016;14:56. doi: 10.1186/s12916-016-0608-4.

2. Stover J, McKinnon R, Winfrey B. Spectrum: a model platform for linking maternal and child survival interventions with AIDS, family planning and demographic projections. International journal of epidemiology. 2010;39(suppl_1):i7-i10.

3. United Nations DoEaSA, Population Division;. World Population Prospects 2019, custom data acquired via website. 2019.

4. United Nations Department of Economic and Social Affairs. World Population Prospects New York: Population Division of the Department of Economic and Social Affairs of the United Nations Secretariat; 2017 [cited 2018 21 June]. Available from: https://esa.un.org/unpd/wpp/DVD/Files/1_Indicators%20(Standard)/EXCEL_FILES/2_Fertility/WPP2017_FERT_F02_SEX_RATIO_AT_BIRTH.xlsx.

5. Behr M, Warren S, Salamon H, Hopewell P, De Leon AP, Daley C, et al. Transmission of Mycobacterium tuberculosis from patients smear-negative for acid-fast bacilli. Lancet. 1999;353(9151):444-9.

6. Dowdy DW, Chaisson RE. The persistence of tuberculosis in the age of DOTS: reassessing the effect of case detection. Bulletin of the World Health Organization. 2009;87(4):296-304.

7. Menzies NA, Cohen T, Lin H-H, Murray M, Salomon JA. Population health impact and cost-effectiveness of tuberculosis diagnosis with Xpert MTB/RIF: a dynamic simulation and economic evaluation. PLoS Med. 2012;9(11):e1001347. doi: 10.1371/journal.pmed.1001347.

8. Dye C, Williams BG. Eliminating human tuberculosis in the twenty-first century. Journal of the Royal Society Interface. 2008;5(23):653-62.

9. Abu-Raddad LJ, Sabatelli L, Achterberg JT, Sugimoto JD, Longini IM, Dye C, et al. Epidemiological benefits of more-effective tuberculosis vaccines, drugs, and diagnostics. Proceedings of the National Academy of Sciences. 2009;106(33):13980-5.

10. Knight GM, Griffiths UK, Sumner T, Laurence YV, Gheorghe A, Vassall A, et al. Impact and cost-effectiveness of new tuberculosis vaccines in low-and middle-income countries. Proceedings of the National Academy of Sciences. 2014;111(43):15520-5.

11. Dye C, Williams BG. Criteria for the control of drug-resistant tuberculosis. Proceedings of the National Academy of Sciences. 2000;97(14):8180-5.

12. Horby P, Thai PQ, Hens N, Yen NTT, Thoang DD, Linh NM, et al. Social contact patterns in Vietnam and implications for the control of infectious diseases. PLoS One. 2011;6(2):e16965.

13. Baguelin M, Flasche S, Camacho A, Demiris N, Miller E, Edmunds WJ. Assessing optimal target populations for influenza vaccination programmes: an evidence synthesis and modelling study. PLoS medicine. 2013;10(10):e1001527.

14. Global Burden of Disease Collaborative Network. Global Burden of Disease Study 2015 (GBD 2015) Smoking Prevalence 1980-2015. Seattle, United States: Institute for Health Metrics and Evaluation (IHME), 2017.

15. United Nations Department of Economic and Social Affairs Population Division. World Population Prospects 2019, Online Edition. Rev. 1. 2019.

16. World Health Organization Global Health Observatory Data Repository. Prevalence of current tobacco use (crude-adjusted rate) 2020. Available from: https://apps.who.int/gho/data/view.sdg.3-a-data-ctry?lang=en.

17. Lin H-H, Ezzati M, Murray M. Tobacco smoke, indoor air pollution and tuberculosis: a systematic review and meta-analysis. PLoS Medicine. 2007;4(1):e20.

18. Basu S, Stuckler D, Bitton A, Glantz SA. Projected effects of tobacco smoking on worldwide tuberculosis control: mathematical modelling analysis. BMJ. 2011;343:d5506.

19. Bates MN, Khalakdina A, Pai M, Chang L, Lessa F, Smith KR. Risk of tuberculosis from exposure to tobacco smoke: a systematic review and meta-analysis. Archives of Internal Medicine. 2007;167(4):335-42.

20. Neyrolles O, Quintana-Murci L. Sexual inequality in tuberculosis. PLoS Medicine. 2009;6(12):e1000199.

21. Nhamoyebonde S, Leslie A. Biological differences between the sexes and susceptibility to tuberculosis. Journal of Infectious Diseases. 2014;209(suppl 3):S100–S6.

22. Sutherland I, Švandová E, Radhakrishna S. The development of clinical tuberculosis following infection with tubercle bacilli: 1. A theoretical model for the development of clinical tuberculosis following infection, linking from data on the risk of tuberculous infection and the incidence of clinical tuberculosis in the Netherlands. Tubercle. 1982;63(4):255-68.

23. Cohen T, Lipsitch M, Walensky RP, Murray M. Beneficial and perverse effects of isoniazid preventive therapy for latent tuberculosis infection in HIV–tuberculosis coinfected populations. Proceedings of the National Academy of Sciences. 2006;103(18):7042-7.

24. Lawn SD, Kranzer K, Wood R. Antiretroviral therapy for control of the HIV-associated tuberculosis epidemic in resource-limited settings. Clinics in chest medicine. 2009;30(4):685-99.

25. Dye C, Garnett GP, Sleeman K, Williams BG. Prospects for worldwide tuberculosis control under the WHO DOTS strategy. Lancet. 1998;352(9144):1886–91.

26. Eamranond P, Jaramillo E. Tuberculosis in children: reassessing the need for improved diagnosis in global control strategies. The International Journal of Tuberculosis and Lung Disease. 2001;5(7):594-603.

27. Global Burden of Disease Collaborative Network. Global Burden of Disease Study 2016 (GBD 2016) Alcohol Use Estimates 1990-2016. In: Institute for Health Metrics and Evaluation (IHME), editor. Seattle, WA, USA2018.

28. World Health Organization Global Health Observatory Data Repository. Total alcohol consumption per capita, female (liters of pure alcohol, projected estimates, female 15+ years of age) 2020. Available from: https://data.worldbank.org/indicator/SH.ALC.PCAP.FE.LI.

29. World Health Organization Global Health Observatory Data Repository. Total alcohol consumption per capita, male (liters of pure alcohol, projected estimates, male 15+ years of age) 2020. Available from: https://data.worldbank.org/indicator/SH.ALC.PCAP.MA.LI.

30. Rehm J, Samokhvalov AV, Neuman MG, Room R, Parry C, Lönnroth K, et al. The association between alcohol use, alcohol use disorders and tuberculosis (TB). A systematic review. BMC public health. 2009;9(1):450.

31. Imtiaz S, Shield KD, Roerecke M, Samokhvalov AV, Lönnroth K, Rehm J. Alcohol consumption as a risk factor for tuberculosis: meta-analyses and burden of disease. European Respiratory Journal. 2017;50(1):1700216.

32. Zwerling A, Behr MA, Verma A, Brewer TF, Menzies D, Pai M. The BCG World Atlas: a database of global BCG vaccination policies and practices. PLoS Med. 2011;8(3):e1001012.

33. World Health Organization. WHO-UNICEF estimates of BCG coverage 2020 [08 October 2020]. Available from: https://apps.who.int/immunization_monitoring/globalsummary/timeseries/tswucoveragebcg.html.

34. Widyono M, Sherris J. Sex-disaggregated immunization coverage data: Input from key stakeholders. Washington, DC, USA: PATH, 2010.

35. Dodd PJ, Gardiner E, Coghlan R, Seddon JA. Burden of childhood tuberculosis in 22 high-burden countries: a mathematical modelling study. The lancet global health. 2014;2(8):e453-e9.

36. Sonnenberg P, Glynn JR, Fielding K, Murray J, Godfrey-Faussett P, Shearer S. How soon after infection with HIV does the risk of tuberculosis start to increase? A retrospective cohort study in South African gold miners. Journal of Infectious Diseases. 2005;191(2):150-8.

37. Ferebee S. Controlled chemoprophylaxis trial in tuberculosis, A general review. Adv Tuberc Res. 1970;17:28-106.

38. Gilks CF, Godfrey-Faussett P, Batchelor BI, Ojoo JC, Ojoo SJ, Brindle RJ, et al. Recent transmission of tuberculosis in a cohort of HIV-1-infected female sex workers in Nairobi, Kenya. Aids. 1997;11(7):911-8.

39. Corbett E, Watt C, Walker N, Maher D, Williams B, Raviglione M, et al. The Growing Burden of Tuberculosis Global Trends and Interactions With the HIV Epidemic. Arch Intern Med. 2003;163(1009-1021).

40. Praygod G, Todd J, McDermid J. Early childhood tuberculosis in northwestern Tanzania. Int J Tuberc Lung Dis. 2012;16(11):1455-60.

41. Horton KC, Sumner T, Houben RM, Corbett EL, White RG. A Bayesian approach to understanding sex differences in tuberculosis disease burden. Am J Epidemiol. 2018;187(11):2431-8.

42. Nguyen HV, Tiemersma EW, Nguyen HB, Cobelens FG, Finlay A, Glaziou P, et al. The second national tuberculosis prevalence survey in Vietnam. PLoS One. 2020;15(4):e0232142.

43. TIME: TB Impact Model and Estimates for Viet Nam. 2017.

44. Espinal MA, Kim SJ, Suarez PG, Kam KM, Khomenko AG, Migliori GB, et al. Standard short-course chemotherapy for drug-resistant tuberculosis: treatment outcomes in 6 countries. Jama. 2000;283(19):2537-45.

45. Lew W, Pai M, Oxlade O, Martin D, Menzies D. Initial drug resistance and tuberculosis treatment outcomes: systematic review and meta-analysis. Annals of internal medicine. 2008;149(2):123-34.

46. R Core Team. A language and environment for statistical computing. Vienna, Austria: R Foundation for Statistical Computing, 2015.

47. Press W, Teukolsky S, Vetterling W, Flannery B. Numerical Recipes: The Art of Scientific Computing. Cambridge, UK: Cambridge University Press; 2007.

48. Harris RC, Sumner T, Knight GM, Evans T, Cardenas V, Chen C, et al. Age-targeted tuberculosis vaccination in China and implications for vaccine development: a modelling study. The Lancet Global Health. 2019;7(2):e209-e18.

49. Jabot F, Faure T, Dumoulin N. Easy ABC: performing efficient approximate B ayesian computation sampling schemes using R. Methods in Ecology and Evolution. 2013;4(7):684-7.

50. World Health Organization. WHO TB burden estimates. 2020 ed. Geneva, Switzerland: World Health Organization; 2020.

51. World Health Organization. Case notifications. 2020 ed. Geneva, Switzerland: World Health Organization; 2020.

52. Hoa NB, Cobelens FG, Sy DN, Nhung NV, Borgdorff MW, Tiemersma EW. Diagnosis and treatment of tuberculosis in the private sector, Vietnam. Emerging infectious diseases. 2011;17(3):562.

53. Glaziou P. Revised estimates for first national prevalence survey. In: Horton KC, editor. 2019.

54. Marks G, Nhung N, Nguyen T, Hoa N, Khoa T, Son N, et al. Prevalence of latent tuberculous infection among adults in the general population of Ca Mau, Viet Nam. The International Journal of Tuberculosis and Lung Disease. 2018;22(3):246-51.

1. States S: susceptible; L: latent infection; I: smear-positive TB; N: smear-negative TB.

   Parameters λ: force of infection; x: proportion protection due to previous infection against progression to active disease following reinfection; α: proportion of new infections developing primary disease; ν: reactivation rate; σ: proportion of cases developing smear-positive disease; θ: rate of conversion from smear-negative to smear-positive disease; r: rate of TB self-cure; d: relative detection of smear-negative TB; γ: rate of access to TB care; Se: net sensitivity of diagnostic algorithm; Sp: net specificity of diagnostic algorithm; ψ: DST coverage; η: proportion of diagnosed individuals who are linked to treatment; τ: proportion of individuals linked to care who successfully complete treatment; ξ: rate of acquisition of resistance during treatment; ι: proportion of non-progressing superinfections.

   Subscripts X: treatment-naïve; P: previously treated; S: drug-susceptible; R: MDR; g: sex; a: age; h: HIV status; k: ART duration; t: time-step. [↑](#footnote-ref-2)
2. States S: susceptible; L: latent infection; I: smear-positive TB; N: smear-negative TB.

   Parameters λ: force of infection; x: proportion protection due to previous infection against progression to active disease following reinfection; α: proportion of new infections developing primary disease; ν: reactivation rate; σ: proportion of ca2ses developing smear-positive disease; θ: rate of conversion from smear-negative to smear-positive disease; r: rate of TB self-cure; d: relative detection of smear-negative TB; γ: rate of access to TB care; Se: net sensitivity of diagnostic algorithm; Sp: net specificity of diagnostic algorithm; ψ: DST coverage; η: proportion of diagnosed individuals who are linked to treatment; τ: proportion of individuals linked to care who successfully complete treatment; ξ: rate of acquisition of resistance during treatment; ι: proportion of non-progressing superinfections.

   Subscripts X: treatment-naïve; P: previously treated; S: drug-susceptible; R: MDR; g: sex; a: age; h: HIV status; k: ART duration; t: time-step. [↑](#footnote-ref-3)
3. States S: susceptible; L: latent infection; I: smear-positive TB; N: smear-negative TB.

   Parameters λ: force of infection; x: proportion protection due to previous infection against progression to active disease following reinfection; α: proportion of new infections developing primary disease; ν: reactivation rate; σ: proportion of cases developing smear-positive disease; θ: rate of conversion from smear-negative to smear-positive disease; r: rate of TB self-cure; d: relative detection of smear-negative TB; γ: rate of access to TB care; Se: net sensitivity of diagnostic algorithm; Sp: net specificity of diagnostic algorithm; ψ: DST coverage; η: proportion of diagnosed individuals who are linked to treatment; τ: proportion of individuals linked to care who successfully complete treatment; ξ: rate of acquisition of resistance during treatment; ι: proportion of non-progressing superinfections.

   Subscripts X: treatment-naïve; P: previously treated; S: drug-susceptible; R: MDR; g: sex; a: age; h: HIV status; k: ART duration; t: time-step. [↑](#footnote-ref-4)
4. States S: susceptible; L: latent infection; I: smear-positive TB; N: smear-negative TB.

   Parameters λ: force of infection; x: proportion protection due to previous infection against progression to active disease following reinfection; α: proportion of new infections developing primary disease; ν: reactivation rate; σ: proportion of cases developing smear-positive disease; θ: rate of conversion from smear-negative to smear-positive disease; r: rate of TB self-cure; d: relative detection of smear-negative TB; γ: rate of access to TB care; Se: net sensitivity of diagnostic algorithm; Sp: net specificity of diagnostic algorithm; ψ: DST coverage; η: proportion of diagnosed individuals who are linked to treatment; τ: proportion of individuals linked to care who successfully complete treatment; ξ: rate of acquisition of resistance during treatment; ι: proportion of non-progressing superinfections.

   Subscripts X: treatment-naïve; P: previously treated; S: drug-susceptible; R: MDR; g: sex; a: age; h: HIV status; k: ART duration; t: time-step. [↑](#footnote-ref-5)
5. States S: susceptible; L: latent infection; I: smear-positive TB; N: smear-negative TB.

   Parameters λ: force of infection; x: proportion protection due to previous infection against progression to active disease following reinfection; α: proportion of new infections developing primary disease; ν: reactivation rate; σ: proportion of cases developing smear-positive disease; θ: rate of conversion from smear-negative to smear-positive disease; r: rate of TB self-cure; d: relative detection of smear-negative TB; γ: rate of access to TB care; Se: net sensitivity of diagnostic algorithm; Sp: net specificity of diagnostic algorithm; ψ: DST coverage; η: proportion of diagnosed individuals who are linked to treatment; τ: proportion of individuals linked to care who successfully complete treatment; ξ: rate of acquisition of resistance during treatment; ι: proportion of non-progressing superinfections.

   Subscripts X: treatment-naïve; P: previously treated; S: drug-susceptible; R: MDR; g: sex; a: age; h: HIV status; k: ART duration; t: time-step. [↑](#footnote-ref-6)
